# Association of dengue severity with the serotype of infection: a systematic review and meta-analysis

**DOI:** 10.1016/j.eclinm.2026.104218

**Published:** 2026-09-29

**Authors:** Hadrian Jules Ang, Clare McCormack, Christl A. Donnelly, Ilaria Dorigatti

**Affiliations:** a Medical Research Council Centre for Global Infectious Disease Analysis, School of Public Health, Imperial College London, London, United Kingdom; b Department of Statistics, University of Oxford, Oxford, United Kingdom

**Keywords:** Severe dengue, Dengue haemorrhagic fever, Dengue serotype, Secondary infection, Meta-analysis

## Abstract

**Background:**

Dengue is a mosquito-borne infection caused by one of four dengue virus serotypes (DENV1-4) that circulates endemically across the tropics and subtropics. To date, there is contrasting evidence on the relationship between the serotype of infection and disease severity.

**Methods:**

We conducted a systematic review (PROSPERO, CRD42023443101) and meta-analysis to examine the association between the serotype of symptomatic infections with severe disease while accounting for both prior exposure status and geographic region, with severity defined by (i) the 1997 World Health Organization (WHO) guidelines, (ii) the 2009 WHO guidelines or (iii) hospitalisation. We searched the Global Health, Embase Classic + Embase, and Medline databases using Ovid for studies published up to 20 January 2025 and extracted case counts with matched serotype and severity information. We fitted generalised linear mixed models to estimate the proportions of severe cases, running independent analyses per severity definition, and stratifying by prior exposure-serotype combinations (e.g. primary DENV2) and geographic region (Asia versus Americas). Heterogeneity was assessed using the τ and $I^{2}$ metrics and risk of bias was assessed by adapting an established tool for risk of bias in prevalence studies.

**Findings:**

Our search yielded 4621 unique records, with 71 studies included for the meta-analysis. We found that, on average, DENV3 in Asia resulted in the highest proportion of DHF/DSS (21·00% [95% CrI: 13·02–30·44%]) and hospitalised (22·00% [95% CrI: 0·88–75·31%]) cases among symptomatic primary infections, while also causing the highest proportion of DHF/DSS among secondary infections (37·61% [95% CrI: 24·19–52·32%]). However, conditional on the same prior exposure status (primary/secondary infections), we found no statistically significant differences between serotypes across the three severity definitions. For 1997-type severity, we found some evidence of secondary infections being more severe than primary infections and found that dengue was significantly less severe in the Americas when compared to Asia, though this disparity between regions was sensitive to the inclusion of cases with unknown prior exposure status. There was no significant difference between regions for 2009-type severity and hospitalisations. The paucity of published studies from Africa limited the extent to which we could investigate dengue severity in this region.

**Interpretation:**

This study provides evidence that, according to the published literature, prior dengue exposure has a stronger association with the development of severe disease than the serotype of infection. Better characterising the local population-level immunity remains a current priority for outbreak response and healthcare demand planning.

**Funding:**

UK Medical Research Council, UK Foreign, Commonwealth & Development Office, Wellcome Trust, European Union.

**Research in context**

**Evidence before this study**

We searched PubMed for meta-analyses or systematic reviews related to the association between dengue serotypes and severe disease published before 07 May 2025 using the terms “(((Meta-analysis OR systematic review)) AND ((dengue∗ AND (severe∗ OR severity∗)) OR (DHF OR DSS OR dengue haemorrhagic fever OR dengue shock syndrome))) AND ((serotype OR serology OR ((DEN OR DENV) AND (1 OR 2 OR 3 OR 4)))).” We found 8 reviews that pooled information from multiple studies and assessed the associations between the infecting dengue serotypes and clinical presentation. Of these, a single meta-analysis conducted in 2015 assessed the effect of serotypes in combination with prior exposure, but one of the limitations of this prior analysis was the inclusion of studies sampling only hospitalised patients, which biases estimates of the probability of developing severe disease conditional on symptom onset.

**Added value of this study**

We conducted a meta-analysis to generate pooled estimates of the probability of developing severe disease and hospitalisation among symptomatic infections by serotype, prior exposure, and geographic region. In addition to broadening the study population as compared to previous studies and conducting an updated literature review, we developed a Bayesian inferential modelling approach to estimate coefficients for prior exposure-serotype combinations and geographic regions, whilst accounting for correlation between outcomes in the same study.

**Implications of all the available evidence**

Our findings contribute to better understanding the combined role of serotype, prior exposure, and geographic region in the development of severe dengue and quantify this risk among symptomatic cases reported globally. The estimates published in this study will help inform healthcare demand and capacity planning and will be used to parameterise mathematical models estimating dengue burden and the potential impact and cost-effectiveness of interventions such as vaccination and vector control under the current and changing climate.

## Introduction

Dengue virus (DENV) is a mosquito-borne flavivirus transmitted by *Aedes* mosquitoes which has been spreading throughout all World Health Organization (WHO) regions, including in Europe.^1^^,^^2^ There are an estimated 100 million annual DENV infections worldwide,^3^^,^^4^ and whilst infection can be asymptomatic or lead only to mild disease,^3^ the development of severe dengue can lead to bleeding, plasma leakage, organ impairment, and death.^1^^,^^5^

To help unify case definitions, as well as diagnostic and patient management practices, the WHO published guidelines in 1997^5^ (building on previous iterations from 1975^6^ and 1986^7^), which classified infections as dengue fever (DF) or dengue haemorrhagic fever (DHF)—with DHF being further divided into grades of increasing severity (I-IV), and DHF-III and DHF-IV also being referred to as dengue shock syndrome (DSS).^4^, ^5^, ^6^, ^7^, ^8^, ^9^ Due to concerns regarding the sensitivity and utility of this system in diagnosing severe cases,^10^, ^11^, ^12^, ^13^, ^14^ in 2009, revised WHO guidelines were published using a classification expressed in terms of dengue with/without warning signs (Dw/woWS) and severe dengue (SD).^1^ The latter was defined by the presence of either (1) severe bleeding, (2) severe plasma leakage, or (3) organ impairment.^1^^,^^10^^,^^12^ The two severity classification systems are complementary, with the clinician-driven WHO 2009 guidelines potentially more useful in triage settings, and the WHO 1997 guidelines, which define severe cases in terms of clinical and laboratory parameters such as platelet count, potentially more useful in data analyses involving comparisons across healthcare settings.

The interplay between virus genetics and the host immune response is one of the main factors that affect dengue severity.^15^, ^16^, ^17^, ^18^, ^19^, ^20^, ^21^ There are four distinct DENV serotypes (DENV1–4)^1^^,^^5^ and, whilst infection with a given serotype confers long-term homotypic and short-term heterotypic protection, secondary infection with a heterologous serotype increases the risk of severe disease.^15^, ^16^, ^17^, ^18^, ^19^, ^20^, ^21^, ^22^ This is due to the mechanism of antibody-dependent enhancement (ADE), whereby individuals with intermediate levels of pre-existing antibody titres have a higher risk of DHF compared with DENV-naïve individuals or those with high titres.^18^^,^^22^

Virus serotype has also been suggested as a factor affecting the severity of disease.^21^^,^^23^, ^24^, ^25^, ^26^, ^27^ For instance, a nationwide retrospective cohort study of in-hospital mortality in Mexico found that DENV2 and 3 resulted in significantly more fatalities than DENV1,^28^ while a cross-sectional study in India found that DENV2 more frequently led to severe dengue compared to DENV3.^29^ A study in Singapore found that after adjusting for age, gender, and prior dengue exposure, there was a higher risk of severe dengue in DENV1 infections relative to DENV2 or 3.^25^ In addition, analysis of data from patients in Rayong, Thailand found an association between DHF and DENV2 infection,^27^ while findings from other Thai regions suggested that primary DENV2 and 4 infections were less likely to progress to DHF compared to primary DENV1 and 3.^23^ Data from the long-running Paediatric Dengue Cohort and Hospital-based studies in Nicaragua indicate that, when applying WHO 1997 guidelines, DENV2 and 3 led to a larger proportion of severe cases when compared to DENV1 and 4.^26^

It has been hypothesised that genotypic variation within each serotype may also play a role in the development of severe dengue. For instance, despite an outbreak of secondary infections by the American genotype of DENV2 in Peru, there were no reports of severe dengue cases.^30^ Further analysis showed that DENV1 antibody positive sera collected from Iquitos were able to neutralise American genotype DENV2 viruses, but not the Asian genotype DENV2, which may explain the lack of severe cases reported.^31^ A study of 122 confirmed DENV1 cases from 19 hospitals in Guangzhou, China found that there was a significantly higher proportion of severe dengue among DENV1 genotype V patients compared to genotype I.^32^ An analysis of more than 20 years of dengue case and isolate data from Thailand also revealed that severity, as measured by the probability of developing symptomatic disease, was maximised when there was moderate antigenic distance between the infecting viruses at primary and secondary infections.^33^

In February 2024, the Qdenga dengue vaccine was used in a public mass vaccination campaign in a few selected Brazilian municipalities,^34^ and given that the vaccine's efficacy depends on both the serostatus before vaccination and the infecting serotype,^35^ assessing the risk of severe disease across combinations of prior exposure and infecting serotype is a current priority. Historically, rates of DHF have been observed to be higher in Asia when compared to the Americas, hypothesised to be due to differences in healthcare practice and both host and viral genetics.^36^ Because of this, it is important to also account for geographic region of infection when analysing factors associated with dengue severity.

One previous meta-analysis, published in 2015, studied the association between severe dengue and DENV serotypes, prior exposure, and geographic region.^21^ However, an important limitation of this analysis was the inclusion of studies that sampled solely from hospitalised dengue cases, which biases and overestimates the proportion of symptomatic cases developing severe disease given hospitalised patients are only a proportion of all symptomatic cases. In addition, this previous study combined severe cases classified according to WHO 1997 and 2009 guidelines, despite differences in diagnostic definitions.^21^

Here, we address these limitations and conduct a systematic review and meta-analysis (PROSPERO: CRD42023443101) to quantify the associations between the dengue serotypes, prior exposure (unknown/primary/secondary infection), and geographic regions (as a proxy for variations in circulating genotypes, host genetics, population demographics, and healthcare and hospitalisation practices) with severe disease, where severity is defined by (i) 1997 guidelines, (ii) 2009 guidelines or (iii) hospitalisation. We develop a Bayesian inferential framework to account for correlation between multiple outcomes reported in the same study and compute pooled estimates of the probability of symptomatic dengue cases progressing to severe disease for different combinations of serotype, prior exposure, and geographic region.

## Methods

A systematic review and meta-analysis was conducted according to guidelines set by the Preferred Reporting Items for Systematic Reviews and Meta-Analyses (PRISMA) statement.^37^

### Search strategy and selection criteria

We searched the Medline, Global Health, and Embase Classic + Embase databases using Ovid for all studies until 20 January 2025. Table 1 shows a summary of the search terms used. Additional studies were added to the review by searching for full paper versions of conference abstracts or poster presentations deemed to be relevant. Some relevant records from citations of screened studies were also added to the review.

**Table 1:** Summary of search strategy—terms ending with “/” are medical subject headings.

#	Search
1	((((severe OR severity) adj2 (disease∗ OR dengue∗ OR illness∗)) OR hospital∗) AND dengue∗)
2	(dengue∗ AND warning sign∗)
3	(dengue hemorrhagic fever∗ OR (DHF∗ AND dengue∗))
4	(dengue shock syndrome∗ OR (DSS∗ AND dengue∗))
5	exp Severe Dengue/
6	1 OR 2 OR 3 OR 4 OR 5
7	(serotyp∗ OR serogroup∗ OR serostrain∗ OR serovar∗ OR serological∗ OR serosurvey∗)
8	(DEN?-1 OR DEN?-2 OR DEN?-3 OR DEN?-4)
9	(D1V OR D2V OR D3V OR D4V)
10	exp Serogroup/
11	7 OR 8 OR 9 OR 10
12	6 AND 11

We included all study types published in English except other systematic or literature reviews and/or medical case reports. We searched for and extracted data from studies reporting confirmed symptomatic dengue cases with matched serotype and severity (i.e. the stated severity and serotype combination was given for the same group of people). This matching must either be explicit (e.g. “there were 10 DENV2 DF cases”) or directly inferable (e.g. “there were 10 DENV2 cases and there were no cases of DHF”). DF, Dw/woWS, or confirmed symptomatic dengue cases not fulfilling the WHO definitions were counted as non-severe dengue in the meta-analysis, but asymptomatic cases were excluded. Data on prior exposure status (primary or secondary) was extracted where available, and cases were labelled to have unknown prior exposure when the exposure status was not provided.

Serotyped cases must have been assigned a severity class based on three possible systems: (1) WHO 1997-type (using DF, DHF [I-IV], or DSS),^5^ (2) WHO 2009-type (using DwoWS, DwWS, and SD),^1^ and (3) hospitalisation (reporting whether serotyped cases were hospitalised or not). We included iterations and regional updates of these guidelines. For example, 1997-type systems include the WHO 1975,^6^ 1980,^38^ and 1986^7^ guidelines together with regional versions from the WHO Southeast Asia Regional Office (SEARO) in 1999^39^ and 2011^8^ and the Pan-American Health Organization (PAHO) in 1994.^9^ We also include the WHO 2012 guidelines under the 2009-type systems.^40^ The main outcome of interest was the proportion of symptomatic dengue cases with severe disease according to any of the three severity classification systems ([1] in 1997-type systems DHF or DSS, [2] in 2009-type systems SD only, and [3] hospitalised cases as severe) stratified by serotype and prior exposure status.

Only prior exposure-serotype combinations involving five or more dengue cases were included. For example, 48 secondary DENV3 cases and one primary DENV4 case were reported in an Indonesian cohort, so we excluded the DENV4 case but included the data for DENV3 in our analysis.^41^

We excluded studies that had not been peer-reviewed as well as conference abstracts and poster presentations. Cases identified to have non-dengue co-infections or chronic conditions (such as diabetes or hypertension) were also excluded as there is evidence that these conditions could be risk factors of severity that could bias the results.^42^ Studies explicitly sampling only patients with mild disease (excluding patients with severe disease manifestations) or only those with severe manifestations (such as studies only focussed on DHF cases) were excluded. We also excluded studies that monitored the development of severe disease only among hospitalised dengue patients as these would bias the estimates of our study upwards as some manifestations of severity are required for hospitalisation, although the criteria for hospital admission vary by country and regions. Studies that conditioned the reporting of results on the availability of the full genomic sequence of the infecting virus were also excluded, as were cases determined to be co-infected with more than one serotype. Lastly, we excluded duplicate data by matching the study period, hospital, institution, and references to named cohort studies.^20^^,^^43^

De-duplication was first conducted automatically in EndNote 21 and in Covidence. Using Covidence, titles and abstracts were screened for relevance by a single reviewer, with any remaining duplicates manually removed. Full texts were retrieved for relevant records, which were assessed by the lead reviewer. A second independent reviewer was involved when inclusion was deemed unclear by the lead reviewer. If there was conflict between the decisions of the two reviewers, a third reviewer assessed the study, and a consensus decision was made after a group discussion.

For each study included in the review, the lead reviewer extracted the following data fields using Microsoft Excel: (1) Study location, (2) study period, (3) sample size, (4) age of participants (range, interquartile range, mean, or median), (5) serotyping method, (6) severity classification system (e.g. WHO 1997), (7) prior exposure statuses found (e.g. primary, secondary, unknown), and (8) number of cases stratified by serotype, prior exposure status, and severity. Data extractions that were not clear were discussed as a group by the three reviewers.

### Data analysis

#### Pooled estimates

After extraction, data were analysed in the R statistical programming language.^44^ The goal of the meta-analysis was to generate pooled estimates of the proportion of symptomatic cases developing severe disease across prior exposure-serotype combinations and geographic regions. Pooling and data analyses were run independently for each severity classification system.

Each study i included in the analysis contributed one or more outcomes, each defined as a pair of values $\left(n_{ijkl},e_{ijkl}\right)$, where $n_{ijkl}$ is the number of dengue cases in study i in geographic region j∈{Asia,Americas}, with prior exposure status k∈{0,1,2} (unknown, primary, secondary), and serotype l∈{1,2,3,4} (DENV1 to 4), and $e_{ijkl}$ is the corresponding number of severe cases. We assumed that each study only contributed cases in a single geographic region. The only included study with an Asian and American arm was split into each geographic region for the purposes of the meta-analysis.^45^ Studies from Australia and French Polynesia were classified as Asian as they belong to the majority Asian WHO Western Pacific Region.

We assumed that $e_{ijkl}$ was binomially distributed with parameters $n_{ijkl}$ and the outcome specific probability of severe disease $\theta_{ijkl}$ which was estimated using the generalised linear mixed additive logit model given in Eqs. (1) and (2), where $\theta_{ijkl}$ was defined as a function of an intercept α (for the reference category secondary DENV2 in the Asia), a region-specific effect $\beta_{j}$, prior exposure-serotype effects $\beta_{kl}$ (to account for potential differences in primary and secondary infections), and outcome-specific random effects $\epsilon_{ijkl}$:

1 $$e_{ijkl}∼\text{Binomial}\;\left(n_{ijkl},\theta_{ijkl}\right)$$

2 $$\text{logit}\;\left(\theta_{ijkl}\right)=\alpha +{1_{j\notin \left\{Americas\right\}}\;\left(j\right)\;\beta }_{j}+1_{\left(k,l\right)\notin \left\{\left(2,2\right)\right\}}\;\left(k,l\right){\;\beta }_{kl}\;+\;\epsilon_{ijkl}$$

3 $$p_{jkl}=\text{logi}t^{-1}\;\left(\alpha +{1_{j\notin \left\{Americas\right\}\;}\left(j\right)\;\beta }_{j}+1_{\left(k,l\right)\notin \left\{\left(2,2\right)\right\}\;}\left(k,l\right){\;\beta }_{kl}\right)$$

Note that even if a scenario (j,k,l) was excluded from the fitting due to a lack of studies, we were still able to compute an estimate of the proportion of severe cases, $p_{jkl}$, if corresponding estimates of $\beta_{j}$ and $\beta_{kl}$ were available (Eq. (3)). The choice of using a random effects versus a fixed effects model was pre-specified, as dengue studies would presumably come from a wide range of settings and involve different populations.

If study i contributed outcomes to a single scenario, then $\epsilon_{ijkl}∼N\left(0,\sigma_{jkl}\right)$, where $\sigma_{jkl}$ is the scenario-specific standard deviation of the random effects. If study i contributed data to more than one scenario then $\epsilon_{ij}$ is a vector of random effects with dimensions equal to the number of scenarios study i contributes to, and we assumed that the random effects had covariance $\Sigma_{ij}$ for study i in region j as shown in Eq. (4) (see SI: Computing Random Effects for more details):

4 $$\epsilon_{ij}∼\text{MvNormal}\;\left(0,\Sigma_{ij}\right)$$

The model was fitted running four independent chains with 10 000 iterations each (5000 for warm-up). Six main parameter groups were estimated: intercept α, model coefficients $\beta_{j}$ and $\beta_{kl}$, random effect standard deviations $\sigma_{jkl}$, random effects $\epsilon_{ijkl}$, and region-specific correlation matrices for random effects in outcomes from the same study $P_{j}$, which are used to compute covariance matrices $\Sigma_{ij}$. Statistical significance of region- and prior exposure-serotype effects was assessed through the 95% credible intervals (CrI) of the posterior distributions of odds ratios (ORs). See Table S1 for full information on parameter prior distributions. The model was implemented using the Stan probabilistic programming language (R v4·5·3, RStan v2·32·7, Stan v2·32·2) with Asia-Secondary-DENV2 chosen as the reference scenario.^46^

#### Heterogeneity estimates

For each scenario included in the analysis, we estimated the between-study standard deviation in true effects $\tau_{jkl}$ and reconstructed the corresponding proportion of variation due to study heterogeneity rather than sampling error, $I_{jkl}^{2}$, through simulation (see SI: Estimating Heterogeneity).^47^ We defined $I_{jkl}^{2}\;>75\%$ to represent substantial heterogeneity, as suggested in the Cochrane handbook.^48^ In addition, we also computed prediction intervals (PIs) through simulation (see SI: Computing Prediction Intervals).

#### Sensitivity analysis and validation

To validate our results, we ran two sensitivity analyses to compare our pooled estimates against well-known sources. First, we compared the results of the main analysis of 1997-type severity with pooled estimates from a subset of well-known, multi-year cohorts. We fitted a simplified model using Secondary-DENV2 as the reference class with data from Narvaez et al.^26^ from Nicaragua, and Stephens et al.^49^ and Fried et al.^50^ from Thailand. Compared to the main model (Eqs. 1, 2)), we excluded the region effect $\beta_{j}$ and the random effects $\epsilon_{ijkl}$, while relaxing the restriction on outcomes requiring at least five cases for inclusion in the analysis, resulting in equations 5, 6).

5 $$\text{logit}\left(\theta_{ikl}\right)=\alpha \;+\;1_{\left(k,l\right)\notin \left\{\left(2,2\right)\right\}}\left(k,l\right)\beta_{kl}$$

6 $$e_{ikl}∼\text{Binomial}\left(n_{ikl},\theta_{ikl}\right)$$

In addition, we validated our pooled estimates of the proportion of cases with unknown prior exposure requiring hospitalisation by comparing them to the proportion of virologically confirmed dengue (VCD) hospitalisations reported in the placebo arm of the TAK-003 clinical trial at 57 months since the first dose.^51^

To check for potential masking of serotype-specific effects, we also ran the analysis across all three severity definitions while excluding cases where prior exposure was unknown. We also ran a leave-one-out sensitivity analysis for 1997-type severity, individually excluding the three largest studies with known prior exposure, to check the robustness of the serotype-specific estimates of dengue severity to the most influential studies. Additionally, we ran a sensitivity analysis with stricter de-duplication criteria, excluding studies that could be potential duplicates based on authors and institutions, even if duplication could not be confirmed (e.g. when study period is not explicitly given).

### Bias assessment

We assessed study-specific risk of bias using an adapted version of the tool proposed by Hoy et al.^52^ for assessing the risk of bias in prevalence studies (see Table S2). This tool was applied framing the current review as a meta-analysis of the prevalence of severe disease among symptomatic dengue cases. The tool includes 10 questions including validity of sampling, case definition, and diagnostic instruments used, with each rated as 1 (low risk of bias) or 0 (high risk of bias). We included two summary measures, one based on the sum of responses across the 10 questions (with 0–4 being high risk, 5–7 moderate risk, and 8–10 low risk of bias), and a subjective assessment of overall risk of bias (low, moderate, or high). Each study was assessed by two independent reviewers, with studies having high risk of bias excluded.

We also assessed small study effects, which could hint at the presence of publication bias, using a modified version of Peters' Regression^53^—using fitted random effects $\epsilon_{ijkl}$ as the dependent variable (weighted by inverse standard deviation 1/ $\sigma_{jkl}$), and inverse sample size $1/n_{ijkl}$ as the independent variable to answer the question: “do smaller studies systematically report smaller or larger proportions of severe dengue compared to the scenario average”. As a sensitivity analysis we also ran standard Peters' regression using the *meta* package (v8.5.0).^54^ Due to correlations between the effect size we are studying and the standard error, we opted not to use Egger's Regression, as this could lead to false positives (see SI: Assessing Small Study Effects for more details).^53^

### Role of the funding source

Study funders had no role in study design, data extraction and analysis, or preparation of the manuscript.

## Results

### Study selection

The literature search yielded 8533 records, of which 4621 were screened following de-duplication (Fig. 1). The full texts of 1577 out of 1581 relevant records were successfully retrieved. An inclusion/exclusion decision was made for 1388 studies by a single reviewer, while 189 studies were further assessed by a second independent review, with conflicts resolved as a group of three.

**Fig. 1:**
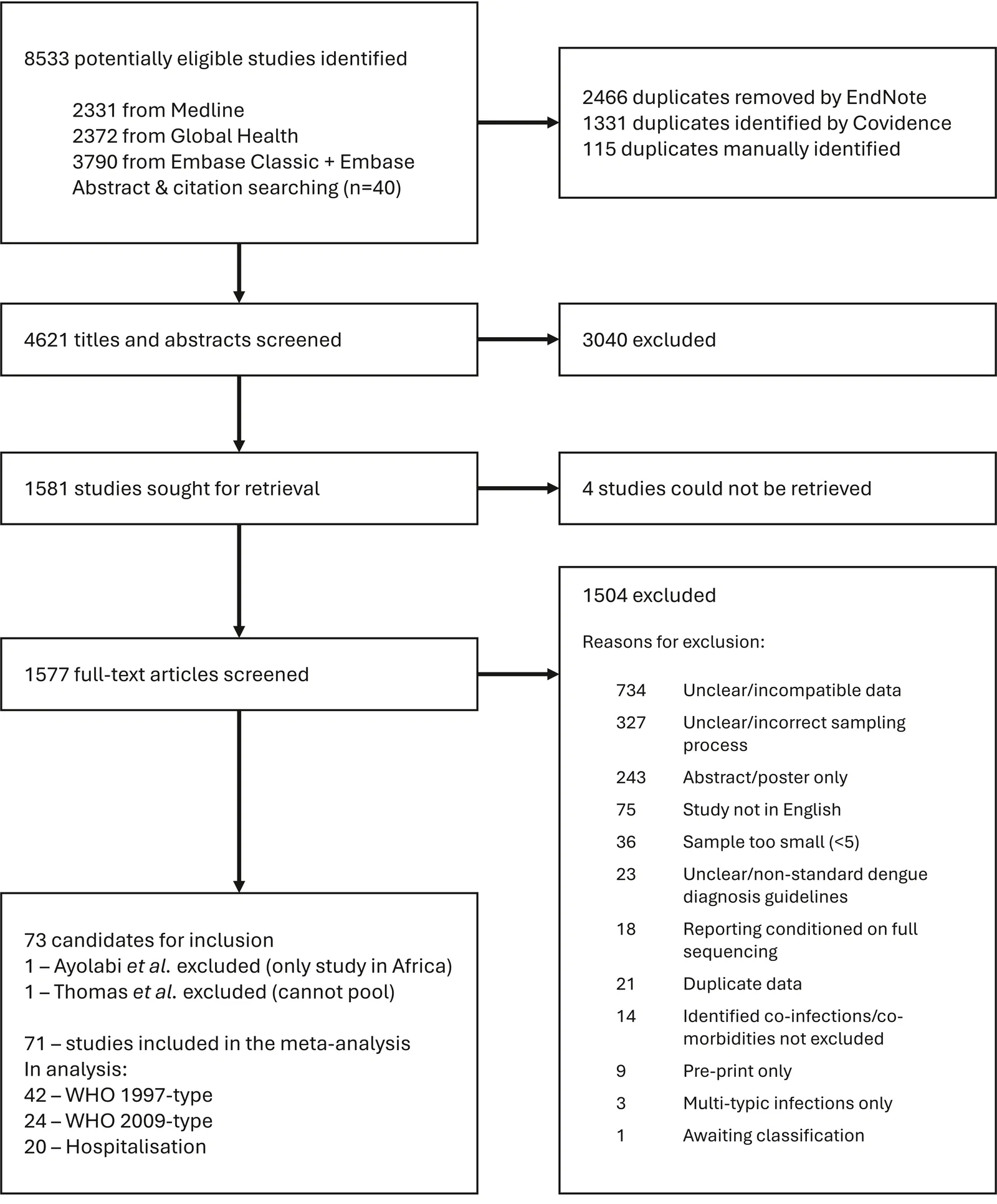
PRISMA flow diagram of study selection.

Following full assessment, 1504 studies were excluded and 73 passed review criteria. One excluded study is awaiting classification as the data could not be verified, though its publisher has been contacted for clarification. Two studies passing the criteria were not included in the meta-analysis due to a lack of sufficient studies for pooling in their particular scenarios: Ayolabi et al. was the only study included from Africa,^55^ while Thomas et al. was the only study reporting on hospitalisations due to primary DENV2 and 4 in the Americas.^56^ Thus 71 studies were included in the analysis. A full list of included studies together with a summary of extracted characteristics is presented in Tables S3–S5, while Figure S1 shows a breakdown of study locations by severity definition used.

All included studies were assessed for risk of bias, with both branches of the study from Dussart et al.^45^ (separate Asian and American cohorts) assessed separately. Most studies had a low risk of bias, 49/72 and 50/72 when using the subjective summary ratings, and 55/72 and 60/72 for reviewers 1 and 2, respectively when using sum-based summary ratings (Fig. 2). All other studies were assessed to have moderate risk of bias, with none classified as high risk using either summary criteria, due to the strict inclusion/exclusion criteria applied. Therefore, no additional sensitivity analyses on study exclusions for bias assessment were conducted (see Tables S6 and S7 for a breakdown of bias assessment results per reviewer).

**Fig. 2:**
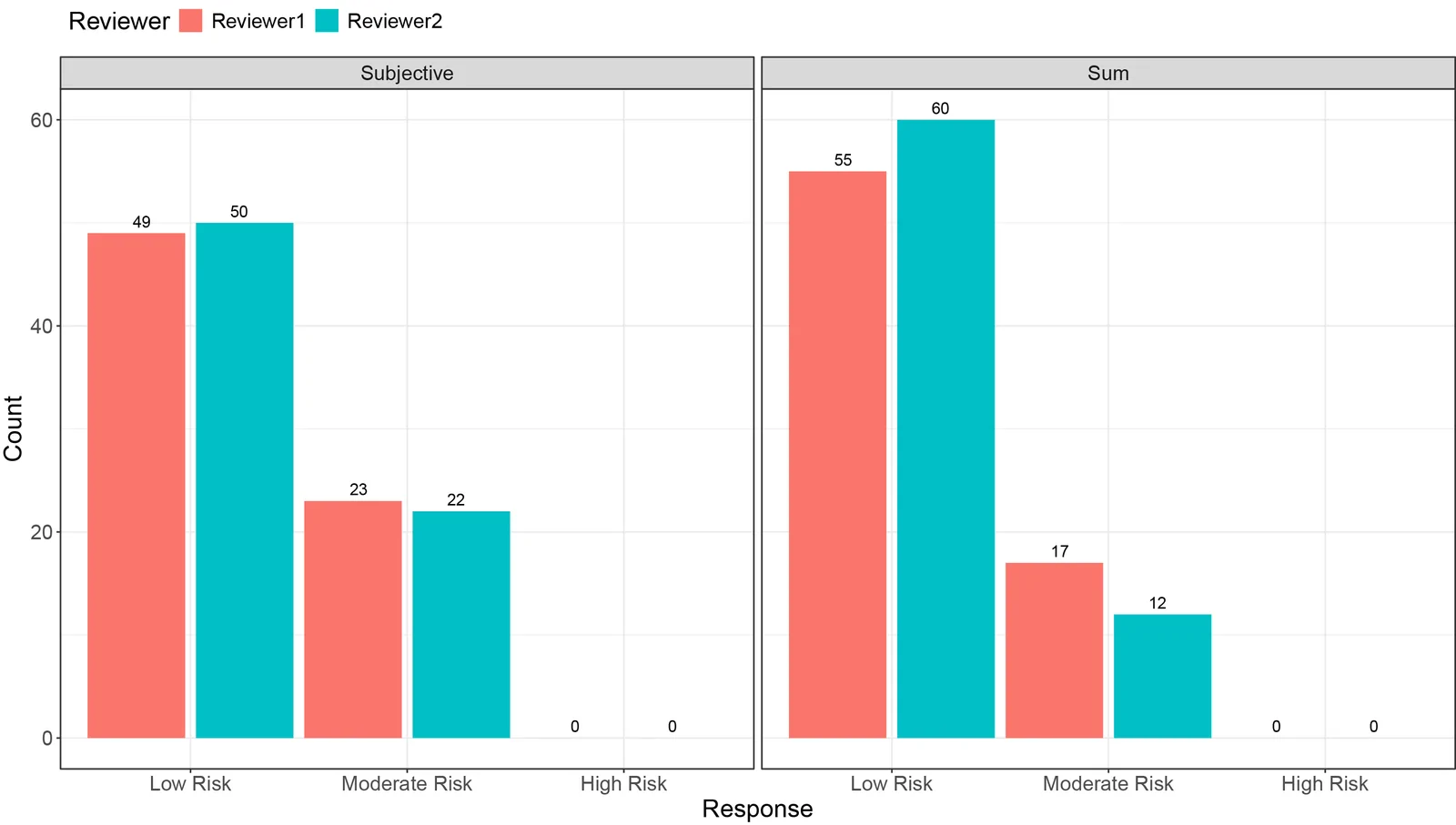
Number of studies assessed as low, moderate, or high risk of bias for subjective and sum-based summary ratings across two independent reviewers.

One of the most prevalent potential sources of bias was related to non-response (where a response was defined as patient inclusion in the study through dengue diagnostics), as resource constraints often meant that studies could only test a subset of patients presenting with dengue symptoms. It was not always explicit whether those tested were representative of all symptomatic patients as there was less information on those excluded, but this was not deemed sufficient to classify studies as high risk of bias.

We did not find evidence of any significant small study effects (see SI: Assessing Small Study Effects and Tables S8 and S9 for more details).

### Meta-analysis

All model parameters across independent runs for the three severity definitions converged with $\hat{R}$ <1·01 and with sufficient sample sizes (effective sample size >5000 for all relevant parameters), as shown in Tables S10–S12. Trace plots are given in Figures S2–S10, and Table S13 shows the number of studies contributing to each parameter estimate. Individual study estimates of the proportion of severe cases, $\theta_{ijkl}$ are given in Figures S11–S59.

#### Primary infections

For both 1997-type severity and hospitalisation, the most severe primary infection on average was DENV3 in Asia (proportion with DHF/DSS: 21·00% [95% CrI: 13·02–30·44%], proportion hospitalised: 22·00% [95% CrI: 0·88–75·31%]), though the limited hospitalisation data generated large uncertainty around the central estimate (Fig. 3). On the other hand, primary DENV2 in the Americas was the least severe on average for both definitions of severity (proportion DHF/DSS: 4·08% [95% CrI: 0·24–12·88%], proportion hospitalised: 6·47% [95% CrI: 0·20–34·60%]). In fact, Americas-Primary-DENV2 was, on average, the least severe scenario for both 1997-type severity and hospitalisation.

**Fig. 3:**
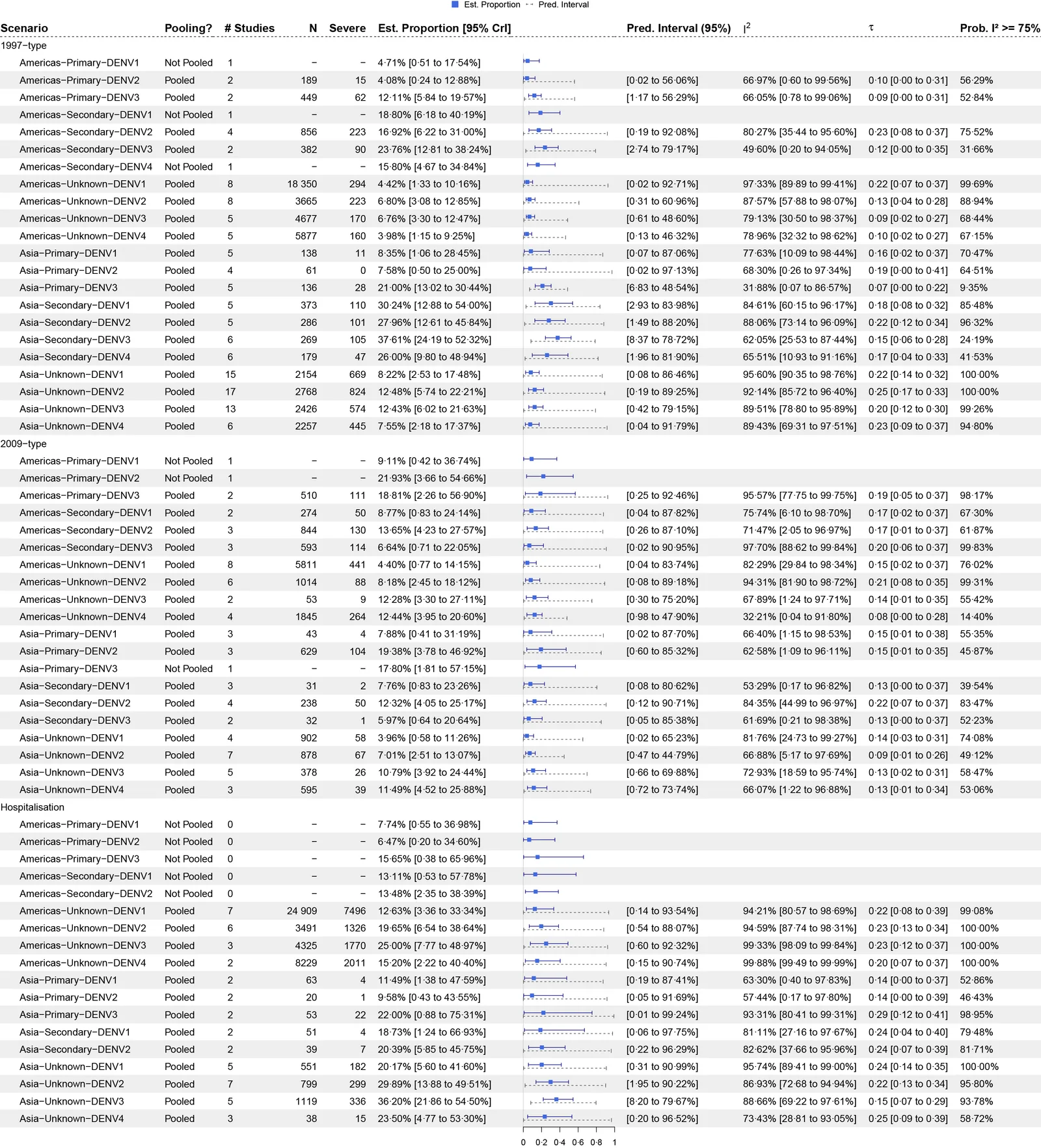
Forest plot of pooled estimates of the proportion of severe cases ($p_{jkl}$) and corresponding prediction intervals according to 1997-type, 2009-type, and hospitalisation-based severity with estimated heterogeneity τ and $I^{2}$ and their 95% CrIs. The last column indicates the probability that $I^{2}\geq$ 75% based on its posterior distribution. Scenarios labelled “Not Pooled” had proportions computed solely using estimated $\beta_{j}$ and $\beta_{kl}$ values.

In contrast to 1997-type severity and hospitalisation, when using 2009-type severity, DENV2 in the Americas was, on average, the most severe primary infection (proportion SD: 21·93% [95% CrI: 3·66–54·66%], while DENV1 in Asia was the least severe (proportion SD: 7·88% [95% CrI: 0·41–31·19%]). However, a lack of studies in the Americas reporting 2009-type severity meant that estimates for the prior exposure-serotype effect $\left(\beta_{kl}\right)$ for primary DENV1 and 2 were based only on studies in Asia (3 in each scenario).

Comparing pairs of prior exposure-serotype effects (all combinations of estimated $\beta_{kl}$), we did not find a statistically significant difference between serotypes in primary infections across any of the three severity classification systems (Figs. 4 and 5, and Figure S60). For example, with 1997-type severity, even the difference between the least and most severe primary infections, DENV2 versus DENV3, was not statistically significant (OR: 0·35 [95% CrI: 0·02–1·28]). However, it is worth noting that due to limited data we were unable to generate estimates for primary DENV4 across any of the severity classification systems, thus we could not compare it against the other serotypes.

#### Secondary infections

Just as in primary infections, the most severe serotype in secondary infections for 1997-type severity was DENV3 (proportion DHF/DSS in Asia: 37·61% [95% CrI: 24·19–52·32%], in Americas: 23·76% [95% CrI: 12·81 to 38·24%]), with Asia-Secondary-DENV3 also being the most severe 1997-type scenario overall. Conversely, we found that, on average, DENV4 led to the lowest proportion of DHF/DSS among secondary infections (Asia: 26·00% [95% CrI: 9·80–48·94%], Americas: 15·80% [95% CrI: 4·67–34·84%]).

For hospitalisation, the only serotypes we could analyse for secondary infections were DENV1 and 2, with DENV2 leading to a higher proportion of hospitalisations on average (Asia: 20·39% [95% CrI: 5·85–45·75%], Americas: 13·48% [95% CrI: 2·35–38·39%]). Similarly, we found DENV2 to be the most severe secondary infection for 2009-type severity (proportion SD 12·32% [95% CrI: 4·05–25·17%] in Asia, and 13·65% [95% CrI: 4·23–27·57%] in Americas).

Mirroring our findings in primary infections, we found no statistically significant difference between serotypes in secondary infections (Figs. 4 and 5, and Figure S60). Using 1997-type severity, even comparing the most and least severe secondary infections, DENV3 and 4, the difference was not significant (OR: 2·16 [95% CrI: 0·56–6·05]).

**Fig. 4:**
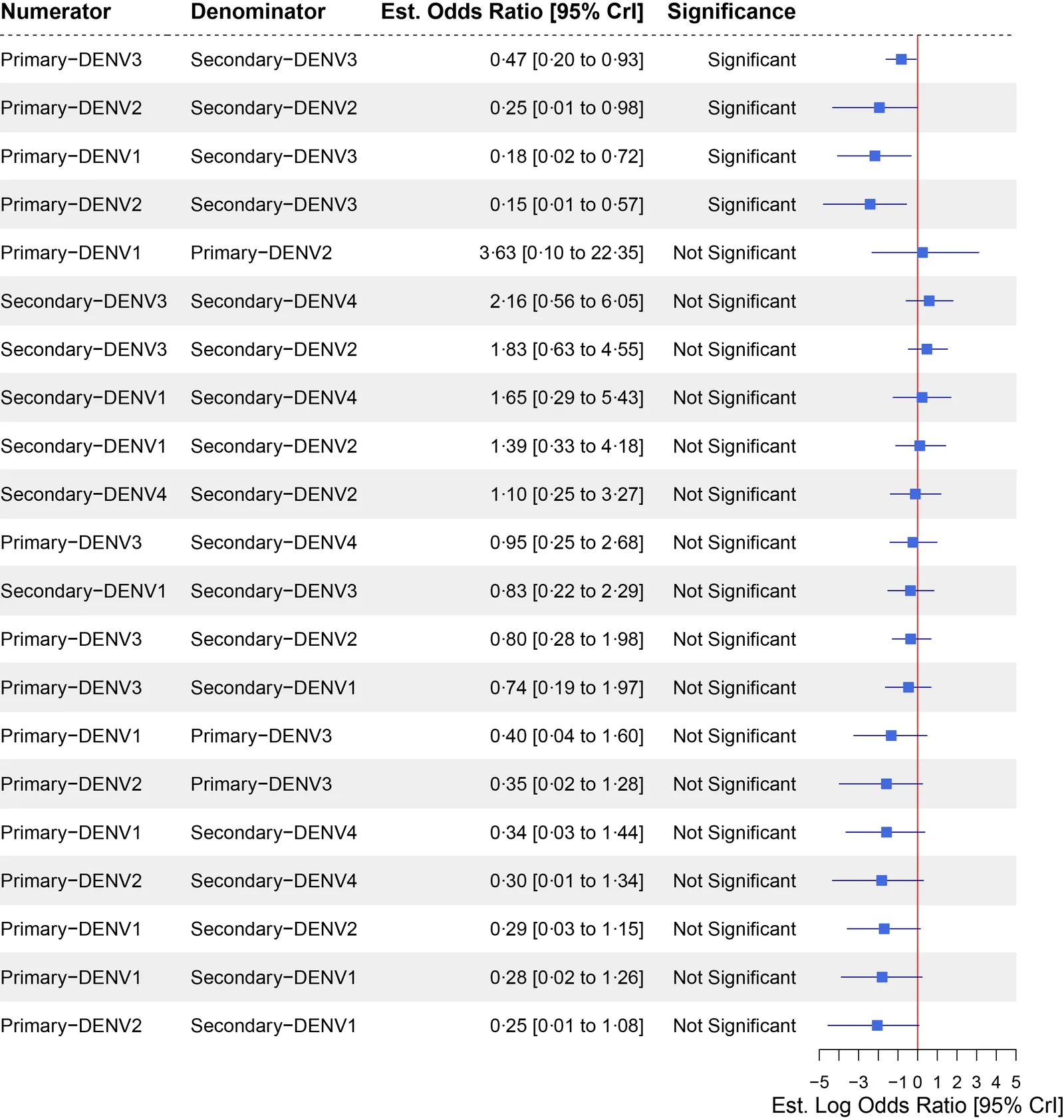
Estimated log odds ratios for 1997-type severity between different prior exposure-serotype combinations (with odds ratios in the table). Estimates where the 95% CrI of the OR is entirely below or above 1 were deemed to be statistically significant.

**Fig. 5:**
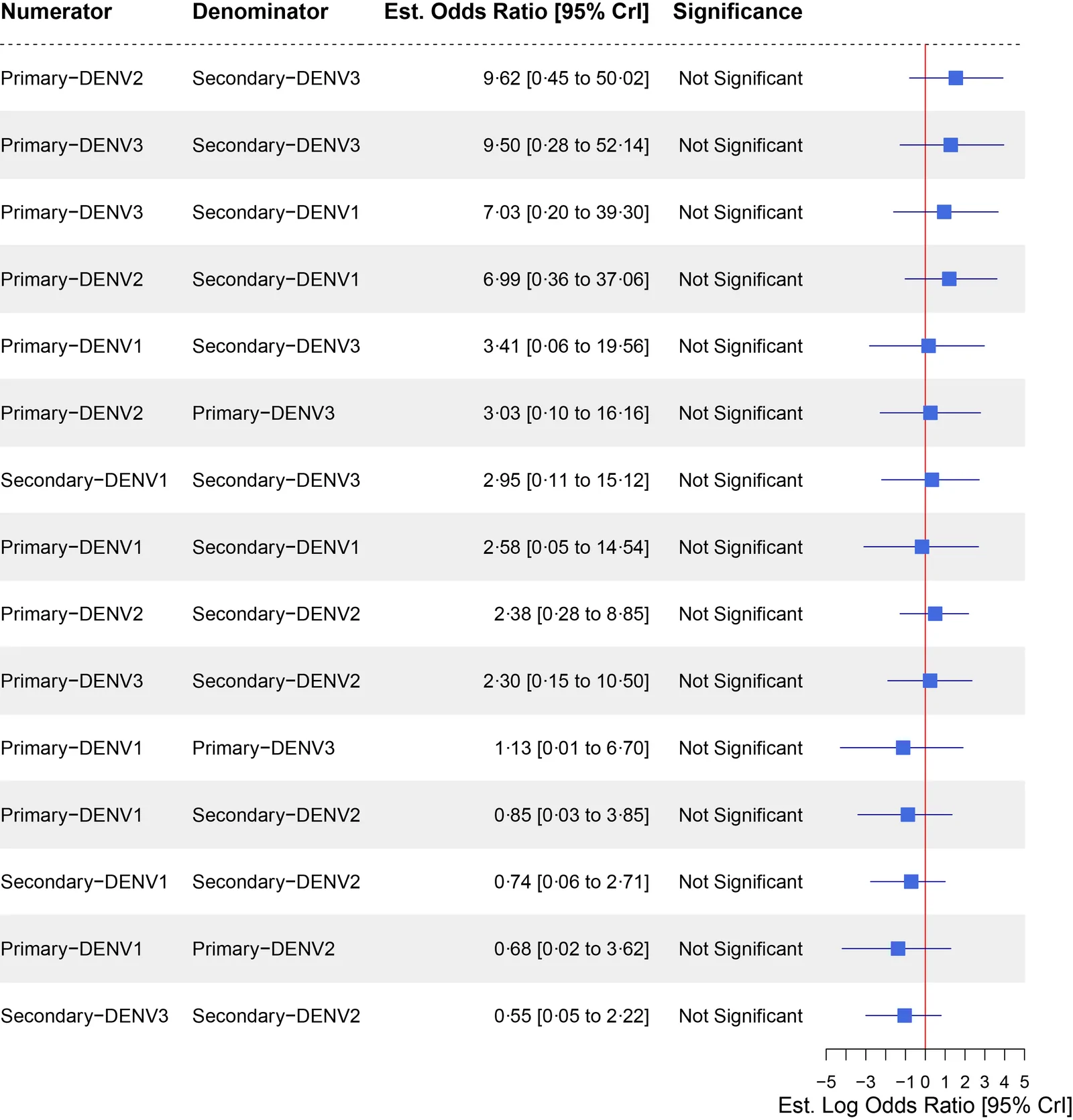
Estimated log odds ratios for 2009-type severity between different prior exposure-serotype combinations (with odds ratios in the table). Estimates where the 95% CrI of the OR is entirely below or above 1 were deemed to be statistically significant.

We also compared different prior exposure-serotype combinations and found significant differences between some primary and secondary infections when using 1997-type severity. For example, we found that secondary DENV3 was significantly more severe than primary DENV1, 2, and 3 (with estimates for primary DENV4 unavailable due to a lack of data). Comparing primary and secondary infections of the same serotype, estimated ORs were similar for DENV1 (OR: 0·28 [95% CrI: 0·02–1·26]) and DENV2 (OR: 0·25 [95% CrI: 0·01–0·98]), which were both smaller than for DENV3 (OR: 0·47 [95% CrI: 0·20–0·93]), though the difference between primary and secondary DENV1 was not statistically significant.

For both 2009-type severity (Fig. 5) and hospitalisation (Figure S2), we found no statistically significant differences across different prior exposure-serotype combinations. Thus, in contrast to 1997-type severity, we found no evidence of secondary infections being more severe than primary ones when using the 2009-type classification.

#### Geographic differences

Using 1997-type severity, we found that the odds of a dengue case being severe in the Americas was on average around half of that in Asia, and this reduced severity was statistically significant (OR: 0·53 [95% CrI: 0·27–0·91]). While dengue was also less severe on average in the Americas for hospitalisations (OR: 0·64 [95% CrI: 0·17–1·67]), it was more severe in the Americas for 2009-type severity (OR: 1·26 [95% CrI: 0·40–2·70]). However, the differences between regions for 2009-type severity and hospitalisations were not statistically significant.

#### Heterogeneity

For 1997-type severity, there was substantial heterogeneity (mean $I^{2}$ >75%) across all scenarios where prior exposure status was unknown (Fig. 3). When prior exposure status was known, heterogeneity was generally lower but still substantial for primary and secondary DENV1 in Asia, and secondary DENV2 in both Asia and the Americas.

Heterogeneity within scenarios in Asia with unknown prior exposure status was generally lower for 2009-type severity than 1997-type severity. However, among scenarios with known prior exposure status, there was still substantial heterogeneity among scenarios in the Americas except for secondary DENV2. Heterogeneity was lower in Asia, though still substantial for secondary DENV2, though no scenario where prior exposure was known had more than 4 studies. For hospitalisations, heterogeneity was substantial in all included scenarios except primary DENV1 and 2 in Asia, which only involved two studies each, and DENV4 in Asia with unknown prior exposure.

This amount of heterogeneity in many scenarios combined with small numbers of studies also meant that PIs were correspondingly wide. For example, in the analysis of 1997-type severity, the PI for primary DENV2 infection in the Americas was (95% PI: 0·02–56·06%), while the mean effect sat in a narrower range (4·08% [95% CrI: 0·24–12·88%]).

However, we found that the widths of these PIs were sensitive to the prior distributions used. We ran a prior sensitivity analysis setting the prior distributions for α, $\beta_{j}$, $\beta_{kl}$, and $\sigma_{jkl}$ to N(0,1) with a [0,∞) constraint for $\sigma_{jkl}$ (instead of the main analysis prior distributions in Table S1). With this change in prior distributions, the mean estimated proportions were generally similar (e.g. 5·07% [95% CrI: 1·20–11·05%] for primary DENV2 in the Americas), but the PIs were much narrower (95% PI: 0·33–23·68%), with the upper bound in the sensitivity analysis being around half that in the main analysis (23·68% versus 56·06%), as shown in Figure S61. Given that there were only two studies in this scenario, the between-study heterogeneity is not well-estimated, thus reporting the wider PIs, as we have chosen here, is more apt. It is worth noting that even with this change in prior distributions, findings were consistent with the main analysis, as there were no significant differences between serotypes when conditioning on the same prior exposure status (Figures S62–S64).

### Sensitivity analyses

Most central estimates from the cohort study sensitivity analysis sat between central Asian and American estimates from the main analysis, as expected, given the analysis includes one American and two Asian studies (Figure S65). However, we found that the proportion of severe secondary DENV4 cases in the sensitivity analysis (10·97% [95% CrI: 7·92–14·49%]) was smaller than the proportions from the main analysis (Asia: 26·00% [95% CrI: 9·80–48·94%], Americas: 15·80% [95% CrI: 4·67–34·84%]). In contrast to the main analysis, the pooled estimates obtained from the cohort studies showed significant differences between serotypes, with DENV2 and 3 being the most severe secondary infections, and DENV3 being the most severe primary infection (Figure S66). See SI: Cohort Study Sensitivity Analysis for the full results of the cohort study sensitivity analysis.

We found that the proportions of hospitalised cases observed during the Takeda vaccine trial^51^ generally fell between the central estimates from the main analysis, except for DENV3, where the observed proportion of hospitalisations during the trial (15·93% [95% CI: 9·72–24·00%] including Sri Lanka and 12·96% [95% CI: 7·27–20·79%] excluding it) were smaller than the central estimates from the main analysis (36·20% [95% CrI: 21·86–54·50%] in Asia and 25·00% [95% CrI: 7·77–48·97%] in the Americas). See SI: Comparison with Clinical Trial Data for full results (Figure S67).

When excluding cases with unknown prior exposure status (Figure S68), we found that there were still no significant differences between serotypes when conditioning on the same prior exposure status across all three severity definitions (Figure S69). However, the difference in 1997-type severity between cases in the Americas and Asia was no longer statistically significant (OR: 0·76 [95% CrI: 0·32–1·75]) (see SI: Excluding Unknown Prior Exposure for full results).

We re-ran the analysis of 1997-type severity individually excluding the three studies with the largest sample sizes of cases with known prior exposure status (Figures S70–S72), namely the study in Nicaragua by Narvaez et al.,^26^ and two studies in Thailand by Sabchareon et al.,^57^ and Fried et al.,^50^ respectively. After exclusion of data from Narvaez et al., primary DENV2 infection was estimated to be significantly less severe than primary DENV3 (OR: 0·14 [95% CrI: 0·00–0·74], see Figure S73), however, this also meant that estimates of these two prior exposure-serotype combinations were based solely on studies from Asia, as there were no longer enough studies to generate pooled estimates for primary DENV2 and 3 in the Americas. Individual exclusion of the other two Thai studies did not change any conclusions regarding serotype- or region-specific differences (see SI: Excluding Influential Studies for full results).

Finally, our sensitivity analysis with stricter de-duplication criteria resulted in the exclusion of six more studies for 1997-type severity, and two studies for 2009-type severity, with no changes for the analysis of hospitalisation (see Table S14 for a list of studies excluded for the sensitivity analysis). Though means of pooled estimates shifted slightly (Figure S74), consistent with our main analysis of 1997-type severity, DENV3 in Asia was, on average, the most severe primary (18·72% [95% CrI: 10·08–29·28%]) and secondary (33·80% [95% CrI: 18·47–50·88%]) infection, and there remained no statistically significant difference between serotypes when controlling for the same prior exposure status in 1997-type or 2009-type severity (Figures S75 and S76). However, the difference in 1997-type severity between the Americas and Asia was no longer statistically significant (OR: 0·71 [95% CrI: 0·35–1·32]), aligning with the findings of our sensitivity analysis excluding all cases with unknown prior exposure (likely due in part to 4/6 of the additional exclusions being studies only reporting unknown prior exposure). See SI: Stricter De-duplication for more details.

## Discussion

In this study, we conducted a systematic review and meta-analysis generating pooled estimates of the proportion of symptomatic dengue cases progressing to severe disease across combinations of geographic region, prior exposure status, and serotype of infection. We ran this analysis for three dengue severity classification systems, WHO 1997-type, WHO 2009-type, and hospitalisation, with each being an independent model run. Conditional on the same prior exposure status, we did not find a statistically significant difference between serotypes across any of the three severity classification systems. Results for 1997-type severity indicate a stronger link between severe disease and prior exposure, rather than any specific infecting serotype.

For 1997-type severity, secondary DENV2 and 3 were both significantly more severe than primary infections of the same type, with secondary DENV3 also being significantly more severe than primary infections of any other serotype. This is consistent with prior reviews and analyses, which have found that secondary infections are associated with a greater probability of severe disease.^21^^,^^42^^,^^58^, ^59^, ^60^ We also found that dengue was significantly less severe in the Americas compared to Asia (OR: 0·53 [95% CrI: 0·27–0·91]) when applying 1997-type severity, though this finding did not hold when cases with unknown prior exposure were excluded from the analysis or when stricter de-duplication (which mostly excluded cases with unknown prior exposure) was applied, suggesting that differences in underlying demographics or population-level immunity profiles may be contributing to the apparent severity of dengue in Asia. It has also been hypothesised that this difference between regions could be due to genetic variation in circulating strains^30^ or differences in healthcare practice, with the WHO 1997 guidelines requiring clinical tests such as those for changes in haematocrit which may not always be conducted.^36^

A sensitivity analysis examining data only from the historical Thai^49^^,^^50^ and Nicaraguan^26^ cohorts showed that, similar to the main analysis, DENV3 was the most severe primary and secondary infection, and that there was a significant difference in severity between primary and secondary DENV2. However, in contrast to the main analysis, this sensitivity analysis also revealed significant differences between serotypes even when conditioning on the same prior exposure status, with secondary DENV2 and 3 being the most severe.

The estimates of the proportion of symptomatic DENV1, 2, and 4 cases hospitalised with unknown prior exposure aligned with the observed proportions of VCD cases hospitalised during the TAK-003 clinical trial.^51^^,^^61^ However, for DENV3, the pooled mean estimates obtained in this study were approximately 2–3 fold larger than the mean observed proportion from the trial. It is worth noting though that most of the DENV3 cases observed during the trial were from the Philippines, so the difference may be driven by the hospitalisation practices of the different countries.^61^

In fact, regional differences in healthcare practice combined with genetic differences in circulating viruses likely contributed to the considerable heterogeneity found in some of the scenarios we analysed. Other potential factors contributing to this heterogeneity include the varying proportions of primary and secondary infections among study participants, variability in patient age,^42^ different study designs, different exposures to other flaviviruses such as Zika, which has been found to enhance dengue disease severity,^62^^,^^63^ and potentially JEV vaccination.^64^

Prior systematic reviews and meta-analyses have reported on the association of dengue serotypes and prior exposure with severe disease.^21^^,^^42^^,^^58^, ^59^, ^60^^,^^65^, ^66^, ^67^ For example, outbreaks where DENV2 was the dominant serotype have been estimated to have the highest pooled mortality rates^58^ and an analysis of dengue trends in Pakistan showed that DENV2 and 3 were associated with more severe disease than DENV1 and 4.^66^ However, with the exception of Soo et al.^21^ who conducted a meta-analysis of dengue severity by serotype in primary and secondary infections with published studies until July 2015, previous studies analysed serotype and prior exposure separately. Our work differs from Soo et al.^21^ by including newer studies, using a different analytic approach, and modifying inclusion/exclusion criteria. Combining 1997-type and 2009-type severity, Soo et al. found that secondary DENV4 in Southeast Asia was, on average, the most severe, with approximately 53% of cases being DHF, DSS or SD.^21^ In general, we estimated lower proportions of severe cases, peaking at a pooled DHF/DSS proportion of 37·61% (95% CrI: 24·19–52·32%) for secondary DENV3 in Asia. A possible explanation for this difference is that Soo et al. included studies sampling only hospitalised patients and excluded those reporting no severe cases, which could have biased the estimates upwards.^21^

We found that our estimates of proportions of severe cases differed according to the definition of severity. This difference was in part due to a lack of data for 2009-type severity and hospitalisation, which is one of the limitations of this meta-analysis. For example, using 1997-type severity, secondary DENV3 in Asia led to the highest proportion of DHF/DSS: 37·61% (95% CrI: 24·19–52·32%), on average (6 studies, 269 cases). Estimates for secondary DENV3 in Asia for 2009-type severity were much smaller (5·97% [95% CrI: 0·64–20·64%]) and involved fewer studies (2 studies, 32 cases), while secondary DENV3 estimates were not available for hospitalisation. The lack of data also meant that we were unable to generate estimates of severity for primary DENV4 infections across any of the severity classification systems, though results from the sensitivity analysis run on the cohort studies indicate primary DENV4 as less severe than the other prior exposure-serotype combinations.

We were also unable to include (and thus control for) patient age in our meta-analysis due to a lack of data matching serotype, prior exposure status, severity, and age for the same group of patients in the published literature. However, a meta-analysis by Sangkaew et al. found that younger children and older adults were more prone to severe dengue disease than older children and younger adults, respectively.^42^ Aside from this, our analysis also lacks data on the sequence of infecting serotypes (i.e. serotype at primary infection for cases with known serotype of secondary infection), which has been suggested to influence clinical outcomes.^33^ These data limitations meant that there remained substantial residual heterogeneity in many scenarios. In future studies, it would be useful to report individual patient data including age and the history of infections to allow for a fuller exploration of the associations between these different factors, though the reconstruction of sequential infections from virological and serological data is a current challenge for dengue surveillance and modelling.

Our study is also limited to two geographic regions (Asia and the Americas) given there was an insufficient number of studies in South Asia to divide Asia into sub-regions. There was also a paucity of studies in Africa, with only a single study from Nigeria reporting on 11 serotyped dengue cases, none of which were severe after applying WHO 2009 guidelines.^55^ Studies from a greater geographic range could contribute to a better understanding of the potentially confounding effects of differing healthcare practices and variations in virus or host genetics.

A strength of this study is that we fitted our model to data for different serotypes, prior exposure statuses, and geographic regions simultaneously. We also allowed for correlations in random effects for outcomes coming from the same study, while also modelling a separate effect for each prior exposure-serotype combination $\left(\beta_{kl}\right)$ and geographic region $\left(\beta_{j}\right)$. This approach allowed us to leverage data from Asia to inform estimates for the Americas and vice versa, making it possible to compute estimates for scenarios that did not have enough studies for a meta-analysis. Additionally, our sensitivity analyses showed that our pooled estimates generally align with empirical findings from well-known cohort studies and vaccine clinical trials.

In conclusion, our results suggest a lack of significant serotype-specific differences in severity across all classifications tested in this study, but a strong association between prior exposure and severe disease. From a clinical perspective, this lack of evidence for a serotype-specific link to severity means that the serotype of infection taken alone and independently of other patient parameters, cannot be used to predict the clinical course of a dengue illness nor be used in triage settings. This also means that, for outbreak response planning, information on the dominant circulating serotype taken independently from historical transmission dynamics is unlikely to determine healthcare and hospital demand, highlighting the importance of seroprevalence surveys in reconstructing population immunity profiles. The documented serotype-specific differences in the efficacy of dengue vaccines^35^—both licensed and in development, antiviral drugs^68^ and vector control interventions^69^ raise questions on the potential public health and economic impact of interventions against the four dengue serotypes. As such, the results presented in this study contribute to synthesising and characterising the published evidence on the severity of the different serotypes, which can be used to parameterise mathematical models of dengue transmission, thereby allowing for refined estimates of the potential impact and cost-effectiveness of interventions.

## Contributors

The study was conceptualised by ID. HJA, ID, CM curated the data. HJA, ID, CM, CAD performed the formal analysis. Funding was acquired by ID. CAD, HJA, ID, CM designed the methodology. Software was developed by HJA. The underlying data was verified by HJA, ID, CM. HJA, ID generated the visuals. The original draft of the manuscript was written by HJA, ID and was reviewed and edited by HJA, ID, CM, CAD. The project was supervised by ID, CM. All authors had full access to the study data, have reviewed and approved the final version of the manuscript.

## Data sharing statement

Data preparation, analysis, and model code together with extracted data are available online at https://github.com/hadrianang/denv-sev-meta.

## Declaration of interests

HJA, CM, and ID received funding from the UK Medical Research Council (MRC), UK Foreign Commonwealth & Development Office (UK FCDO), European Union, and the EDCTP2 programme through funding for the MRC Centre for Global Infectious Disease Analysis. HJA received funding from the UK MRC for PhD studies and the World Health Organization (WHO) South-East Asia Regional Office in support of previous travel. CM received grants from the Medical Research Foundation (MRF) UK. CAD received funding from the UK National Institute for Health Research (NIHR), and Oxford Martin School, University of Oxford, received grants or contracts from the UK MRC and UK FCDO, received royalties or licenses from Cambridge University Press and Chapman & Hall/CRC Press, received consulting fees from UK Defra via UK Animal and Plant Health Agency (APHA) and the Lampard Inquiry, received support for attending meetings and/or travel from Australian Wildlife Management Society, and has had trustee roles in the Royal Society, Royal Statistical Society, and St Peter's College, Oxford. CAD also declares a position at the WHO R&D Blueprint, and payments from Science Foundation Ireland, Singapore National Environment Agency, and Swiss National Science Foundation. ID received research funding from the Wellcome Trust and MRF, consulting fees from the WHO, is a current member of Wellcome's Population and Public Health Discovery Advisory Group Committee and has acted as an Advisory Board member for Bavarian Nordic.
