# Supplementary material for: Association of dengue severity with the serotype of infection: a systematic review and meta-analysis
Source: eClinicalMedicine. 2026 Sep 29;100:104218. doi: 10.1016/j.eclinm.2026.104218 (PMC13654564; doi:10.1016/j.eclinm.2026.104218)
Supplement: Supplementary file 1 [file mmc1.docx]

Supplementary Information

Association of Dengue Severity with the Serotype of Infection: a systematic review and meta-analysis

Hadrian Jules Ang, Clare McCormack, Christl A. Donnelly, Ilaria Dorigatti

Contents

[Methods	2](#_Toc238576833)

[Computing Random Effects	2](#_Toc238576834)

[Estimating Heterogeneity	3](#_Toc238576835)

[Parameter Prior Distributions	4](#_Toc238576836)

[Bias Assessment Tool	4](#_Toc238576837)

[Results	11](#_Toc238576838)

[Summary of Included Studies	11](#_Toc238576839)

[Bias Assessment Results	18](#_Toc238576840)

[Assessing Small Study Effects	24](#_Toc238576841)

[Trace Plots and Parameter Estimates (1997-type Severity)	25](#_Toc238576842)

[Trace Plots and Parameter Estimates (2009-type Severity)	34](#_Toc238576843)

[Trace Plots and Parameter Estimates (Hospitalisation)	42](#_Toc238576844)

[Number of Studies Contributing to Parameter Estimates	48](#_Toc238576845)

[Individual Study Results per Scenario (1997-type Severity)	48](#_Toc238576846)

[Individual Study Results per Scenario (2009-type Severity)	57](#_Toc238576847)

[Individual Study Results per Scenario (Hospitalisation)	61](#_Toc238576848)

[Hospitalisation Results	65](#_Toc238576849)

[Prior Sensitivity Analysis	65](#_Toc238576850)

[Cohort Study Sensitivity Analysis	69](#_Toc238576851)

[Comparison with Clinical Trial Data	71](#_Toc238576852)

[Excluding Unknown Prior Exposure	73](#_Toc238576853)

[Excluding Influential Studies	76](#_Toc238576854)

[Stricter De-duplication	81](#_Toc238576855)

[References	84](#_Toc238576856)

# Methods

## Computing Random Effects

We fit outcome-specific random effects $\epsilon_{ijk\mathcal{l}}$ corresponding to the random effect for study $i$, contributing data for region $j$, prior exposure status $k$, and serotype $\mathcal{l}$. If study $i$ contributes data for only a single scenario, then $\epsilon_{ijk\mathcal{l}}\sim N(0,\sigma_{jk\mathcal{l}})$ where $\sigma_{jk\mathcal{l}}$ is the standard deviation of random effects for scenario $\left( j,k\mathcal{,l} \right)$. We compute $\epsilon_{ijk\mathcal{l}}$ by sampling values $\tilde{\epsilon}_{ijk\mathcal{l}}\sim N(0,1)$ and setting

$$\epsilon_{ijk\mathcal{l}}=\sigma_{jk\mathcal{l}}\tilde{\epsilon}_{ijk\mathcal{l}}$$

Eq. S1

However, if study $i$ contributes data to more than one scenario, we assume $\epsilon_{ij}\sim\mathrm{MvNormal}\left( 0,\Sigma_{ij} \right)$, where $\epsilon_{ij}$ is the vector of random effects for each outcome from study $i$, and $\Sigma_{ij}$ is the covariance matrix for study $i$ in region $j$. The covariance matrix $\Sigma_{ij}$ is constructed using an appropriate principal sub-matrix of the region-specific correlation matrix $P_{j}$ (taking rows and columns corresponding to prior exposure-serotype combinations for region $j$ that study $i$ is contributing data to) and scenario-specific standard deviations $\sigma_{jk\mathcal{l}}$, with further details provided below.

Let $R_{j}$ be the set of scenarios included in the analysis for region $j$ (those that have at least two studies included in the analysis) and let $S_{ij}\subseteq R_{j}$ be the set of scenarios included in the analysis that study $i$ in region $j$ contributes data to. The region-specific correlation matrix $P_{j}\in\mathbb{R}^{\left| R_{j} \right|\times\left| R_{j} \right|}$ has a row and column corresponding to each prior exposure-serotype combination for region $j$. We define the study-specific correlation matrix $P_{ij}\in\mathbb{R}^{\left| S_{ij} \right|\times\left| S_{ij} \right|}$ as the sub-matrix of $P_{j}$which contains rows and columns of $P_{j}$ corresponding only to scenarios which study $i$ contributes data to.

Letting $z_{i}=\left( \sigma_{jk\mathcal{l}} \right)_{\left( j,k\mathcal{,l} \right)\in S_{ij}}$ denote the vector of all standard deviations corresponding to scenarios that study $i$ contributes data to, we compute the vector of random effects for study $i$ ($\epsilon_{ij})$as

$$\epsilon_{ij}=z_{i}\odot\left( \mathrm{Chol}\left( P_{ij} \right)\times\tilde{\epsilon}_{ij} \right)$$

Eq. S2

where $\odot$ is the Hadamard product (element-wise multiplication), $\mathrm{Chol}(P_{ij})$ denotes the lower triangular matrix of the Cholesky decomposition of $P_{ij}$, and $\tilde{\epsilon}_{ij}\sim N\left( 0,1 \right)$denotes initial values of the random effect. This is equivalent to having $\epsilon_{ij}\sim\mathrm{MvNormal}\left( 0, \Sigma_{ij} \right)$ where

$$\Sigma_{ij}=diag\left( z_{i} \right)\times P_{ij}\times diag\left( z_{i} \right)$$

Eq. S3

and $diag(m)$ for some vector $m$ returns a diagonal matrix with the elements of $m$ on the main diagonal.

## Estimating Heterogeneity

We present two metrics related to heterogeneity in the main text (Figure 3): the between-study standard deviation $\tau$ and the proportion of the total observed variance due to heterogeneity, rather than sampling error, $I^{2}$. We estimated both values by simulation.

Let $\mu_{jk\mathcal{l}}$ be the logit scale sum of fixed effects for scenario $\left( j, k\mathcal{, l} \right),$

$$\mu_{jk\mathcal{l}}=\alpha+{\mathbb{1}_{j\notin\{Americas\}}(j)\beta}_{j}+\mathbb{1}_{\left( k\mathcal{,l} \right)\notin\{\left( 2,2 \right)\}}\left( k\mathcal{,l} \right)\beta_{k\mathcal{l}}$$

Eq. S4

where $\beta_{j}$ is the region-specific effect and $\beta_{k\mathcal{l}}$ the prior exposure-serotype effect. For each posterior sample $\chi\in\{1, \ldots, 20 000\}$, we have values $\mu_{jk\mathcal{l}}^{(\chi)}$ and $\sigma_{jk\mathcal{l}}^{\left( \chi\right)}$ (standard deviation of random effects in scenario [$j, k\mathcal{,l}$]). To compute simulated effect sizes $\tilde{\theta}_{jk\mathcal{l}}^{\left( \chi\right)}$ for each posterior sample $\chi$ , we draw 1000 samples $\tilde{\varepsilon}_{jk\mathcal{l}}^{\left( \chi\right)}\sim N\left( 0, \sigma_{jk\mathcal{l}}^{\left( \chi\right)} \right)$, each a random effect for a simulated study, and set

$$\tilde{\theta}_{jk\mathcal{l}}^{\left( \chi\right)}=logit^{-1}\left( \mu_{jk\mathcal{l}}^{\left( \chi\right)}+\tilde{\varepsilon}_{jk\mathcal{l}}^{\left( \chi\right)} \right)\in\mathbb{R}^{1000}$$

Eq. S5

We then compute $\tau_{jk\mathcal{l,}\left( \chi\right)}^{2}=Var\left( \tilde{\theta}_{jk\mathcal{l}}^{\left( \chi\right)} \right)$. This process is repeated for each posterior sample $\chi$ and we report the mean and credible interval for $\tau_{jk\mathcal{l}}$.

We compute the sampling variance for observed proportions $\left( \frac{e_{ijk\mathcal{l}}}{n_{ijk\mathcal{l}}} \right)$ as

$$v_{ijk\mathcal{l}}^{\left( \chi\right)}=Var\left( \frac{e_{ijk\mathcal{l}}}{n_{ijk\mathcal{l}}} \right)=\frac{p_{jk\mathcal{l}}^{\left( \chi\right)}\left( 1-p_{jk\mathcal{l}}^{\left( \chi\right)} \right)}{n_{ijk\mathcal{l}}}$$

Eq. S6

This is derived from the variance of a binomial distribution, where the probability of success is $p_{jk\mathcal{l}}^{(\chi)}$, the pooled result for scenario $\left( j,k\mathcal{,l} \right)$, which excludes random effects. Let $T_{jk\mathcal{l}}=\left\{ i \right|n_{ijk\mathcal{l}}>0\}$ be the set of all studies contributing data for scenario $\left( j,k\mathcal{,l} \right)$. To represent the typical amount of sampling variance in the scenario, we compute the mean sampling variance of studies contributing data

$$\bar{v}_{jk\mathcal{l}}^{\left( \chi\right)}=\frac{1}{\left| T_{jk\mathcal{l}} \right|}\sum_{i\in T_{jk\mathcal{l}}} v_{ijk\mathcal{l}}^{\left( \chi\right)}$$

Eq. S7

We then compute the proportion of between-study variance due to heterogeneity rather than sampling error ($I^{2}$), as follows:

$$I_{jk\mathcal{l,}\left( \chi\right)}^{2}=\frac{\tau_{jk\mathcal{l,}\left( \chi\right)}^{2}}{\tau_{jk\mathcal{l,}\left( \chi\right)}^{2}+\bar{v}_{jk\mathcal{l}}^{\left( \chi\right)}}$$

Eq. S8

We report the mean and credible interval for $I_{jk\mathcal{l}}^{2}$.

## Computing Prediction Intervals

Let $\mu_{jk\mathcal{l}}^{\left( \chi\right)}$ be the logit scale sum of fixed effects for scenario $\left( j,k\mathcal{,l} \right)$ for posterior sample $\chi\in\{1,\ldots,20 000\}$ (see Eq. S4). We computed samples from the posterior predictive distribution for the given scenario as

$$q_{jk\mathcal{l}}^{\left( \chi\right)}=\mathrm{logi}t^{-1}\left( \mu_{jk\mathcal{l}}^{\left( \chi\right)}+\zeta_{jk\mathcal{l}}^{\left( \chi\right)} \right)$$

Eq. S9

where $\zeta_{jk\mathcal{l}}^{\left( \chi\right)}$ is a simulated random effect drawn randomly from $N\left( 0,\sigma_{jk\mathcal{l}}^{\left( \chi\right)} \right)$ and $\sigma_{jk\mathcal{l}}^{\left( \chi\right)}$ is the corresponding posterior sample for the standard deviation of random effects. We then presented the 2·5^th^ and 97·5^th^ (the 95% CrI) of $q_{jk\mathcal{l}}$ as the 95% prediction interval.

## Parameter Prior Distributions

| **Parameter** | **Description** | **Prior Distribution** | **Constraint** |
| --- | --- | --- | --- |
| $\alpha$ | Intercept (reference value for secondary DENV2 in the Asia) | $N\left( 0,2 \right)$ | NA |
| $\beta_{j}$ | Region $(j)$ specific effect versus reference class | $N\left( 0,2 \right)$ | NA |
| $\beta_{k\mathcal{l}}$ | Prior exposure ($k$) – serotype ($\mathcal{l}$) specific effect versus reference class | $N\left( 0,2 \right)$ | NA |
| $\sigma_{jk\mathcal{l}}$ | Standard deviation of random effects. | $N\left( 0.5, 2 \right)$ | $[0,\infty)$ |
| $\tilde{\epsilon}_{ijk\mathcal{l}}$ | Initial values of the random effects, which are then transformed depending on the number of outcomes coming from the same study $i$. If only a single outcome is given, then $\epsilon_{ijk\mathcal{l}}\sim N\left( 0,\sigma_{jk\mathcal{l}} \right)$  but $\epsilon_{ij}\sim MvNormal\left( 0, \Sigma_{ij} \right)$ when there are multiple outcomes. See the section Appendix: Computing Random Effects for more details. | $N\left( 0,1 \right)$ | NA |
| $P_{j}$ | Region $(j)$-specific correlation matrix for random effects in outcomes coming from the same study. | $\mathrm{LKJCorr}(2)$ | $[-1,1]$  Must be valid correlation matrix |

Table S1: Parameter descriptions and prior distributions.

## Bias Assessment Tool

| **#** | **Question** | **Criteria** | **Notes** |
| --- | --- | --- | --- |
| **External Validity** | | | |
| 1 | Was the study’s target population **a close representation** of the population of symptomatic dengue cases in relation to relevant variables, e.g. age, sex, occupation? | - **Yes (LOW RISK)**: The study’s target population was a close representation of the population of symptomatic dengue cases. - **No (HIGH RISK)**: The study’s target population was clearly NOT representative of the population of symptomatic dengue cases. | We modify Q1 from the original stated by Hoy *et al.,^1^* as rather than assessing the prevalence of some condition in a national population, we are assessing the prevalence of severe dengue among symptomatic cases in general. The focus here is how well the **target population** (group being sampled/studied) represents symptomatic dengue cases. For example, studies only taking cases from narrow age ranges (such as paediatric cohorts) could potentially have a high risk of bias based on these criteria.  We deem studies with target populations presenting to hospitals or healthcare centres to have a low risk of bias. While these populations may not be completely representative of all symptomatic dengue cases, this is the best that can be done in many settings and can be deemed to be a “close representation.” |
| 2 | Was the sampling frame **a true or close representation** of the target population? | - **Yes (LOW RISK):** The sampling frame was a **true or close** representation of the target population. - **No (HIGH RISK):** The sampling frame was NOT a **true or close** representation of the target population. | In Q1, we focussed on how well the **target population** matched the overall population of symptomatic dengue cases. Here, in Q2, we look at the sampling frame of the study (i.e. the patient list, database, or group the study samples from). The goal is to assess how well the study samples the group they claim to study.  Just as in Q1, if a study is looking into symptomatic dengue, we deem the sampling of only those presenting to healthcare centres, hospitals (and other disease surveillance points) to be low risk of bias as this is a close enough representation of symptomatic dengue (i.e. authors/study organisers do not necessarily have to setup community-based cohorts/sampling to be considered low risk of bias).  For example, if a study is looking into disease severity among patients presenting to hospitals with dengue-like symptoms or suspected dengue, this question asks if a representative set was sampled from among these patients or if there were other extra conditions which could bias results (e.g. only including those bleeding). For our purposes, we count sampling only from patients presenting with fever + 1 or 2 dengue symptoms (e.g. arthralgia, myalgia, bleeding) as low risk of bias for healthcare institution-based observational studies. |
| 3 | Was some form of **random selection** used to select the sample, OR was census undertaken? | - **Yes (LOW RISK):** A census was undertaken OR some form of random selection was used to select the sample (e.g. simple random sampling, stratified random sampling, cluster random sampling, systematic sampling). - **No (HIGH RISK):** A census was NOT undertaken, AND some form of random selection was NOT used to select the sample. | Note that Q3 is in relation to the sampling frame selected for the target population (not necessarily the entire population of symptomatic dengue). As defined by Hoy *et al.*,^1^ a census would involve every unit in the sampling frame, while surveys would include only a part of it. If doing a survey, some form of random sampling should be used to reduce the risk of bias.  For example, if the target population was individuals presenting to hospital with fever + 1 or 2 dengue symptoms, then a census would involve testing (and attempting to serotype) all patients who present during the study period. If testing everyone was not possible (e.g. due to limited access to serotyping methods such as RT-PCR), then random sampling should be used to select those to be tested. We count serotyping only those who were tested positive for some preliminary test (such as NS1) as low risk of bias if all or a representative random sample of participants underwent preliminary testing. |
| 4 | Was the likelihood of **non-response bias minimal**? | - **Yes (LOW RISK):** The response rate for the study was >= 75%, OR an analysis was performed that showed no significant difference in relevant characteristics between responders and non-responders. - **No (HIGH RISK):** The response rate was <75%, and if any analysis comparing responders and non-responders was done, it showed a significant difference in relevant characteristics between responders and non-responders (or no such analysis was done). | We define a response to be the successful collection and testing of a sample from an individual. If preliminary testing is involved, the 75% threshold mentioned in the criteria is applied to each test in the sequence leading to the serotyping method. For example, in a study serotyping only those NS1+, at least 75% of patients involved in the study should be tested by NS1 and at least 75% of those NS1+ should have serotyping attempted. Risk of bias could potentially be mitigated if authors presented an analysis showing that there was no significant difference between tested and non-tested groups in terms of demographics and clinical presentation. |
| **Internal Validity** | | | |
| 5 | Were data collected **directly from the subjects** (as opposed to a proxy)? | - **Yes (LOW RISK):** All data were collected directly from the subjects. - **No (HIGH RISK):** In some instances, data were collected from a proxy (such as a serum bank or database). | We take this to mean that study authors were involved in or had full knowledge regarding the collection of samples. There could be a risk of bias for studies that only received samples from a serum bank or had no part in collection (e.g. only presenting values from a surveillance system). |
| 6 | Was an acceptable case definition used in the study? | - **Yes (LOW RISK):** An acceptable case definition was used. - **No (HIGH RISK):** An acceptable case definition was NOT used. | Acceptable case definitions include those from WHO guidelines (including their iterations or regional updates), such as those published in 1997^2^ or 2009.^3^ We also accept hospitalisation status as a valid severity classification if dengue was serotyped and confirmed using standard testing.  If prior exposure status was also given, standard criteria such as WHO guidelines should be used to define secondary versus primary infections. |
| 7 | Was the instrument that measured the parameter of interest **reliable and valid?** | - **Yes (LOW RISK):** A reliable and valid instrument was used to measure the parameter of interest. - **No (HIGH RISK):** The study instrument had NOT been shown to have reliability or validity. | Dengue testing / serotyping should ideally be carried out through serotype-specific RT-PCR tests or viral culture + immunofluorescence assay (IFA) with type-specific monoclonal antibodies. While serotype could potentially be inferred from antibody patterns, such as when using plaque reduction neutralisation tests (PRNT), this could be biased due to cross-reactivity.  If prior exposure statuses were also given, valid methods such as IgM/IgG tests with antibody ratios presented, haemagglutination inhibition (HI), or PRNT should be used. |
| 8 | Was the same mode of data collection used for all subjects? | - **Yes (LOW RISK):** The same mode of data collection was used for all subjects. - **No (HIGH RISK):** The same mode of data collection was NOT used for all subjects. | Was the same testing methodology used among study participants? If some were serotyped using RT-PCR, while others were serotyped using viral culture + IFA, this could introduce some bias. Ideally, all participants should go through the same testing pipeline (including decision making on testing). For example, there would be a high risk of bias if a subset of patients required preliminary testing (such as through NS1 tests), while others were tested by RT-PCR directly.  If only one testing method was used or multiple methods were used on all participants, then risk of bias is low. |
| 9 | Was the **length of the shortest prevalence period** for the parameter of interest appropriate? | - **Yes (LOW RISK):** The shortest prevalence period for the parameter of interest was appropriate. - **No (HIGH RISK):** The shortest prevalence period for the parameter of interest was not appropriate. | By prevalence period here, we mean the period dengue can be clinically and virologically diagnosed. We adapt this question to include two things related to sampling and the dengue disease phase: (1) were patients sampled during acute/febrile phase, and (2) were they then observed over the disease course to see if they would develop severe symptoms (i.e. recording the highest severity class reached by a patient, rather than just at a snapshot at one point in time)?  For example, studies sampling patients from a large time window from disease onset (e.g. 1-14 days) may be biased as serotyping may fail for those too far into the course of disease. Studies that also do not observe the whole course of disease have a risk of bias, as symptoms may be mild early on then develop to be severe. |
| 10 | Were the **numerator(s) and the denominator(s)** for the parameter of interest appropriate? | - **Yes (LOW RISK):** The paper presented appropriate numerator(s) AND denominator(s) for the parameter of interest. - **No (HIGH RISK):** The paper did present numerator(s) AND denominator(s) for the parameter of interest but one or more of these were inappropriate. | The numerator should be the number of severe cases (based on WHO 1997, WHO 2009, or Hospitalisation) for a given geographic region and prior exposure status (unknown, primary, secondary), while the denominator is the corresponding total number of dengue cases. |
| **Summary** | | | |
| 11 | Summary item on overall risk of bias | - 2 – Low risk of bias: further research is very unlikely to change our confidence in the estimate - 1 – Moderate risk of bias: further research is likely to have an important impact on our confidence in the estimate and may change the estimate - 0 – High risk of bias: Further research is very likely to have an important impact on our confidence in the estimate and is likely to change the estimate |  |

Table S2: Bias assessment tool for prevalence of severe disease among symptomatic dengue patients adapted from the original tool from Hoy *et al.^1^*

# Results

## Summary of Included Studies

Studies were included from 10 locations across the Americas and 16 from Asia (Figure S1). Overall, 42 studies were included in the meta-analysis for 1997-type severity, 24 for 2009-type severity, and 20 for hospitalisations. There were some overlaps between groups since some studies gave data for more than one severity class system. Thailand, and Brazil were the locations with the most data, though there was a lack of studies from Thailand using 2009-type severity classification.

734 studies were excluded due to unclear/incompatible data, which was mostly due to studies either lacking serotype or severity information or lacking a clear matching between them. 327 studies were excluded due to unclear or unsuitable sampling for this review (such as by sampling only hospitalised inpatients).

**Figure S1**: Number of included studies per location coloured by the severity classification system used. Studies that gave results for more than one severity definition were counted once for each definition used.

| **Study** | **Location** | **Study Period** | **Age (Statistic, Population: Value)** | **Serotyping Method** | **Prior Exposure Status** | **Severity Class Guideline** | **Severe** | **N** |
| --- | --- | --- | --- | --- | --- | --- | --- | --- |
| Poeranto 2016^4^ | Indonesia | 1995-1999 | Range, Inclusion criteria: 4-13 years | RT-PCR, Virus isolation, fluorescence staining, PRNT50 | Primary, Secondary | 1997 | 27 | 171 |
| Osmali 2007^5^ | Malaysia | 04/2005-04/2006 | Mean $\pm$ SD, Confirmed dengue cases: 38·35 $\pm$ 14·14 years | RT-PCR, Virus isolation, indirect immunofluorescence antibody test | Unknown | 1997 | 0 | 9 |
| Burattini 2016^6^ | Brazil | 2000-2014 | No summary statistic | Not Mentioned | Unknown | 1997 (Brazil) | 593 | 22 595 |
| Sharp 2013^7^ | Puerto Rico | 01/2010-12/2010 | Median (Range), Lab positive dengue cases: 18 (0·08 to 102) years | RT-PCR | Unknown | 1997 | 187 | 7428 |
| Myint 2006^8^ | Thailand | NA | Mean $\pm$ SD, DF: 7·8 $\pm$3·1, DHF: 8·7$\pm$ 3·2 years | RT-PCR, Virus Isolation | Unknown | 1997 | 55 | 111 |
| Saha 2016^9^ | India | 08/2008-12/2012 | No summary statistic | RT-PCR | Unknown | 2009** | 15 | 433 |
| Dewi 2014^10^ | Indonesia | 2009-2010 | Inclusion criteria: $\geq$14 years | RT-PCR, Virus Isolation | Unknown | 1997 | 3 | 11 |
| Montoya 2003^11^ | Peru | 05/2000-05/2001 | No summary statistic | RT-PCR, Virus isolation, indirect fluorescent antibody test | Unknown | 1997 | 14 | 276 |
| Dewi 2021^12^ | Indonesia | 03/2010-12/2011 | Inclusion criteria: >14 years | RT-PCR, Virus Isolation | Unknown | 1997 | 25 | 54 |
| Wardhani 2017^13^ | Indonesia | 02/2012-08/2012 | Range, Dengue suspected patients: 0-60 years | RT-PCR, Virus Isolation | Unknown, Secondary | SEARO 2011 | 44 | 65 |
| Harris 2000^14^ | Nicaragua | 07/1998-12/1998 | Mean (Range), Lab-confirmed dengue: 15·0 (0-84) years | RT-PCR, Virus Isolation, IFA | Unknown | 1997 | 12 | 122 |
| Kerdpanich 2021^15^ | Thailand | 2002-2018 | Median (Range), KPP Hospital: 14 (0·08-95) years, PMK Hospital: 11 (0·08 to 76) years | RT-PCR, Virus isolation | Unknown | 1997 | 2110 | 5414 |
| Guilarde 2008^16^ | Brazil | 01/2005-07/2005 | Mean $\pm$ SD, DF: 32·4 $\pm$ 12·7, Intermediate DF/DHF: 33·0 $\pm$ 13·9 years, DHF: 32·7 $\pm$ 14·4 years | RT-PCR, Virus isolation, indirect fluorescent antibody test | Unknown | 1997 (Brazil) | 8 | 56 |
| WHO 1987^17^ | Puerto Rico | 08/1986-12/1986 | No summary statistic | Virus isolation | Unknown | 1986* | 6 | 331 |
| Sabchareon 2012^18^ | Thailand | 2006-2009 | Mean (Range, Median, Mode), Serologically confirmed dengue cases: 9·4 (4-14, 10, 11) years | RT-PCR | Primary, Secondary | 1997 | 36 | 332 |
| Yung 2015^19^ | Singapore | 05/2005-12/2011 | Median (Range), DENV-1: 35 (19-74) years, DENV-2: 37 (18-77) years, DENV-3: 39 (19-87) years | RT-PCR | Unknown | 1997 | 82 | 451 |
| Dumre 2017^20^ | Nepal | 08/2010-12/2010 | Median (IQR), Dengue patients: 29·5 (21·3-40·0) years, DENV-1: 29·0 (17·8-40·0) years, DENV-2: 28·0 (15·5-36·0) years, DENV-4: 25·0 (20·0-37·5) years | RT-PCR | Unknown | 1997 | 15 | 83 |
| Chakravarti 2010^21^ | India | 07/2008-12/2008 | Mean (Range), Dengue patients: 29 (2-65) years | RT-PCR | Unknown | 1997 | 12 | 20 |
| Rao 2018^22^ | India | 01/2013-12/2013 | No summary statistic | RT-PCR | Unknown | 2015 | 11 | 656 |
| Stephens 2002^23^ | Thailand | NA | Range, Inclusion criteria: 3-14 years | RT-PCR, Virus isolation | Primary, Secondary | 1997 | 112 | 257 |
| Reyes 2010^24^ | Nicaragua | 2006-2007 | Mean (Range), Index cases: 8 (4-12) years, Post-enrolment dengue: 23 (11-67) years, Pre-enrolment: 34 (range not given) years | RT-PCR, Virus isolation | Secondary | 1997* | 0 | 7 |
| Feres 2006^25^ | Brazil | 1994-2003 | Range, Dengue cases: 1-60 years | RT-PCR, Virus isolation | Unknown | 1997 | 7 | 713 |
| Sharma 2020^26^ | India | 01/2016-12/2017 | Median (Range), Dengue infections: 33·94 (9-75) years | RT-PCR | Unknown | 1997 | 0 | 106 |
| Thomas 2010^27^ | Martinique | NA | Median (IQR), Dengue patients: 30 (23) years | RT-PCR | Unknown | 1997 | 16 | 66 |
| Fried 2010^28^ | Thailand | 1994-1997; 1999-2002;2004-2006 | Mean $\pm$ SD, DF: 8·5 $\pm$ 3·0 years, DHF: 8·7 $\pm$ 3·1 years | RT-PCR, Virus isolation | Primary, Secondary | 1997 | 166 | 448 |
| Falconar 2012^29^ | Colombia | 2007-2007 | Mean (Range), Confirmed dengue infections: 8·1 (1-27) years | Virus isolation, Immunofluorescent assay | Secondary | 1997 | 5 | 14 |
| Ocazionez 2006^30^ | Colombia | 1998-2004 | Mean $\pm$ SD, DENV-1: 22·1 $\pm$ 28·1 years, DENV-2: 25·1 $\pm$ 18·3 years, DENV-3: 26·0 $\pm$ 16·1 years, DENV-4: 21·1 $\pm$ 4·7 years | RSS-PCR, Virus isolation, indirect fluorescent antibody test | Primary, Secondary, Unknown | 1980 | 24 | 122 |
| Kao 2016^31^ | Taiwan | 07/2009-02/2010 | No summary statistics | RT-PCR, Virus isolation | Unknown | 1997 | 9 | 295 |
| Kosasih 2016^32^ | Indonesia | 08/2000-06/2004; 09/2006-05/2009 | Mean (Range; SD), Study participants: 35·6 (18-66; 7·7) years | RT-PCR, Virus isolation | Primary, Secondary | 1997 | 45 | 187 |
| Capeding 2010^33^ | Philippines | 10/2006-05/2009 | Median (95% CI), Symptomatic dengue patients: 7·2 (5·8-8·3) months | RT-PCR, HAI, PRNT50 | Primary | 1997 | 9 | 36 |
| Calvez 2020^34^ | Laos | 2012-2019 | Mean $\pm$ SD, Dengue positive: 23·9 $\pm$ 14·4 years, DENV-4: 27·4 $\pm$ 14·6 years | RT-PCR | Unknown | 1997 | 22 | 1176 |
| Mammen 2008^35^ | Thailand | 06/2004-11/2004, 06/2005-11/2005 | Range, Cluster enrolees with dengue infection: 0-15 years | RT-PCR | Unknown | SEARO 1999 | 0 | 7 |
| Trravassos da Rosa 2000^36^ | Brazil | 01/1997-12/1997 | No summary statistic | Virus isolation, indirect immunofluorescence test | Unknown | 1997 | 0 | 948 |
| Effler 2005^37^ | Hawaii | 09/2001-05/2002 | Median (Range), Persons testing positive for dengue: 41 (1-77) years | Virus isolation, RT-PCR | Unknown | 1997 | 0 | 15 |
| Salje 2018^38^ | Thailand | 1998-2003 | Mean (IQR) Age at recruitment: 9 (8-11) years | RT-PCR | Unknown | 1997 | 28 | 217 |
| Fakeeh 2001^39^ | Saudi Arabia | 02/1994-12/1999 | No summary statistic | Virus isolation, indirect immunofluorescent assay | Unknown | 1986 | 2 | 207 |
| Beckett 2005^40^ | Indonesia | 10/2001-10/2003 | Mean (Range): Post-enrolment dengue cases: 20·2 (5-47) years | RT-PCR, Virus isolation | Unknown | 1997 | 4 | 8 |
| Messer 2002^41^ | Sri Lanka | 1980-1985 | No summary statistics | Virus isolation, IFA, RT-PCR | Unknown | 1980* | 0 | 55 |
| Dung 2010^42^ | Vietnam | 10/2007-03/2008; 08/2006-03/2007 | Median (Range), DF: 11 (4-15) years, DHF: 13 (8-15) years | RT-PCR | Unknown | 1997 | 17 | 92 |
| Nainggolan 2023^43^ | Indonesia | 02/2010-01/2011 | Mean, Cases positive by RT-PCR: 24.3 | RT-PCR | Unknown | 1997 | 18 | 45 |
| Jadeja 2022^44^ | India | 07/2021-09/2021 | No summary statistic | RT-PCR | Unknown | SEARO 2011 | 47 | 101 |
| Narvaez 2025^45^ | Nicaragua | 2004-2024 | Mean age at disease onset for PDCS: 9.88, PDHS: 9.19 | RT-PCR | Primary, Secondary | 1997 | 365 | 1752 |

**Table S3:** Studies included for 1997-type severity – *Studies mention that they are using WHO guidelines but do not cite a specific document, and we thus assume a version based on the study period. **Study cites the WHO 2009 guidelines but uses DF/DHF/DSS classification. As these studies still make use of standard WHO guidelines, we include them in the review. HAI – haemagglutination-inhibition, PRNT – plaque reduction neutralisation test, RT-PCR – reverse transcription polymerase chain reaction

| **Study** | **Location** | **Study Period** | **Age (Statistic, Population: Value)** | **Serotyping Method** | **Prior Exposure Status** | **Severity Class Guideline** | **Severe** | **N** |
| --- | --- | --- | --- | --- | --- | --- | --- | --- |
| Nwe 2022^46^ | Myanmar | 06/2017-12/2019 | No summary statistic | RT-PCR | Unknown | 2009 | 18 | 83 |
| Azevedo 2019^47^ | Brazil | NA | Mean $\pm$ SD, DwoWS: 21 $\pm$ 16 years, DwWS: 28 $\pm$ 23 years, Severe dengue: 39 $\pm$ 36 years | RT-PCR, Virus Isolation | Primary, Secondary | 2009 | 25 | 299 |
| Sharp 2013^7^ | Puerto Rico | 01/2010-12/2010 | Median (Range), Lab positive dengue cases: 18 (0·08-102) years | RT-PCR | Unknown | 2009 | 739 | 7428 |
| Huang 2020^48^ | Taiwan | 07/2015-11/2015 | Median (IQR), Severe dengue: 75 (68-79) years, Mild dengue: 55 (35-71) years | RT-PCR | Primary, Secondary | 2009 | 138 | 798 |
| Martins 2014^49^ | Brazil | 01/2011-05/2011 | Mean $\pm$ SD (Range), dengue infected patients: 35·7 $\pm$ 15·7 (4-83) years | RT-PCR | Unknown | 2012 | 39 | 247 |
| Dussart 2012a^50^ | Brazil; French Guiana | 07/2006-06/2007 | Age for inclusion criteria: > 24 months | RT-PCR, Virus Isolation | Primary, Secondary | 2009 | 0 | 68 |
| Dussart 2012b^50^ | Cambodia; Vietnam | 07/2006-06/2007 | Age for inclusion criteria: > 24 months | RT-PCR, Virus Isolation | Primary, Secondary | 2009 | 21 | 84 |
| Mukherjee 2022^51^ | India | 07/2016-06/2017 | Mean $\pm$ SD, Dengue patients: 25·08 $\pm$ 12·70 years | RT-PCR | Unknown, Primary | 2009 | 6 | 29 |
| Racherla 2018^52^ | India | 10/2017-11/2017 | Mean $\pm$ SD, Adult patients: 35·5 $\pm$ 11·6 years, paediatric patients: 8·8 $\pm$ 5·2 years, geriatric patients: 68·6 $\pm$ 6·8 years | RT-PCR | Unknown | 2009 | 3 | 60 |
| Yung 2015^19^ | Singapore | 05/2005-12/2011 | Median (Range), DENV-1: 35 (19-74) years, DENV-2: 37 (18-77) years, DENV-3: 39 (19-87) years | RT-PCR | Unknown | 2009 | 30 | 451 |
| Ly 2022^53^ | Belize | 01/2020-12/2020 | Median (IQR), DENV positive patients: 21 (22) years | RT-PCR | Unknown | 2009 | 0 | 26 |
| Ta 2019^54^ | Vietnam | 06/2012-12/2014 | Mean $\pm$ SD, Severe dengue: 8 $\pm$ 2·6, Non-severe dengue: 8·2 $\pm$ 3·2 | RT-PCR | Unknown | 2009 | 8 | 61 |
| Mehta 2018^55^ | India | 01/2015-12/2015 | No summary statistic | RT-PCR | Unknown | 2009 | 10 | 115 |
| Rouers 2021^56^ | Singapore | NA | Mean $\pm$ SD, Patient cohort: 37·3 $\pm$ 11·7 years | RT-PCR | Primary, Secondary | 2009 | 2 | 44 |
| Guerra-Gomes 2017^57^ | Brazil | 2007-2015 | Mean, All dengue patients: 30·73, DENV-1: 29·85, DENV-2: 36, DENV-3: 29·5, DENV-4: 30·15 | RT-PCR | Unknown | 2009 | 1 | 15 |
| Braga 2016^58^ | Brazil | 05/2011-06/2014 | Range, Study criteria: 0-24 months | RT-PCR | Unknown | 2012 | 0 | 11 |
| Gupta 2015^59^ | Nepal | 07/2013-12/2013 | Mean $\pm$ SD, Patient analysed: 45·75 $\pm$ 38·61 years | RT-PCR | Unknown | 2009 | 0 | 15 |
| Perdomo-Celis 2017^60^ | Colombia | 07/2013-08/2015 | Median (Range), DwoWS: 36 (12-168) months, DwWS: 72 (9-168) months, Severe dengue: 45·5 (4-144) months | RT-PCR | Unknown | 2009 | 14 | 44 |
| Falconar 2012^29^ | Colombia | 2007-2007 | Mean (Range), Confirmed dengue infections: 8·1 (1-27) years | Virus isolation, IFA | Secondary | 2009 | 5 | 14 |
| Nguyen 2017^61^ | Vietnam | 10/2010-12/2013 | Median (IQR), Severe dengue: 9 (7-11), Non-severe dengue: 9 (6-11) | RT-PCR | Unknown | 2009 | 115 | 1944 |
| Estofolete 2023^62^ | Brazil | 12/2018-11/2019 | Mean, DV-ZV-: 38·2, DV-ZV+: 48, DV+ZV-: 40.17 | RT-PCR | Unknown | 2009 | 7 | 507 |
| Fiora 2024^63^ | Argentina | 01/2020-05/2020 | Median (IQR) all patients: 108 (84-144) Months | RT-PCR | Unknown | 2009 | 0 | 25 |
| Narvaez 2025^45^ | Nicaragua | 2004-2024 | Mean age at disease onset for PDCS: 9·88, PDHS: 9·19 | RT-PCR | Primary, Secondary | 2009 | 375 | 1840 |
| Odio 2025^64^ | Philippines | 05/2017-06/2017 | Mean (SD) for Symptomatic dengue: 10·91 (1.15) | RT-PCR | Secondary | 2009 | 0 | 42 |
| Burgos 2025^65^ | Peru | 10/2021-09/2023 | Mean (SD): Total: 29·18 (17·78), DENV-1: 28·33 (18·01), DENV-2: 29·92 (17·57) | RT-PCR | Unknown | 2009 | 2 | 420 |

**Table S4:** Studies included for 2009-type severity: HAI – haemagglutination-inhibition, PRNT – plaque reduction neutralisation test, RT-PCR – reverse transcription polymerase chain reaction

| **Study** | **Location** | **Study Period** | **Age (Statistic, Population: Value)** | **Serotyping Method** | **Prior Exposure Status** | **Severity Class Guideline** | **Severe** | **N** |
| --- | --- | --- | --- | --- | --- | --- | --- | --- |
| Burattini 2016^6^ | Brazil | 2000-2014 | No summary statistic | Not Mentioned | Unknown | Hospitalisation | 1597 | 12 323 |
| Hanna 2001^66^ | Australia | 1997-1999 | Median (Range), Dengue patients with known age: 36·6 (1·3-86·4) years | Virus Isolation, HAI, RT-PCR | Unknown | Hospitalisation | 101 | 498 |
| Santiago 2019^67^ | Puerto Rico | 2005-2006, 2012-2016? | Median (Range), DENV-2: 25 (0·5-81) years, DENV-3: 24 (0-84) years | RT-PCR | Unknown | Hospitalisation | 272 | 572 |
| Sabchareon 2012^18^ | Thailand | 2006-2009 | Mean (Range, Median, Mode), Serologically confirmed cases: 9·4 (4-14, 10, 11) years | RT-PCR | Unknown | Hospitalisation | 163 | 333 |
| Yung 2015^19^ | Singapore | 05/2005-12/2011 | Median (Range), DENV-1: 35 (19-74) years, DENV-2: 37 (18-77) years, DENV-3: 39 (19-87) years | RT-PCR | Unknown | Hospitalisation | 215 | 451 |
| Aubry 2019^68^ | French Polynesia | 02/2019-06/2019 | No summary statistic | RT-PCR | Unknown | Hospitalisation | 21 | 331 |
| Ly 2022^53^ | Belize | 01/2020-12/2020 | Median (IQR), DENV positive patients: 21 (22) years | RT-PCR | Unknown | Hospitalisation | 2 | 26 |
| Rouers 2021^56^ | Singapore | NA | Mean $\pm$ SD, Patient cohort: 37·3 $\pm$ 11·7 years | RT-PCR | Primary, Secondary | Hospitalisation | 12 | 44 |
| Duyen 2011^69^ | Vietnam | 06/2006-03/2008 | Median (IQR), Study participants: 11 (9-13) years | RT-PCR | Primary, Secondary | Hospitalisation | 5 | 167 |
| Capeding 2010^33^ | Philippines | 10/2006-05/2009 | Median (95% CI), Symptomatic dengue patients: 7·2 (5·8-8·3) months | RT-PCR, HAI, PRNT50 | Primary | Hospitalisation | 22 | 60 |
| Mammen 2008^35^ | Thailand | 06/2004-11/2004, 06/2005-11/2005 | Range, Cluster enrolees with dengue infection: 0-15 years | RT-PCR | Unknown | Hospitalisation | 1 | 9 |
| GomezDantes 1988^70^ | Mexico | NA | No summary statistic | Virus isolation, indirect immunofluorescent test | Unknown | Hospitalisation | 0 | 44 |
| Salje 2018^38^ | Thailand | NA | Mean (IQR) Age at recruitment: 9 (8-11) years | RT-PCR | Unknown | Hospitalisation | 50 | 217 |
| Hanna 1998^71^ | Australia | 1996-1997 | Range, Dengue patients in Cairns: 20-46 years | RT-PCR, Virus isolation, HAI | Unknown | Hospitalisation | 0 | 6 |
| AlBalushi 2023^72^ | Oman | 02/2022-04/2022 | Range, study population: 4-82 years | RT-PCR | Unknown | Hospitalisation | 93 | 169 |
| Estofolete 2023^62^ | Brazil | 12/2018-11/2019 | Mean, DV-ZV-: 38.2, DV-ZV+: 48, DV+ZV-: 40·17 | RT-PCR | Unknown | Hospitalisation | 62 | 496 |
| Fiora 2024^63^ | Argentina | 01/2020-05/2020 | Median (IQR), all patients: 108 (84-144) Months | RT-PCR | Unknown | Hospitalisation | 0 | 25 |
| Rahim 2023^73^ | Bangladesh | 2018-2022 | Mean Age, 2018 (DENV1: 20, DENV2: 31, DENV3: 23), 2019 (DENV1: 13, DENV3: 17), 2021 (DENV3: 30), 2022 (DENV3: 30) | RT-PCR | Unknown | Hospitalisation | 188 | 495 |
| Rodriguez 2024^74^ | Puerto Rico | 2010-2024 | Median (Range), all patients: 20 (0-102) | Unclear | Unknown | Hospitalisation | 10 659 | 27 048 |
| Burgos 2025^65^ | Peru | 10/2021-09/2023 | Mean (SD): Total: 29·18 (17·78), DENV-1: 28·33 (18·01), DENV-2: 29·92 (17·57) | RT-PCR | Unknown | Hospitalisation | 11 | 420 |

**Table S5:** Studies included for hospitalisation. HAI – haemagglutination-inhibition, PRNT – plaque reduction neutralisation test, RT-PCR – reverse transcription polymerase chain reaction

## Bias Assessment Results

| **Study** | **Q1** | **Q2** | **Q3** | **Q4** | **Q5** | **Q6** | **Q7** | **Q8** | **Q9** | **Q10** | **Total** | **Risk of Bias (Sum-based)** | **Risk of Bias (Subjective)** |
| --- | --- | --- | --- | --- | --- | --- | --- | --- | --- | --- | --- | --- | --- |
| Poeranto 2016 | 0 | 1 | 1 | 1 | 1 | 1 | 0 | 0 | 1 | 1 | 7 | Moderate Risk | Moderate Risk |
| Nwe 2022 | 1 | 1 | 1 | 1 | 1 | 1 | 1 | 1 | 1 | 1 | 10 | Low Risk | Low Risk |
| Osmali 2007 | 1 | 1 | 0 | 0 | 1 | 1 | 0 | 1 | 0 | 1 | 6 | Moderate Risk | Moderate Risk |
| Azevedo 2019 | 1 | 1 | 1 | 1 | 1 | 1 | 1 | 1 | 1 | 1 | 10 | Low Risk | Low Risk |
| Burattini 2016 | 1 | 1 | 1 | 1 | 0 | 1 | 1 | 0 | 1 | 1 | 8 | Low Risk | Low Risk |
| Hanna 2001 | 1 | 1 | 0 | 0 | 1 | 1 | 0 | 0 | 0 | 1 | 5 | Moderate Risk | Moderate Risk |
| Sharp 2013 | 1 | 1 | 1 | 1 | 1 | 1 | 1 | 1 | 1 | 0 | 9 | Low Risk | Moderate Risk |
| Huang 2020 | 1 | 1 | 0 | 0 | 1 | 1 | 1 | 1 | 0 | 1 | 7 | Moderate Risk | Moderate Risk |
| Myint 2006 | 0 | 1 | 1 | 1 | 1 | 1 | 1 | 1 | 1 | 1 | 9 | Low Risk | Low Risk |
| Kallol 2016 | 1 | 1 | 1 | 1 | 1 | 1 | 1 | 1 | 1 | 1 | 10 | Low Risk | Low Risk |
| Dewi 2014 | 0 | 1 | 1 | 1 | 1 | 1 | 1 | 0 | 1 | 1 | 8 | Low Risk | Low Risk |
| Montoya 2003 | 1 | 1 | 0 | 0 | 1 | 1 | 1 | 1 | 0 | 1 | 7 | Moderate Risk | Moderate Risk |
| Dewi 2021 | 0 | 1 | 1 | 1 | 1 | 1 | 1 | 1 | 1 | 1 | 9 | Low Risk | Low Risk |
| Santiago 2019 | 1 | 1 | 0 | 0 | 1 | 1 | 1 | 1 | 1 | 1 | 8 | Low Risk | Low Risk |
| Whitehorn 2017 | 1 | 0 | 0 | 0 | 1 | 1 | 1 | 0 | 0 | 1 | 5 | Moderate Risk | Moderate Risk |
| Wardhani 2017 | 1 | 1 | 1 | 1 | 1 | 1 | 1 | 1 | 1 | 1 | 10 | Low Risk | Low Risk |
| Martins 2014 | 1 | 1 | 1 | 1 | 1 | 1 | 1 | 1 | 1 | 1 | 10 | Low Risk | Low Risk |
| Dussart 2012a  (Brazil, French Guiana) | 1 | 1 | 1 | 1 | 1 | 1 | 0 | 0 | 1 | 1 | 8 | Low Risk | Moderate Risk |
| Dussart 2012b (Cambodia, Vietnam) | 1 | 1 | 1 | 1 | 1 | 1 | 0 | 0 | 1 | 1 | 8 | Low Risk | Moderate Risk |
| Harris 2000 | 1 | 1 | 1 | 1 | 1 | 1 | 1 | 1 | 1 | 1 | 10 | Low Risk | Low Risk |
| Mukherjee 2022 | 1 | 1 | 0 | 0 | 1 | 1 | 1 | 0 | 1 | 1 | 7 | Moderate Risk | Moderate Risk |
| Racherla 2018 | 1 | 1 | 1 | 1 | 1 | 1 | 1 | 1 | 1 | 1 | 10 | Low Risk | Low Risk |
| Kerdpanich 2021 | 1 | 1 | 1 | 1 | 1 | 1 | 1 | 0 | 1 | 1 | 9 | Low Risk | Low Risk |
| Guilarde 2008 | 0 | 1 | 1 | 1 | 1 | 1 | 1 | 1 | 1 | 1 | 9 | Low Risk | Low Risk |
| WHO 1987 | 1 | 0 | 0 | 0 | 1 | 1 | 1 | 1 | 0 | 1 | 6 | Moderate Risk | Moderate Risk |
| Sabchareon 2012 | 0 | 1 | 1 | 1 | 1 | 1 | 1 | 0 | 1 | 1 | 8 | Low Risk | Low Risk |
| Yung 2015 | 0 | 1 | 1 | 1 | 1 | 1 | 1 | 1 | 1 | 1 | 9 | Low Risk | Low Risk |
| Aubry 2019 | 1 | 1 | 0 | 0 | 1 | 1 | 1 | 0 | 0 | 1 | 6 | Moderate Risk | Moderate Risk |
| Dumre 2017 | 1 | 1 | 1 | 0 | 1 | 1 | 1 | 1 | 1 | 1 | 9 | Low Risk | Low Risk |
| Ayolabi 2019 | 1 | 1 | 1 | 1 | 1 | 1 | 1 | 1 | 0 | 1 | 9 | Low Risk | Low Risk |
| Chakravarti 2010 | 1 | 1 | 1 | 1 | 1 | 1 | 1 | 1 | 1 | 1 | 10 | Low Risk | Low Risk |
| Ly 2022 | 1 | 1 | 1 | 1 | 1 | 1 | 1 | 1 | 1 | 1 | 10 | Low Risk | Low Risk |
| Rao 2018 | 1 | 1 | 1 | 1 | 1 | 1 | 1 | 1 | 1 | 1 | 10 | Low Risk | Low Risk |
| TramVan 2019 | 0 | 1 | 1 | 1 | 1 | 1 | 1 | 1 | 1 | 1 | 9 | Low Risk | Low Risk |
| Stephens 2002 | 0 | 1 | 1 | 1 | 1 | 1 | 1 | 1 | 1 | 1 | 9 | Low Risk | Low Risk |
| Mehta 2018 | 1 | 0 | 1 | 1 | 1 | 1 | 1 | 1 | 0 | 1 | 8 | Low Risk | Moderate Risk |
| Rouers 2021 | 1 | 1 | 1 | 1 | 1 | 1 | 1 | 1 | 1 | 1 | 10 | Low Risk | Low Risk |
| Reyes 2010 | 1 | 1 | 1 | 1 | 1 | 1 | 1 | 1 | 1 | 1 | 10 | Low Risk | Low Risk |
| Thomas 2008 | 1 | 0 | 1 | 1 | 1 | 1 | 1 | 1 | 0 | 1 | 8 | Low Risk | Low Risk |
| Duyen 2011 | 1 | 0 | 1 | 1 | 1 | 1 | 1 | 1 | 1 | 1 | 9 | Low Risk | Low Risk |
| Feres 2006 | 1 | 1 | 0 | 0 | 0 | 1 | 1 | 1 | 0 | 1 | 6 | Moderate Risk | Moderate Risk |
| Guerra-Gomes 2017 | 1 | 1 | 0 | 0 | 0 | 1 | 1 | 1 | 0 | 1 | 6 | Moderate Risk | Moderate Risk |
| Sharma 2020 | 1 | 1 | 1 | 0 | 1 | 1 | 1 | 1 | 0 | 1 | 8 | Low Risk | Low Risk |
| Braga 2016 | 0 | 1 | 1 | 1 | 1 | 1 | 1 | 1 | 1 | 1 | 9 | Low Risk | Moderate Risk |
| Gupta 2015 | 1 | 1 | 0 | 0 | 1 | 1 | 0 | 1 | 1 | 1 | 7 | Moderate Risk | Moderate Risk |
| Thomas 2010 | 1 | 1 | 0 | 1 | 1 | 1 | 1 | 1 | 1 | 1 | 9 | Low Risk | Low Risk |
| Perdomo-Celis 2017 | 0 | 1 | 1 | 1 | 1 | 1 | 1 | 1 | 1 | 1 | 9 | Low Risk | Low Risk |
| Fried 2010 | 0 | 1 | 1 | 1 | 1 | 1 | 1 | 1 | 1 | 1 | 9 | Low Risk | Low Risk |
| Falconar 2012 | 1 | 1 | 1 | 1 | 1 | 1 | 1 | 0 | 1 | 1 | 9 | Low Risk | Moderate Risk |
| Ocazionez 2006 | 1 | 1 | 0 | 0 | 1 | 1 | 1 | 0 | 1 | 1 | 7 | Moderate Risk | Moderate Risk |
| Kao 2016 | 1 | 1 | 0 | 0 | 1 | 1 | 1 | 0 | 1 | 1 | 7 | Moderate Risk | Moderate Risk |
| Kosasih 2016 | 1 | 1 | 1 | 1 | 1 | 1 | 1 | 1 | 1 | 1 | 10 | Low Risk | Low Risk |
| Capeding 2010 | 0 | 1 | 1 | 1 | 1 | 1 | 1 | 1 | 1 | 1 | 9 | Low Risk | Low Risk |
| Calvez 2020 | 1 | 1 | 0 | 0 | 1 | 1 | 1 | 0 | 1 | 1 | 7 | Moderate Risk | Moderate Risk |
| Mammen 2008 | 1 | 0 | 0 | 0 | 1 | 1 | 1 | 1 | 1 | 1 | 7 | Moderate Risk | Moderate Risk |
| Rosa 2000 | 1 | 1 | 1 | 1 | 1 | 1 | 1 | 1 | 1 | 1 | 10 | Low Risk | Low Risk |
| GomezDantes 1988 | 1 | 1 | 1 | 1 | 1 | 1 | 1 | 1 | 0 | 1 | 9 | Low Risk | Low Risk |
| Effler 2005 | 1 | 1 | 1 | 1 | 0 | 1 | 1 | 1 | 0 | 1 | 8 | Low Risk | Low Risk |
| Salje 2018 | 1 | 1 | 0 | 1 | 1 | 1 | 1 | 1 | 1 | 1 | 9 | Low Risk | Low Risk |
| Hanna 1998 | 1 | 1 | 1 | 1 | 1 | 1 | 0 | 0 | 0 | 1 | 7 | Moderate Risk | Moderate Risk |
| Fakeeh 2001 | 1 | 1 | 1 | 1 | 1 | 1 | 1 | 1 | 0 | 1 | 9 | Low Risk | Low Risk |
| Beckett 2005 | 1 | 1 | 1 | 0 | 1 | 1 | 1 | 1 | 1 | 1 | 9 | Low Risk | Low Risk |
| Messer 2002 | 1 | 1 | 1 | 1 | 1 | 1 | 1 | 0 | 0 | 1 | 8 | Low Risk | Low Risk |
| Dung 2010 | 0 | 1 | 1 | 1 | 1 | 1 | 1 | 1 | 1 | 1 | 9 | Low Risk | Low Risk |
| Nguyen 2017 | 1 | 0 | 1 | 1 | 1 | 1 | 1 | 0 | 1 | 1 | 8 | Low Risk | Low Risk |
| AlBalushi 2023 | 1 | 1 | 1 | 1 | 1 | 1 | 1 | 1 | 0 | 1 | 9 | Low Risk | Low Risk |
| Estofolete 2023 | 1 | 1 | 1 | 1 | 0 | 1 | 1 | 1 | 0 | 1 | 8 | Low Risk | Low Risk |
| Fiora 2024 | 0 | 1 | 1 | 1 | 0 | 1 | 1 | 1 | 1 | 1 | 8 | Low Risk | Low Risk |
| Nainggolan 2023 | 1 | 0 | 1 | 1 | 1 | 1 | 1 | 1 | 1 | 1 | 9 | Low Risk | Low Risk |
| Jadeja 2022 | 1 | 1 | 1 | 1 | 1 | 1 | 1 | 1 | 0 | 1 | 9 | Low Risk | Low Risk |
| Narvaez 2025 | 0 | 1 | 1 | 1 | 1 | 1 | 1 | 1 | 1 | 1 | 9 | Low Risk | Low Risk |
| Odio 2025 | 1 | 0 | 1 | 1 | 1 | 1 | 1 | 1 | 0 | 1 | 8 | Low Risk | Low Risk |
| Rahim 2023 | 1 | 1 | 1 | 1 | 1 | 1 | 1 | 1 | 1 | 1 | 10 | Low Risk | Low Risk |
| Rodriguez 2024 | 1 | 1 | 1 | 0 | 1 | 1 | 0 | 0 | 0 | 1 | 6 | Moderate Risk | Moderate Risk |
| Burgos 2025 | 1 | 1 | 1 | 1 | 1 | 1 | 1 | 1 | 0 | 1 | 9 | Low Risk | Low Risk |

Table S6: Breakdown of bias assessment results for Reviewer A (based on Table S2)

| **Study** | **Q1** | **Q2** | **Q3** | **Q4** | **Q5** | **Q6** | **Q7** | **Q8** | **Q9** | **Q10** | **Total** | **Risk of Bias (Sum-based)** | **Risk of Bias (Subjective)** |
| --- | --- | --- | --- | --- | --- | --- | --- | --- | --- | --- | --- | --- | --- |
| Poeranto 2016 | 1 | 1 | 1 | 1 | 0 | 1 | 1 | 1 | 1 | 1 | 9 | Low Risk | Low Risk |
| Nwe 2022 | 1 | 1 | 0 | 0 | 1 | 1 | 1 | 1 | 1 | 1 | 8 | Low Risk | Moderate Risk |
| Osmali 2007 | 0 | 0 | 0 | 1 | 1 | 1 | 1 | 1 | 1 | 1 | 7 | Moderate Risk | Moderate Risk |
| Azevedo 2019 | 1 | 1 | 1 | 1 | 1 | 1 | 1 | 1 | 1 | 1 | 10 | Low Risk | Low Risk |
| Burattini 2016 | 1 | 1 | 0 | 0 | 0 | 1 | 1 | 1 | 1 | 1 | 7 | Moderate Risk | Moderate Risk |
| Hanna 2001 | 1 | 1 | 1 | 0 | 1 | 1 | 1 | 1 | 0 | 1 | 8 | Low Risk | Moderate Risk |
| Sharp 2013 | 1 | 1 | 0 | 0 | 1 | 1 | 1 | 1 | 1 | 1 | 8 | Low Risk | Moderate Risk |
| Huang 2020 | 1 | 1 | 0 | 1 | 0 | 1 | 1 | 1 | 1 | 1 | 8 | Low Risk | Moderate Risk |
| Myint 2006 | 1 | 1 | 1 | 1 | 1 | 1 | 1 | 1 | 1 | 1 | 10 | Low Risk | Low Risk |
| Kallol 2016 | 1 | 1 | 1 | 0 | 1 | 1 | 1 | 1 | 1 | 1 | 9 | Low Risk | Low Risk |
| Dewi 2014 | 0 | 1 | 1 | 0 | 1 | 1 | 1 | 1 | 0 | 1 | 7 | Moderate Risk | Moderate Risk |
| Montoya 2003 | 1 | 0 | 1 | 1 | 1 | 1 | 1 | 1 | 1 | 1 | 9 | Low Risk | Low Risk |
| Dewi 2021 | 0 | 1 | 1 | 0 | 1 | 1 | 1 | 1 | 0 | 1 | 7 | Moderate Risk | Low Risk |
| Santiago 2019 | 1 | 1 | 1 | 0 | 1 | 1 | 1 | 1 | 0 | 1 | 8 | Low Risk | Moderate Risk |
| Whitehorn 2017 | 1 | 1 | 1 | 1 | 1 | 0 | 1 | 1 | 1 | 1 | 9 | Low Risk | Low Risk |
| Wardhani 2017 | 1 | 1 | 1 | 0 | 1 | 1 | 1 | 1 | 1 | 1 | 9 | Low Risk | Low Risk |
| Martins 2014 | 1 | 1 | 1 | 1 | 1 | 1 | 1 | 1 | 1 | 1 | 10 | Low Risk | Low Risk |
| Dussart 2012a (Brazil, French Guiana) | 1 | 1 | 1 | 0 | 1 | 1 | 0 | 1 | 1 | 1 | 8 | Low Risk | Low Risk |
| Dussart 2012b (Cambodia, Vietnam) | 1 | 1 | 1 | 0 | 1 | 1 | 0 | 1 | 1 | 1 | 8 | Low Risk | Low Risk |
| Harris 2000 | 1 | 1 | 1 | 0 | 1 | 1 | 1 | 1 | 1 | 1 | 9 | Low Risk | Low Risk |
| Mukherjee 2022 | 0 | 0 | 0 | 1 | 1 | 1 | 1 | 1 | 1 | 1 | 7 | Moderate Risk | Moderate Risk |
| Racherla 2018 | 1 | 1 | 1 | 0 | 1 | 1 | 1 | 1 | 1 | 1 | 9 | Low Risk | Low Risk |
| Kerdpanich 2021 | 0 | 1 | 1 | 0 | 0 | 1 | 1 | 1 | 1 | 1 | 7 | Moderate Risk | Moderate Risk |
| Guilarde 2008 | 0 | 1 | 0 | 1 | 1 | 1 | 1 | 1 | 1 | 1 | 8 | Low Risk | Moderate Risk |
| WHO 1987 | 1 | 1 | 1 | 0 | 0 | 1 | 0 | 1 | 1 | 1 | 7 | Moderate Risk | Moderate Risk |
| Sabchareon 2012 | 0 | 1 | 1 | 1 | 1 | 1 | 1 | 0 | 1 | 1 | 8 | Low Risk | Low Risk |
| Yung 2015 | 0 | 1 | 1 | 1 | 1 | 1 | 1 | 1 | 1 | 1 | 9 | Low Risk | Low Risk |
| Aubry 2019 | 1 | 1 | 1 | 1 | 1 | 1 | 1 | 1 | 1 | 1 | 10 | Low Risk | Low Risk |
| Dumre 2017 | 1 | 1 | 1 | 1 | 1 | 1 | 1 | 1 | 1 | 1 | 10 | Low Risk | Low Risk |
| Ayolabi 2019 | 0 | 0 | 1 | 0 | 1 | 1 | 1 | 1 | 1 | 1 | 7 | Moderate Risk | Moderate Risk |
| Chakravarti 2010 | 0 | 0 | 1 | 0 | 0 | 1 | 1 | 1 | 1 | 1 | 6 | Moderate Risk | Moderate Risk |
| Ly 2022 | 0 | 1 | 1 | 1 | 1 | 1 | 1 | 1 | 0 | 1 | 8 | Low Risk | Moderate Risk |
| Rao 2018 | 1 | 1 | 1 | 0 | 1 | 1 | 1 | 1 | 1 | 1 | 9 | Low Risk | Moderate Risk |
| TramVan 2019 | 0 | 1 | 1 | 0 | 1 | 1 | 1 | 1 | 1 | 1 | 8 | Low Risk | Moderate Risk |
| Stephens 2002 | 0 | 1 | 1 | 0 | 1 | 1 | 1 | 1 | 1 | 1 | 8 | Low Risk | Moderate Risk |
| Mehta 2018 | 0 | 0 | 1 | 1 | 0 | 1 | 1 | 1 | 1 | 1 | 7 | Moderate Risk | Moderate Risk |
| Rouers 2021 | 0 | 0 | 1 | 1 | 1 | 1 | 1 | 1 | 1 | 1 | 8 | Low Risk | Moderate Risk |
| Reyes 2010 | 1 | 1 | 1 | 1 | 1 | 1 | 1 | 1 | 1 | 1 | 10 | Low Risk | Low Risk |
| Thomas 2008 | 0 | 1 | 1 | 1 | 1 | 1 | 1 | 1 | 0 | 1 | 8 | Low Risk | Moderate Risk |
| Duyen 2011 | 0 | 1 | 1 | 1 | 1 | 1 | 1 | 1 | 1 | 1 | 9 | Low Risk | Low Risk |
| Feres 2006 | 1 | 1 | 1 | 1 | 0 | 1 | 1 | 1 | 0 | 1 | 8 | Low Risk | Low Risk |
| Guerra-Gomes 2017 | 1 | 1 | 1 | 0 | 0 | 1 | 1 | 1 | 1 | 1 | 8 | Low Risk | Low Risk |
| Sharma 2020 | 1 | 1 | 1 | 0 | 1 | 1 | 1 | 1 | 1 | 1 | 9 | Low Risk | Low Risk |
| Braga 2016 | 0 | 1 | 1 | 1 | 1 | 1 | 1 | 1 | 1 | 1 | 9 | Low Risk | Low Risk |
| Gupta 2015 | 1 | 1 | 1 | 1 | 1 | 1 | 1 | 1 | 1 | 1 | 10 | Low Risk | Low Risk |
| Thomas 2010 | 0 | 1 | 1 | 1 | 1 | 1 | 1 | 1 | 1 | 1 | 9 | Low Risk | Low Risk |
| Perdomo-Celis 2017 | 0 | 1 | 1 | 0 | 1 | 1 | 1 | 1 | 1 | 1 | 8 | Low Risk | Low Risk |
| Fried 2010 | 0 | 1 | 1 | 1 | 1 | 1 | 1 | 1 | 1 | 1 | 9 | Low Risk | Low Risk |
| Falconar 2012 | 1 | 1 | 1 | 0 | 1 | 1 | 1 | 1 | 1 | 1 | 9 | Low Risk | Low Risk |
| Ocazionez 2006 | 1 | 1 | 0 | 0 | 0 | 1 | 1 | 0 | 1 | 1 | 6 | Moderate Risk | Moderate Risk |
| Kao 2016 | 1 | 1 | 1 | 0 | 1 | 1 | 1 | 0 | 1 | 1 | 8 | Low Risk | Low Risk |
| Kosasih 2016 | 0 | 1 | 1 | 0 | 1 | 1 | 1 | 1 | 1 | 1 | 8 | Low Risk | Low Risk |
| Capeding 2010 | 0 | 1 | 1 | 1 | 1 | 1 | 1 | 1 | 1 | 1 | 9 | Low Risk | Low Risk |
| Calvez 2020 | 1 | 1 | 1 | 0 | 0 | 1 | 1 | 1 | 1 | 1 | 8 | Low Risk | Low Risk |
| Mammen 2008 | 0 | 1 | 1 | 1 | 1 | 1 | 1 | 1 | 1 | 1 | 9 | Low Risk | Low Risk |
| Rosa 2000 | 1 | 1 | 1 | 0 | 1 | 1 | 1 | 0 | 1 | 1 | 8 | Low Risk | Low Risk |
| GomezDantes 1988 | 1 | 1 | 1 | 0 | 1 | 1 | 1 | 0 | 1 | 1 | 8 | Low Risk | Moderate Risk |
| Effler 2005 | 1 | 1 | 1 | 0 | 0 | 1 | 1 | 0 | 1 | 1 | 7 | Moderate Risk | Low Risk |
| Salje 2018 | 0 | 1 | 1 | 1 | 1 | 1 | 1 | 1 | 1 | 1 | 9 | Low Risk | Low Risk |
| Hanna 1998 | 1 | 1 | 1 | 1 | 1 | 1 | 1 | 0 | 1 | 1 | 9 | Low Risk | Low Risk |
| Fakeeh 2001 | 1 | 1 | 1 | 0 | 1 | 1 | 1 | 0 | 1 | 1 | 8 | Low Risk | Low Risk |
| Beckett 2005 | 1 | 1 | 1 | 1 | 1 | 1 | 1 | 0 | 1 | 1 | 9 | Low Risk | Low Risk |
| Messer 2002 | 0 | 1 | 1 | 1 | 1 | 1 | 1 | 0 | 1 | 1 | 8 | Low Risk | Low Risk |
| Dung 2010 | 0 | 1 | 1 | 1 | 1 | 1 | 1 | 1 | 1 | 1 | 9 | Low Risk | Low Risk |
| Nguyen 2017 | 1 | 1 | 1 | 1 | 1 | 1 | 1 | 1 | 1 | 1 | 10 | Low Risk | Low Risk |
| AlBalushi 2023 | 1 | 1 | 1 | 1 | 0 | 1 | 1 | 1 | 1 | 1 | 9 | Low Risk | Low Risk |
| Estofolete 2023 | 1 | 1 | 1 | 1 | 0 | 1 | 1 | 1 | 1 | 1 | 9 | Low Risk | Low Risk |
| Fiora 2024 | 0 | 1 | 1 | 0 | 1 | 1 | 1 | 1 | 1 | 1 | 8 | Low Risk | Low Risk |
| Nainggolan 2023 | 0 | 1 | 1 | 1 | 1 | 1 | 1 | 1 | 1 | 1 | 9 | Low Risk | Low Risk |
| Jadeja 2022 | 1 | 1 | 1 | 1 | 1 | 1 | 1 | 1 | 1 | 1 | 10 | Low Risk | Low Risk |
| Narvaez 2025 | 1 | 1 | 1 | 1 | 1 | 1 | 1 | 1 | 1 | 1 | 10 | Low Risk | Low Risk |
| Odio 2025 | 0 | 1 | 1 | 1 | 1 | 1 | 1 | 1 | 1 | 1 | 9 | Low Risk | Low Risk |
| Rahim 2023 | 1 | 1 | 1 | 1 | 1 | 1 | 1 | 1 | 1 | 1 | 10 | Low Risk | Low Risk |
| Rodriguez 2024 | 1 | 1 | 1 | 0 | 0 | 1 | 1 | 1 | 1 | 1 | 8 | Low Risk | Low Risk |
| Burgos 2025 | 1 | 1 | 0 | 0 | 0 | 1 | 1 | 1 | 1 | 1 | 7 | Moderate Risk | Moderate Risk |

Table S7: Breakdown of bias assessment results for Reviewer B (based on Table S2)

## Assessing Small Study Effects

To assess small study effects, we sought to answer the question “do small studies systematically report higher or lower proportions of severe dengue” for each severity definition. We fitted a linear regression model with outcome-specific random effect $\epsilon_{ijk\mathcal{l}}$ as dependent variable and inverse sample size $\frac{1}{n_{ijk\mathcal{l}}}$ as the independent variable (shown in the equation below, where $E\left[ . \right]$ refers to the expected value) – a modified version of Peters’ regression adapted for our Bayesian meta-analysis.^75^

$$E\left[ \frac{\epsilon_{ijk\mathcal{l}}}{\sigma_{jk\mathcal{l}}} \right]=b+m\left( \frac{1}{n_{ijk\mathcal{l}}} \right)$$

Eq. S10

We fitted this regression model for each posterior sample of $\epsilon_{ijk\mathcal{l}}$ and ($\sigma_{jk\mathcal{l}})$. If the slope $m$ has a credible interval that is completely above or below 0, then there is a statistically significant small study effect. The results of this regression are shown in Table S8 below. Since the slope of the regression model crosses zero across all three severity definitions, then there was not enough evidence to conclude the presence of small study effects.

| **Severity Definition** | $\boldsymbol{m (95\% CrI)}$ |
| --- | --- |
| 1997-type | 2·78 (95% CrI: -0·03 to 5·63) |
| 2009-type | -1·67 (95% CrI: -5·41 to 1·92) |
| Hospitalisation | -4·03 (95% CrI: -8·82 to 0·79) |

Table S8: Slope $m$ of regression for small study effects for each severity definition

As a sensitivity analysis, we also ran standard Peters’ regression for each of the three severity definitions using the metaprop and metabias methods from the meta (v8.5.0) package in R with resulting $p$-values presented in Table S9 below.

| **Severity Definition** | $\boldsymbol{p}$**-value Peters’ Regression** |
| --- | --- |
| 1997-type | 0·0851 |
| 2009-type | 0·0622 |
| Hospitalisation | 0·8391 |

Table S9: $p$-value of Peters’ regression for each severity definition

Since $p$>0.05 across all three severity definitions, the results of the standard analysis are consistent with our adapted version: there is not enough evidence to conclude the presence of small study effects.

## Trace Plots and Parameter Estimates (1997-type Severity)

Figure S2: Trace plot for random effects $\epsilon_{ijk\mathcal{l}}$ for 1997-type severity

Figure S3: Trace plot for prior exposure-serotype effects $\beta_{k\mathcal{l}}$, region effect $\beta_{j}$, intercept $\alpha$, and standard deviation of random effects $\sigma_{jk\mathcal{l}}$ for 1997-type severity.

Figure S4: Trace plot for region-specific correlation matrices $P_{j}$ for 1997-type severity (including diagonal elements that are always 1 by definition)

| Parameter | Mean | 2.50% | 97.50% | SD | SE_Mean | ESS | Rhat |
| --- | --- | --- | --- | --- | --- | --- | --- |
| beta_sero_prior[1] | -1.5372 | -2.8259 | -0.2647 | 0.6521 | 0.0089 | 5375.0613 | 1.0009 |
| beta_sero_prior[2] | -1.0144 | -2.0624 | 0.0996 | 0.5493 | 0.0071 | 5959.7409 | 1.0004 |
| beta_sero_prior[3] | -1.0129 | -2.0284 | 0.0927 | 0.5404 | 0.0070 | 6002.3408 | 1.0002 |
| beta_sero_prior[4] | -1.6430 | -2.9979 | -0.2850 | 0.6734 | 0.0077 | 7666.4091 | 1.0006 |
| beta_sero_prior[5] | -1.6986 | -3.5761 | 0.1389 | 0.9192 | 0.0087 | 11289.9489 | 1.0000 |
| beta_sero_prior[6] | -1.9438 | -4.3225 | -0.0198 | 1.1182 | 0.0119 | 8875.7382 | 1.0003 |
| beta_sero_prior[7] | -0.3560 | -1.2876 | 0.6826 | 0.4991 | 0.0064 | 6028.2986 | 1.0006 |
| beta_sero_prior[8] | 0.1078 | -1.1119 | 1.4295 | 0.6361 | 0.0069 | 8463.9432 | 1.0003 |
| beta_sero_prior[9] | 0.4718 | -0.4688 | 1.5155 | 0.5032 | 0.0064 | 6127.7843 | 1.0002 |
| beta_sero_prior[10] | -0.1170 | -1.3869 | 1.1843 | 0.6424 | 0.0068 | 9037.5217 | 1.0001 |
| beta_reg | -0.6781 | -1.3085 | -0.0919 | 0.3039 | 0.0031 | 9358.3950 | 0.9999 |
| intercept | -0.9896 | -1.9356 | -0.1667 | 0.4466 | 0.0061 | 5412.4623 | 1.0003 |
| sigma[1] | 2.5777 | 1.3074 | 4.5677 | 0.8403 | 0.0078 | 11573.6862 | 1.0004 |
| sigma[2] | 1.3879 | 0.6420 | 2.6451 | 0.5174 | 0.0048 | 11451.6725 | 1.0002 |
| sigma[3] | 1.0464 | 0.3511 | 2.4375 | 0.5486 | 0.0050 | 12207.8016 | 1.0001 |
| sigma[4] | 1.3674 | 0.5509 | 3.0025 | 0.6389 | 0.0054 | 14007.7134 | 1.0000 |
| sigma[5] | 1.5667 | 0.0665 | 4.2519 | 1.1222 | 0.0103 | 11980.8321 | 1.0001 |
| sigma[6] | 0.7492 | 0.0251 | 2.7528 | 0.7275 | 0.0057 | 16310.8088 | 0.9999 |
| sigma[7] | 1.7805 | 0.4598 | 3.9276 | 0.8898 | 0.0085 | 11022.3208 | 1.0006 |
| sigma[8] | 0.7680 | 0.0217 | 2.8759 | 0.7615 | 0.0057 | 18025.3338 | 0.9999 |
| sigma[9] | 2.1090 | 1.3302 | 3.2866 | 0.5085 | 0.0061 | 6852.1532 | 1.0003 |
| sigma[10] | 2.0211 | 1.3374 | 3.0397 | 0.4380 | 0.0044 | 10016.4828 | 1.0000 |
| sigma[11] | 1.6120 | 1.0019 | 2.5515 | 0.4019 | 0.0039 | 10698.4703 | 1.0004 |
| sigma[12] | 2.2998 | 1.1145 | 4.3806 | 0.8517 | 0.0067 | 16044.4014 | 1.0004 |
| sigma[13] | 1.7205 | 0.2837 | 4.0405 | 0.9680 | 0.0085 | 12909.3671 | 1.0002 |
| sigma[14] | 2.1968 | 0.1178 | 5.4173 | 1.4341 | 0.0136 | 11181.3971 | 1.0003 |
| sigma[15] | 0.4054 | 0.0143 | 1.3850 | 0.3755 | 0.0032 | 13669.6242 | 1.0002 |
| sigma[16] | 1.0462 | 0.4169 | 2.3934 | 0.5140 | 0.0043 | 14291.4549 | 1.0000 |
| sigma[17] | 1.3404 | 0.6287 | 2.7327 | 0.5459 | 0.0048 | 13025.3890 | 1.0000 |
| sigma[18] | 0.7558 | 0.2773 | 1.6979 | 0.3722 | 0.0029 | 16226.3280 | 1.0000 |
| sigma[19] | 1.0502 | 0.1995 | 2.4925 | 0.5843 | 0.0052 | 12748.9147 | 1.0003 |
| eps_vals[1] | -0.8926 | -1.9355 | 0.2251 | 0.5491 | 0.0077 | 5110.3689 | 1.0011 |
| eps_vals[2] | 0.0573 | -0.7235 | 0.8390 | 0.3934 | 0.0042 | 8681.1201 | 1.0008 |
| eps_vals[3] | -0.6684 | -1.4163 | 0.0445 | 0.3683 | 0.0035 | 10939.0190 | 1.0004 |
| eps_vals[4] | -0.6792 | -1.7278 | 0.4645 | 0.5444 | 0.0046 | 13927.0033 | 1.0002 |
| eps_vals[5] | -4.7373 | -9.0652 | -2.1235 | 1.8077 | 0.0161 | 12649.1108 | 1.0005 |
| eps_vals[6] | -2.3353 | -5.0828 | -0.5568 | 1.1692 | 0.0088 | 17624.1750 | 1.0002 |
| eps_vals[7] | -1.8316 | -3.3326 | -0.4157 | 0.7423 | 0.0085 | 7602.7610 | 1.0006 |
| eps_vals[8] | -0.5402 | -2.0796 | 0.7630 | 0.7197 | 0.0056 | 16576.3289 | 1.0005 |
| eps_vals[9] | -0.9406 | -2.6506 | 0.2123 | 0.7422 | 0.0055 | 18261.4197 | 1.0002 |
| eps_vals[10] | 0.6654 | -0.2909 | 1.6420 | 0.4872 | 0.0041 | 14078.2812 | 1.0000 |
| eps_vals[11] | 2.3578 | 0.7682 | 3.9405 | 0.8077 | 0.0084 | 9311.2491 | 1.0007 |
| eps_vals[12] | -0.4857 | -3.5862 | 1.5699 | 1.2933 | 0.0087 | 21928.2284 | 1.0000 |
| eps_vals[13] | 0.6814 | -1.4552 | 3.5779 | 1.2546 | 0.0115 | 11827.9606 | 1.0008 |
| eps_vals[14] | -0.1130 | -1.0478 | 0.7208 | 0.4156 | 0.0033 | 16074.1764 | 1.0000 |
| eps_vals[15] | 1.8840 | 0.4144 | 3.3656 | 0.7498 | 0.0090 | 6946.5951 | 1.0006 |
| eps_vals[16] | -0.2072 | -1.6125 | 0.7765 | 0.5672 | 0.0043 | 17475.7523 | 0.9999 |
| eps_vals[17] | 0.9064 | -0.4353 | 2.4319 | 0.7336 | 0.0078 | 8777.7487 | 1.0002 |
| eps_vals[18] | -1.8751 | -6.7114 | 1.1782 | 2.0090 | 0.0143 | 19861.8845 | 1.0001 |
| eps_vals[19] | -3.1141 | -6.4513 | -0.8450 | 1.4308 | 0.0103 | 19186.1478 | 1.0000 |
| eps_vals[20] | -2.9956 | -6.2169 | -0.7825 | 1.3779 | 0.0097 | 20376.8483 | 1.0002 |
| eps_vals[21] | 1.8955 | 1.0445 | 2.7781 | 0.4406 | 0.0047 | 8872.7991 | 1.0005 |
| eps_vals[22] | 0.8492 | -0.8519 | 2.4480 | 0.8366 | 0.0053 | 25086.0101 | 1.0000 |
| eps_vals[23] | -2.9439 | -5.9864 | -0.8496 | 1.3211 | 0.0101 | 17113.1185 | 1.0001 |
| eps_vals[24] | -1.9538 | -2.9799 | -0.9578 | 0.5167 | 0.0050 | 10649.5098 | 1.0003 |
| eps_vals[25] | -2.0190 | -3.3214 | -0.8986 | 0.6111 | 0.0048 | 16491.5316 | 0.9999 |
| eps_vals[26] | 2.7961 | 1.4762 | 4.2263 | 0.7029 | 0.0080 | 7709.9644 | 1.0006 |
| eps_vals[27] | -1.7172 | -3.2235 | -0.2706 | 0.7443 | 0.0081 | 8395.8532 | 1.0004 |
| eps_vals[28] | -3.2382 | -5.9123 | -1.4164 | 1.1438 | 0.0084 | 18511.9769 | 1.0001 |
| eps_vals[29] | 0.0509 | -0.8851 | 0.9768 | 0.4748 | 0.0043 | 12158.4272 | 1.0000 |
| eps_vals[30] | -2.6139 | -6.9053 | -0.0577 | 1.7724 | 0.0124 | 20398.0190 | 1.0000 |
| eps_vals[31] | -1.7665 | -5.8696 | 0.4041 | 1.6391 | 0.0137 | 14296.9845 | 1.0002 |
| eps_vals[32] | -2.2392 | -8.0327 | 0.5862 | 2.3057 | 0.0206 | 12567.1423 | 1.0002 |
| eps_vals[33] | -0.2391 | -1.3408 | 0.3456 | 0.4263 | 0.0031 | 18475.8357 | 1.0001 |
| eps_vals[34] | 0.5405 | -0.6315 | 1.8623 | 0.6275 | 0.0050 | 15989.7777 | 1.0000 |
| eps_vals[35] | -0.3086 | -1.6993 | 1.0290 | 0.6904 | 0.0065 | 11111.8985 | 1.0000 |
| eps_vals[36] | 0.0864 | -0.7457 | 0.9477 | 0.4230 | 0.0031 | 18755.9980 | 1.0000 |
| eps_vals[37] | -0.7921 | -2.7144 | 0.5201 | 0.8289 | 0.0064 | 16794.5155 | 1.0000 |
| eps_vals[38] | 2.3124 | 0.9550 | 3.8011 | 0.7167 | 0.0081 | 7793.1953 | 1.0007 |
| eps_vals[39] | 1.0887 | -0.1095 | 2.2704 | 0.6020 | 0.0052 | 13465.9382 | 1.0004 |
| eps_vals[40] | 2.5228 | 1.3429 | 3.7938 | 0.6223 | 0.0052 | 14490.5531 | 1.0000 |
| eps_vals[41] | 1.8350 | 0.4183 | 3.3431 | 0.7510 | 0.0082 | 8330.6474 | 1.0007 |
| eps_vals[42] | 1.1861 | -0.0261 | 2.3754 | 0.6164 | 0.0054 | 13043.9209 | 1.0002 |
| eps_vals[43] | 2.0497 | 0.8071 | 3.3489 | 0.6564 | 0.0052 | 16002.2964 | 1.0001 |
| eps_vals[44] | 3.1883 | 2.0513 | 4.4747 | 0.6185 | 0.0078 | 6218.0191 | 1.0009 |
| eps_vals[45] | 3.2029 | 1.4208 | 5.2838 | 0.9822 | 0.0066 | 22280.2467 | 0.9999 |
| eps_vals[46] | 0.4331 | -0.7354 | 1.8596 | 0.6501 | 0.0040 | 26199.8462 | 1.0000 |
| eps_vals[47] | 1.0433 | -0.3343 | 3.0001 | 0.8730 | 0.0065 | 18036.7893 | 1.0000 |
| eps_vals[48] | 1.3686 | -0.3708 | 3.0793 | 0.8727 | 0.0067 | 17100.9606 | 1.0003 |
| eps_vals[49] | 0.1307 | -2.2405 | 2.4280 | 1.1350 | 0.0085 | 17666.8092 | 0.9999 |
| eps_vals[50] | -1.3714 | -6.7178 | 1.3336 | 2.0799 | 0.0171 | 14868.6023 | 1.0002 |
| eps_vals[51] | -0.3692 | -1.6066 | 0.7335 | 0.5877 | 0.0048 | 15052.4305 | 1.0000 |
| eps_vals[52] | -0.2887 | -1.3753 | 0.8212 | 0.5556 | 0.0063 | 7843.2809 | 1.0003 |
| eps_vals[53] | -0.0878 | -0.8419 | 0.6652 | 0.3774 | 0.0027 | 19133.1141 | 0.9999 |
| eps_vals[54] | -0.1483 | -1.3040 | 1.0302 | 0.5681 | 0.0042 | 17876.6793 | 1.0001 |
| eps_vals[55] | 1.7497 | 0.2170 | 3.2837 | 0.7821 | 0.0056 | 19281.6013 | 0.9999 |
| eps_vals[56] | -1.3323 | -2.4788 | -0.1147 | 0.5931 | 0.0051 | 13653.0163 | 1.0003 |
| eps_vals[57] | -1.7129 | -4.9831 | 0.6954 | 1.4609 | 0.0093 | 24497.6173 | 1.0000 |
| eps_vals[58] | 1.8809 | 0.6833 | 3.1030 | 0.6188 | 0.0054 | 13023.4061 | 1.0003 |
| eps_vals[59] | 1.6152 | 0.4302 | 2.9591 | 0.6462 | 0.0058 | 12476.4146 | 1.0001 |
| eps_vals[60] | 0.3432 | -0.9272 | 1.6511 | 0.6601 | 0.0078 | 7176.0557 | 1.0007 |
| eps_vals[61] | 1.3085 | 0.2084 | 2.4230 | 0.5624 | 0.0054 | 11002.2745 | 1.0004 |
| eps_vals[62] | 0.6086 | -1.0103 | 2.1326 | 0.7898 | 0.0057 | 19455.1288 | 1.0000 |
| eps_vals[63] | 0.2750 | -0.6264 | 1.1498 | 0.4467 | 0.0039 | 13283.0380 | 1.0001 |
| eps_vals[64] | -1.4678 | -5.3369 | 0.6926 | 1.5465 | 0.0100 | 23757.9964 | 1.0001 |
| eps_vals[65] | 1.0631 | -0.5747 | 3.5572 | 1.1190 | 0.0121 | 8515.2571 | 1.0004 |
| eps_vals[66] | 0.2109 | -0.3943 | 1.0156 | 0.3469 | 0.0033 | 11307.3588 | 0.9999 |
| eps_vals[67] | 0.5839 | -0.2994 | 1.6371 | 0.4976 | 0.0068 | 5341.3752 | 1.0005 |
| eps_vals[68] | 0.0248 | -0.7048 | 0.7726 | 0.3485 | 0.0030 | 13665.6409 | 1.0001 |
| eps_vals[69] | -0.0334 | -1.5480 | 1.3710 | 0.7386 | 0.0083 | 7844.6994 | 1.0005 |
| eps_vals[70] | -0.2693 | -1.2806 | 0.6881 | 0.4965 | 0.0045 | 11991.3322 | 1.0006 |
| eps_vals[71] | 0.0211 | -1.2829 | 1.2043 | 0.6194 | 0.0041 | 23157.0780 | 1.0000 |
| eps_vals[72] | 0.0879 | -0.5123 | 0.8090 | 0.3128 | 0.0021 | 22158.0094 | 0.9998 |
| eps_vals[73] | -2.9957 | -7.1359 | -0.4463 | 1.7185 | 0.0138 | 15576.0261 | 1.0003 |
| eps_vals[74] | -0.4909 | -1.8496 | 0.7021 | 0.6474 | 0.0052 | 15364.3147 | 1.0005 |
| eps_vals[75] | -0.5290 | -1.9679 | 0.8146 | 0.7016 | 0.0054 | 17004.1947 | 1.0001 |
| eps_vals[76] | -0.7904 | -1.8378 | 0.3258 | 0.5517 | 0.0077 | 5180.9399 | 1.0011 |
| eps_vals[77] | -0.7415 | -1.6357 | 0.1216 | 0.4435 | 0.0044 | 9974.1309 | 1.0008 |
| eps_vals[78] | 0.2157 | -0.8161 | 1.3789 | 0.5434 | 0.0046 | 13843.9311 | 1.0002 |
| eps_vals[79] | -2.9637 | -6.1229 | -0.6975 | 1.3806 | 0.0100 | 18993.9880 | 1.0005 |
| eps_vals[80] | -2.2774 | -3.8916 | -0.9264 | 0.7561 | 0.0061 | 15218.4289 | 1.0008 |
| eps_vals[81] | -1.9100 | -4.7244 | 0.1079 | 1.2296 | 0.0078 | 24835.6550 | 1.0000 |
| eps_vals[82] | 0.9992 | -0.0861 | 2.2113 | 0.5885 | 0.0077 | 5788.9060 | 1.0010 |
| eps_vals[83] | 0.5747 | -0.2294 | 1.4227 | 0.4174 | 0.0045 | 8673.4229 | 1.0008 |
| eps_vals[84] | 0.1467 | -0.7979 | 1.0646 | 0.4733 | 0.0042 | 12932.3187 | 1.0002 |
| eps_vals[85] | -2.5523 | -6.2756 | 0.1871 | 1.6387 | 0.0106 | 23774.3866 | 1.0003 |
| eps_vals[86] | -2.6535 | -5.9171 | -0.3719 | 1.4127 | 0.0094 | 22728.1479 | 0.9999 |
| eps_vals[87] | -2.0511 | -4.8017 | -0.0874 | 1.2057 | 0.0079 | 23569.6341 | 0.9999 |
| eps_vals[88] | -1.4327 | -2.3923 | -0.4989 | 0.4793 | 0.0043 | 12191.4357 | 1.0003 |
| eps_vals[89] | -1.7021 | -5.9856 | 0.6313 | 1.7504 | 0.0136 | 16524.4263 | 1.0000 |
| eps_vals[90] | -1.6785 | -7.7593 | 1.3198 | 2.3755 | 0.0201 | 13946.0567 | 1.0001 |
| eps_vals[91] | -0.1098 | -1.1002 | 0.6114 | 0.4019 | 0.0028 | 20329.5705 | 0.9998 |
| eps_vals[92] | -1.0925 | -2.2745 | -0.0607 | 0.5547 | 0.0046 | 14504.1057 | 1.0001 |
| eps_vals[93] | -1.0683 | -2.1011 | 0.0003 | 0.5241 | 0.0062 | 7052.6443 | 1.0002 |
| eps_vals[94] | -0.9693 | -1.9173 | -0.1941 | 0.4415 | 0.0032 | 18518.4813 | 1.0000 |
| eps_vals[95] | -0.9817 | -2.7227 | 0.2389 | 0.7602 | 0.0059 | 16763.7921 | 1.0002 |
| eps_vals[96] | 2.2634 | 1.1459 | 3.5181 | 0.6027 | 0.0077 | 6103.4584 | 1.0008 |
| eps_vals[97] | 2.8453 | 1.7578 | 4.0278 | 0.5797 | 0.0052 | 12318.6576 | 1.0005 |
| eps_vals[98] | 1.6306 | 0.6928 | 2.5800 | 0.4801 | 0.0044 | 12129.8618 | 1.0003 |
| eps_vals[99] | 0.8111 | -1.0582 | 2.8885 | 0.9693 | 0.0080 | 14514.4657 | 1.0000 |
| eps_vals[100] | 0.0996 | -0.5022 | 0.8764 | 0.3255 | 0.0024 | 18562.6133 | 0.9999 |
| eps_vals[101] | 0.4292 | -0.6588 | 1.5139 | 0.5363 | 0.0045 | 14504.6607 | 1.0002 |
| eps_vals[102] | 1.6381 | 0.6825 | 2.7401 | 0.5246 | 0.0064 | 6783.2660 | 1.0002 |
| eps_vals[103] | 0.4233 | -0.2815 | 1.2038 | 0.3764 | 0.0028 | 17958.9441 | 1.0001 |
| eps_vals[104] | 0.5490 | -0.5951 | 1.8890 | 0.6226 | 0.0047 | 17797.9837 | 1.0000 |
| eps_vals[105] | 2.1167 | 1.1337 | 3.2402 | 0.5397 | 0.0075 | 5133.2363 | 1.0013 |
| eps_vals[106] | 1.8006 | 1.0438 | 2.6047 | 0.3941 | 0.0044 | 8081.8627 | 1.0008 |
| eps_vals[107] | 1.2088 | 0.4938 | 1.9545 | 0.3710 | 0.0038 | 9651.3627 | 1.0003 |
| eps_vals[108] | 2.2737 | 1.2005 | 3.4589 | 0.5638 | 0.0050 | 12696.1148 | 1.0002 |
| eps_vals[109] | 0.8754 | -0.3896 | 2.2394 | 0.6673 | 0.0080 | 7030.8217 | 1.0007 |
| eps_vals[110] | -0.0074 | -0.9724 | 0.9738 | 0.4924 | 0.0048 | 10512.7295 | 1.0006 |
| eps_vals[111] | 0.0378 | -0.9382 | 1.0027 | 0.4900 | 0.0043 | 13092.5080 | 1.0002 |
| eps_vals[112] | -1.2001 | -5.3754 | 1.4997 | 1.7478 | 0.0106 | 27432.2301 | 1.0002 |
| eps_vals[113] | 0.6294 | -1.1786 | 2.6125 | 0.9281 | 0.0078 | 13991.5452 | 1.0000 |
| eps_vals[114] | -1.3160 | -6.3846 | 1.2657 | 1.9659 | 0.0162 | 14702.3114 | 1.0004 |
| eps_vals[115] | -0.0305 | -0.7193 | 0.5691 | 0.2952 | 0.0022 | 18281.6396 | 1.0000 |
| eps_vals[116] | 0.5039 | -0.5607 | 1.5824 | 0.5279 | 0.0045 | 13826.2400 | 1.0001 |
| eps_vals[117] | 0.8765 | -0.0333 | 1.8977 | 0.4855 | 0.0061 | 6266.4126 | 1.0002 |
| eps_vals[118] | 0.4178 | -0.2548 | 1.1614 | 0.3588 | 0.0028 | 16875.5026 | 0.9999 |
| eps_vals[119] | 0.3202 | -0.7713 | 1.5205 | 0.5608 | 0.0042 | 17460.9046 | 0.9999 |
| eps_vals[120] | -1.4971 | -6.1230 | 1.3724 | 1.8952 | 0.0115 | 27012.0612 | 0.9999 |
| eps_vals[121] | 1.0856 | -0.0952 | 2.3889 | 0.6334 | 0.0080 | 6347.2765 | 1.0010 |
| eps_vals[122] | 0.6653 | -0.8052 | 2.0476 | 0.7226 | 0.0055 | 17191.7156 | 1.0001 |
| eps_vals[123] | 0.0994 | -1.1377 | 1.2373 | 0.6024 | 0.0048 | 15735.3202 | 1.0002 |
| Rho_1[1,1] | 1.0000 | 1.0000 | 1.0000 | 0.0000 | NA | NA | NA |
| Rho_1[1,2] | 0.3001 | -0.2736 | 0.7674 | 0.2723 | 0.0020 | 18052.3774 | 1.0001 |
| Rho_1[1,3] | 0.1117 | -0.4817 | 0.6588 | 0.2983 | 0.0017 | 31041.4384 | 0.9999 |
| Rho_1[1,4] | 0.0806 | -0.5098 | 0.6352 | 0.3010 | 0.0018 | 28191.1182 | 1.0001 |
| Rho_1[1,5] | 0.0332 | -0.5504 | 0.6018 | 0.3014 | 0.0015 | 38990.1687 | 0.9999 |
| Rho_1[1,6] | -0.0180 | -0.5883 | 0.5620 | 0.3011 | 0.0015 | 38904.5876 | 0.9999 |
| Rho_1[1,7] | 0.1194 | -0.4374 | 0.6347 | 0.2807 | 0.0016 | 31488.5015 | 1.0001 |
| Rho_1[1,8] | -0.0213 | -0.5861 | 0.5462 | 0.2977 | 0.0015 | 41755.3170 | 1.0001 |
| Rho_1[2,1] | 0.3001 | -0.2736 | 0.7674 | 0.2723 | 0.0020 | 18052.3774 | 1.0001 |
| Rho_1[2,2] | 1.0000 | 1.0000 | 1.0000 | 0.0000 | 0.0000 | 19998.4304 | 0.9998 |
| Rho_1[2,3] | 0.0554 | -0.5464 | 0.6191 | 0.3074 | 0.0019 | 26051.3477 | 1.0000 |
| Rho_1[2,4] | 0.1535 | -0.4064 | 0.6510 | 0.2757 | 0.0018 | 24171.3959 | 0.9999 |
| Rho_1[2,5] | 0.0088 | -0.5620 | 0.5856 | 0.2987 | 0.0016 | 35465.5586 | 0.9999 |
| Rho_1[2,6] | -0.0055 | -0.5848 | 0.5716 | 0.3019 | 0.0016 | 35104.1523 | 0.9999 |
| Rho_1[2,7] | 0.0300 | -0.5515 | 0.5858 | 0.2992 | 0.0017 | 32421.6373 | 0.9999 |
| Rho_1[2,8] | -0.0050 | -0.5780 | 0.5627 | 0.2991 | 0.0016 | 34755.0469 | 0.9999 |
| Rho_1[3,1] | 0.1117 | -0.4817 | 0.6588 | 0.2983 | 0.0017 | 31041.4384 | 0.9999 |
| Rho_1[3,2] | 0.0554 | -0.5464 | 0.6191 | 0.3074 | 0.0019 | 26051.3477 | 1.0000 |
| Rho_1[3,3] | 1.0000 | 1.0000 | 1.0000 | 0.0000 | 0.0000 | 12691.2645 | 0.9998 |
| Rho_1[3,4] | 0.0642 | -0.5134 | 0.6224 | 0.2972 | 0.0019 | 24919.1331 | 1.0002 |
| Rho_1[3,5] | 0.0016 | -0.5790 | 0.5840 | 0.3055 | 0.0020 | 23845.2001 | 1.0000 |
| Rho_1[3,6] | -0.0031 | -0.5799 | 0.5744 | 0.2991 | 0.0019 | 25264.3788 | 1.0000 |
| Rho_1[3,7] | 0.0132 | -0.5642 | 0.5812 | 0.2979 | 0.0018 | 25988.8052 | 1.0002 |
| Rho_1[3,8] | -0.0015 | -0.5789 | 0.5690 | 0.3013 | 0.0019 | 24432.2468 | 1.0000 |
| Rho_1[4,1] | 0.0806 | -0.5098 | 0.6352 | 0.3010 | 0.0018 | 28191.1182 | 1.0001 |
| Rho_1[4,2] | 0.1535 | -0.4064 | 0.6510 | 0.2757 | 0.0018 | 24171.3959 | 0.9999 |
| Rho_1[4,3] | 0.0642 | -0.5134 | 0.6224 | 0.2972 | 0.0019 | 24919.1331 | 1.0002 |
| Rho_1[4,4] | 1.0000 | 1.0000 | 1.0000 | 0.0000 | 0.0000 | 16426.2430 | 0.9998 |
| Rho_1[4,5] | -0.0113 | -0.5771 | 0.5640 | 0.2999 | 0.0020 | 21661.9913 | 1.0002 |
| Rho_1[4,6] | 0.0034 | -0.5692 | 0.5781 | 0.3028 | 0.0021 | 21053.4231 | 1.0002 |
| Rho_1[4,7] | -0.0292 | -0.5921 | 0.5434 | 0.2960 | 0.0021 | 20551.8401 | 0.9999 |
| Rho_1[4,8] | 0.0042 | -0.5686 | 0.5838 | 0.3022 | 0.0020 | 21743.1255 | 0.9999 |
| Rho_1[5,1] | 0.0332 | -0.5504 | 0.6018 | 0.3014 | 0.0015 | 38990.1687 | 0.9999 |
| Rho_1[5,2] | 0.0088 | -0.5620 | 0.5856 | 0.2987 | 0.0016 | 35465.5586 | 0.9999 |
| Rho_1[5,3] | 0.0016 | -0.5790 | 0.5840 | 0.3055 | 0.0020 | 23845.2001 | 1.0000 |
| Rho_1[5,4] | -0.0113 | -0.5771 | 0.5640 | 0.2999 | 0.0020 | 21661.9913 | 1.0002 |
| Rho_1[5,5] | 1.0000 | 1.0000 | 1.0000 | 0.0000 | 0.0000 | 1209.2246 | 0.9998 |
| Rho_1[5,6] | 0.0207 | -0.5581 | 0.5910 | 0.3011 | 0.0023 | 16976.9259 | 0.9999 |
| Rho_1[5,7] | 0.0590 | -0.5296 | 0.6162 | 0.2989 | 0.0024 | 16148.0357 | 1.0002 |
| Rho_1[5,8] | -0.0031 | -0.5771 | 0.5730 | 0.3010 | 0.0024 | 16090.7379 | 1.0002 |
| Rho_1[6,1] | -0.0180 | -0.5883 | 0.5620 | 0.3011 | 0.0015 | 38904.5876 | 0.9999 |
| Rho_1[6,2] | -0.0055 | -0.5848 | 0.5716 | 0.3019 | 0.0016 | 35104.1523 | 0.9999 |
| Rho_1[6,3] | -0.0031 | -0.5799 | 0.5744 | 0.2991 | 0.0019 | 25264.3788 | 1.0000 |
| Rho_1[6,4] | 0.0034 | -0.5692 | 0.5781 | 0.3028 | 0.0021 | 21053.4231 | 1.0002 |
| Rho_1[6,5] | 0.0207 | -0.5581 | 0.5910 | 0.3011 | 0.0023 | 16976.9259 | 0.9999 |
| Rho_1[6,6] | 1.0000 | 1.0000 | 1.0000 | 0.0000 | 0.0000 | 16842.1480 | 0.9998 |
| Rho_1[6,7] | -0.0063 | -0.5808 | 0.5678 | 0.2998 | 0.0024 | 15538.2222 | 1.0000 |
| Rho_1[6,8] | 0.0144 | -0.5721 | 0.5967 | 0.3074 | 0.0025 | 14976.7549 | 1.0005 |
| Rho_1[7,1] | 0.1194 | -0.4374 | 0.6347 | 0.2807 | 0.0016 | 31488.5015 | 1.0001 |
| Rho_1[7,2] | 0.0300 | -0.5515 | 0.5858 | 0.2992 | 0.0017 | 32421.6373 | 0.9999 |
| Rho_1[7,3] | 0.0132 | -0.5642 | 0.5812 | 0.2979 | 0.0018 | 25988.8052 | 1.0002 |
| Rho_1[7,4] | -0.0292 | -0.5921 | 0.5434 | 0.2960 | 0.0021 | 20551.8401 | 0.9999 |
| Rho_1[7,5] | 0.0590 | -0.5296 | 0.6162 | 0.2989 | 0.0024 | 16148.0357 | 1.0002 |
| Rho_1[7,6] | -0.0063 | -0.5808 | 0.5678 | 0.2998 | 0.0024 | 15538.2222 | 1.0000 |
| Rho_1[7,7] | 1.0000 | 1.0000 | 1.0000 | 0.0000 | 0.0000 | 13926.6227 | 0.9998 |
| Rho_1[7,8] | -0.0158 | -0.5877 | 0.5584 | 0.3004 | 0.0025 | 14175.9271 | 1.0000 |
| Rho_1[8,1] | -0.0213 | -0.5861 | 0.5462 | 0.2977 | 0.0015 | 41755.3170 | 1.0001 |
| Rho_1[8,2] | -0.0050 | -0.5780 | 0.5627 | 0.2991 | 0.0016 | 34755.0469 | 0.9999 |
| Rho_1[8,3] | -0.0015 | -0.5789 | 0.5690 | 0.3013 | 0.0019 | 24432.2468 | 1.0000 |
| Rho_1[8,4] | 0.0042 | -0.5686 | 0.5838 | 0.3022 | 0.0020 | 21743.1255 | 0.9999 |
| Rho_1[8,5] | -0.0031 | -0.5771 | 0.5730 | 0.3010 | 0.0024 | 16090.7379 | 1.0002 |
| Rho_1[8,6] | 0.0144 | -0.5721 | 0.5967 | 0.3074 | 0.0025 | 14976.7549 | 1.0005 |
| Rho_1[8,7] | -0.0158 | -0.5877 | 0.5584 | 0.3004 | 0.0025 | 14175.9271 | 1.0000 |
| Rho_1[8,8] | 1.0000 | 1.0000 | 1.0000 | 0.0000 | 0.0000 | 17592.9368 | 0.9998 |
| Rho_2[1,1] | 1.0000 | 1.0000 | 1.0000 | 0.0000 | NA | NA | NA |
| Rho_2[1,2] | 0.5482 | 0.1496 | 0.8360 | 0.1790 | 0.0017 | 10980.8086 | 1.0001 |
| Rho_2[1,3] | 0.4438 | 0.0014 | 0.7789 | 0.2018 | 0.0017 | 14273.7974 | 1.0015 |
| Rho_2[1,4] | 0.1309 | -0.3570 | 0.5902 | 0.2470 | 0.0013 | 35060.2384 | 1.0000 |
| Rho_2[1,5] | 0.0154 | -0.5056 | 0.5213 | 0.2688 | 0.0015 | 31967.2337 | 0.9999 |
| Rho_2[1,6] | 0.0038 | -0.5101 | 0.5218 | 0.2681 | 0.0014 | 35442.0915 | 0.9998 |
| Rho_2[1,7] | 0.0041 | -0.5133 | 0.5126 | 0.2677 | 0.0014 | 37203.5589 | 1.0000 |
| Rho_2[1,8] | 0.0141 | -0.4986 | 0.5239 | 0.2667 | 0.0015 | 31854.4668 | 1.0000 |
| Rho_2[1,9] | 0.0178 | -0.5012 | 0.5269 | 0.2679 | 0.0015 | 30882.0997 | 0.9998 |
| Rho_2[1,10] | 0.0565 | -0.4528 | 0.5424 | 0.2597 | 0.0015 | 31282.1324 | 0.9999 |
| Rho_2[1,11] | 0.1012 | -0.4013 | 0.5840 | 0.2554 | 0.0014 | 31077.6544 | 1.0000 |
| Rho_2[2,1] | 0.5482 | 0.1496 | 0.8360 | 0.1790 | 0.0017 | 10980.8086 | 1.0001 |
| Rho_2[2,2] | 1.0000 | 1.0000 | 1.0000 | 0.0000 | 0.0000 | 17030.1951 | 0.9998 |
| Rho_2[2,3] | 0.3343 | -0.0903 | 0.6985 | 0.2043 | 0.0016 | 15661.4161 | 1.0003 |
| Rho_2[2,4] | 0.1913 | -0.2826 | 0.6275 | 0.2365 | 0.0013 | 31771.7641 | 0.9999 |
| Rho_2[2,5] | 0.0168 | -0.5027 | 0.5250 | 0.2666 | 0.0015 | 31000.0175 | 1.0000 |
| Rho_2[2,6] | 0.0035 | -0.5047 | 0.5103 | 0.2643 | 0.0014 | 33944.1056 | 0.9999 |
| Rho_2[2,7] | 0.0030 | -0.5065 | 0.5170 | 0.2668 | 0.0014 | 34522.1917 | 1.0001 |
| Rho_2[2,8] | 0.0139 | -0.5066 | 0.5224 | 0.2669 | 0.0015 | 31818.9629 | 0.9999 |
| Rho_2[2,9] | 0.0181 | -0.4984 | 0.5297 | 0.2685 | 0.0016 | 29909.3796 | 0.9999 |
| Rho_2[2,10] | 0.0579 | -0.4448 | 0.5462 | 0.2585 | 0.0015 | 29608.7359 | 0.9999 |
| Rho_2[2,11] | 0.1032 | -0.4016 | 0.5773 | 0.2527 | 0.0014 | 30983.6621 | 1.0000 |
| Rho_2[3,1] | 0.4438 | 0.0014 | 0.7789 | 0.2018 | 0.0017 | 14273.7974 | 1.0015 |
| Rho_2[3,2] | 0.3343 | -0.0903 | 0.6985 | 0.2043 | 0.0016 | 15661.4161 | 1.0003 |
| Rho_2[3,3] | 1.0000 | 1.0000 | 1.0000 | 0.0000 | 0.0000 | 645.3985 | 0.9998 |
| Rho_2[3,4] | 0.0660 | -0.4300 | 0.5474 | 0.2541 | 0.0014 | 30895.7643 | 0.9999 |
| Rho_2[3,5] | 0.0093 | -0.5131 | 0.5235 | 0.2702 | 0.0016 | 28638.2392 | 0.9999 |
| Rho_2[3,6] | 0.0029 | -0.5175 | 0.5239 | 0.2696 | 0.0016 | 30243.0002 | 0.9999 |
| Rho_2[3,7] | 0.0031 | -0.5038 | 0.5137 | 0.2652 | 0.0015 | 32866.7168 | 0.9999 |
| Rho_2[3,8] | 0.0089 | -0.5023 | 0.5186 | 0.2669 | 0.0016 | 29063.4154 | 0.9999 |
| Rho_2[3,9] | 0.0072 | -0.5146 | 0.5148 | 0.2678 | 0.0017 | 25575.1491 | 0.9999 |
| Rho_2[3,10] | 0.0301 | -0.4829 | 0.5307 | 0.2641 | 0.0016 | 27280.2322 | 1.0000 |
| Rho_2[3,11] | 0.0532 | -0.4674 | 0.5490 | 0.2637 | 0.0015 | 30500.9629 | 0.9999 |
| Rho_2[4,1] | 0.1309 | -0.3570 | 0.5902 | 0.2470 | 0.0013 | 35060.2384 | 1.0000 |
| Rho_2[4,2] | 0.1913 | -0.2826 | 0.6275 | 0.2365 | 0.0013 | 31771.7641 | 0.9999 |
| Rho_2[4,3] | 0.0660 | -0.4300 | 0.5474 | 0.2541 | 0.0014 | 30895.7643 | 0.9999 |
| Rho_2[4,4] | 1.0000 | 1.0000 | 1.0000 | 0.0000 | 0.0000 | 19385.0065 | 0.9998 |
| Rho_2[4,5] | 0.0057 | -0.5060 | 0.5247 | 0.2679 | 0.0018 | 23273.1786 | 0.9999 |
| Rho_2[4,6] | 0.0020 | -0.5078 | 0.5229 | 0.2678 | 0.0018 | 23396.0266 | 0.9999 |
| Rho_2[4,7] | 0.0000 | -0.5146 | 0.5210 | 0.2687 | 0.0018 | 22532.9292 | 0.9999 |
| Rho_2[4,8] | 0.0051 | -0.5183 | 0.5250 | 0.2705 | 0.0018 | 23517.5829 | 0.9999 |
| Rho_2[4,9] | 0.0050 | -0.5096 | 0.5179 | 0.2670 | 0.0018 | 22465.9113 | 0.9999 |
| Rho_2[4,10] | 0.0132 | -0.5058 | 0.5226 | 0.2684 | 0.0018 | 22040.7914 | 0.9999 |
| Rho_2[4,11] | 0.0222 | -0.4948 | 0.5285 | 0.2662 | 0.0018 | 22725.7185 | 1.0000 |
| Rho_2[5,1] | 0.0154 | -0.5056 | 0.5213 | 0.2688 | 0.0015 | 31967.2337 | 0.9999 |
| Rho_2[5,2] | 0.0168 | -0.5027 | 0.5250 | 0.2666 | 0.0015 | 31000.0175 | 1.0000 |
| Rho_2[5,3] | 0.0093 | -0.5131 | 0.5235 | 0.2702 | 0.0016 | 28638.2392 | 0.9999 |
| Rho_2[5,4] | 0.0057 | -0.5060 | 0.5247 | 0.2679 | 0.0018 | 23273.1786 | 0.9999 |
| Rho_2[5,5] | 1.0000 | 1.0000 | 1.0000 | 0.0000 | 0.0000 | 10716.6813 | 0.9998 |
| Rho_2[5,6] | 0.0721 | -0.4544 | 0.5608 | 0.2611 | 0.0019 | 18277.7613 | 1.0000 |
| Rho_2[5,7] | 0.0481 | -0.4742 | 0.5502 | 0.2658 | 0.0019 | 20224.2818 | 0.9998 |
| Rho_2[5,8] | 0.0437 | -0.4559 | 0.5351 | 0.2561 | 0.0020 | 17215.1924 | 0.9999 |
| Rho_2[5,9] | 0.1315 | -0.3676 | 0.6003 | 0.2511 | 0.0019 | 16895.8311 | 1.0004 |
| Rho_2[5,10] | 0.1041 | -0.4066 | 0.5857 | 0.2562 | 0.0019 | 18419.6083 | 1.0000 |
| Rho_2[5,11] | 0.1380 | -0.3766 | 0.6142 | 0.2589 | 0.0019 | 18128.1709 | 1.0000 |
| Rho_2[6,1] | 0.0038 | -0.5101 | 0.5218 | 0.2681 | 0.0014 | 35442.0915 | 0.9998 |
| Rho_2[6,2] | 0.0035 | -0.5047 | 0.5103 | 0.2643 | 0.0014 | 33944.1056 | 0.9999 |
| Rho_2[6,3] | 0.0029 | -0.5175 | 0.5239 | 0.2696 | 0.0016 | 30243.0002 | 0.9999 |
| Rho_2[6,4] | 0.0020 | -0.5078 | 0.5229 | 0.2678 | 0.0018 | 23396.0266 | 0.9999 |
| Rho_2[6,5] | 0.0721 | -0.4544 | 0.5608 | 0.2611 | 0.0019 | 18277.7613 | 1.0000 |
| Rho_2[6,6] | 1.0000 | 1.0000 | 1.0000 | 0.0000 | 0.0000 | 17039.3055 | 0.9998 |
| Rho_2[6,7] | 0.0356 | -0.4844 | 0.5474 | 0.2674 | 0.0019 | 18925.7743 | 1.0000 |
| Rho_2[6,8] | 0.0001 | -0.5053 | 0.5059 | 0.2628 | 0.0021 | 16393.8513 | 0.9999 |
| Rho_2[6,9] | 0.0289 | -0.4883 | 0.5338 | 0.2656 | 0.0022 | 14685.5089 | 1.0001 |
| Rho_2[6,10] | 0.0220 | -0.4844 | 0.5177 | 0.2596 | 0.0021 | 15713.7031 | 1.0001 |
| Rho_2[6,11] | 0.0707 | -0.4503 | 0.5644 | 0.2643 | 0.0021 | 16273.3593 | 1.0001 |
| Rho_2[7,1] | 0.0041 | -0.5133 | 0.5126 | 0.2677 | 0.0014 | 37203.5589 | 1.0000 |
| Rho_2[7,2] | 0.0030 | -0.5065 | 0.5170 | 0.2668 | 0.0014 | 34522.1917 | 1.0001 |
| Rho_2[7,3] | 0.0031 | -0.5038 | 0.5137 | 0.2652 | 0.0015 | 32866.7168 | 0.9999 |
| Rho_2[7,4] | 0.0000 | -0.5146 | 0.5210 | 0.2687 | 0.0018 | 22532.9292 | 0.9999 |
| Rho_2[7,5] | 0.0481 | -0.4742 | 0.5502 | 0.2658 | 0.0019 | 20224.2818 | 0.9998 |
| Rho_2[7,6] | 0.0356 | -0.4844 | 0.5474 | 0.2674 | 0.0019 | 18925.7743 | 1.0000 |
| Rho_2[7,7] | 1.0000 | 1.0000 | 1.0000 | 0.0000 | 0.0000 | 18171.8755 | 0.9998 |
| Rho_2[7,8] | -0.0058 | -0.5256 | 0.5132 | 0.2676 | 0.0022 | 15336.4456 | 0.9999 |
| Rho_2[7,9] | 0.0351 | -0.4729 | 0.5404 | 0.2637 | 0.0022 | 14400.1004 | 1.0001 |
| Rho_2[7,10] | 0.0168 | -0.4941 | 0.5241 | 0.2644 | 0.0022 | 14512.5510 | 0.9999 |
| Rho_2[7,11] | 0.0425 | -0.4684 | 0.5451 | 0.2642 | 0.0021 | 15715.0830 | 0.9998 |
| Rho_2[8,1] | 0.0141 | -0.4986 | 0.5239 | 0.2667 | 0.0015 | 31854.4668 | 1.0000 |
| Rho_2[8,2] | 0.0139 | -0.5066 | 0.5224 | 0.2669 | 0.0015 | 31818.9629 | 0.9999 |
| Rho_2[8,3] | 0.0089 | -0.5023 | 0.5186 | 0.2669 | 0.0016 | 29063.4154 | 0.9999 |
| Rho_2[8,4] | 0.0051 | -0.5183 | 0.5250 | 0.2705 | 0.0018 | 23517.5829 | 0.9999 |
| Rho_2[8,5] | 0.0437 | -0.4559 | 0.5351 | 0.2561 | 0.0020 | 17215.1924 | 0.9999 |
| Rho_2[8,6] | 0.0001 | -0.5053 | 0.5059 | 0.2628 | 0.0021 | 16393.8513 | 0.9999 |
| Rho_2[8,7] | -0.0058 | -0.5256 | 0.5132 | 0.2676 | 0.0022 | 15336.4456 | 0.9999 |
| Rho_2[8,8] | 1.0000 | 1.0000 | 1.0000 | 0.0000 | 0.0000 | 17513.9709 | 0.9998 |
| Rho_2[8,9] | 0.1282 | -0.3787 | 0.5989 | 0.2537 | 0.0020 | 15610.8538 | 0.9999 |
| Rho_2[8,10] | 0.1687 | -0.3461 | 0.6358 | 0.2564 | 0.0020 | 15654.1526 | 1.0004 |
| Rho_2[8,11] | 0.0739 | -0.4321 | 0.5715 | 0.2603 | 0.0021 | 15668.7009 | 1.0000 |
| Rho_2[9,1] | 0.0178 | -0.5012 | 0.5269 | 0.2679 | 0.0015 | 30882.0997 | 0.9998 |
| Rho_2[9,2] | 0.0181 | -0.4984 | 0.5297 | 0.2685 | 0.0016 | 29909.3796 | 0.9999 |
| Rho_2[9,3] | 0.0072 | -0.5146 | 0.5148 | 0.2678 | 0.0017 | 25575.1491 | 0.9999 |
| Rho_2[9,4] | 0.0050 | -0.5096 | 0.5179 | 0.2670 | 0.0018 | 22465.9113 | 0.9999 |
| Rho_2[9,5] | 0.1315 | -0.3676 | 0.6003 | 0.2511 | 0.0019 | 16895.8311 | 1.0004 |
| Rho_2[9,6] | 0.0289 | -0.4883 | 0.5338 | 0.2656 | 0.0022 | 14685.5089 | 1.0001 |
| Rho_2[9,7] | 0.0351 | -0.4729 | 0.5404 | 0.2637 | 0.0022 | 14400.1004 | 1.0001 |
| Rho_2[9,8] | 0.1282 | -0.3787 | 0.5989 | 0.2537 | 0.0020 | 15610.8538 | 0.9999 |
| Rho_2[9,9] | 1.0000 | 1.0000 | 1.0000 | 0.0000 | 0.0000 | 15461.2767 | 0.9998 |
| Rho_2[9,10] | 0.1583 | -0.3410 | 0.6197 | 0.2497 | 0.0020 | 14991.0221 | 1.0000 |
| Rho_2[9,11] | 0.1379 | -0.3806 | 0.6161 | 0.2582 | 0.0021 | 15038.4377 | 1.0000 |
| Rho_2[10,1] | 0.0565 | -0.4528 | 0.5424 | 0.2597 | 0.0015 | 31282.1324 | 0.9999 |
| Rho_2[10,2] | 0.0579 | -0.4448 | 0.5462 | 0.2585 | 0.0015 | 29608.7359 | 0.9999 |
| Rho_2[10,3] | 0.0301 | -0.4829 | 0.5307 | 0.2641 | 0.0016 | 27280.2322 | 1.0000 |
| Rho_2[10,4] | 0.0132 | -0.5058 | 0.5226 | 0.2684 | 0.0018 | 22040.7914 | 0.9999 |
| Rho_2[10,5] | 0.1041 | -0.4066 | 0.5857 | 0.2562 | 0.0019 | 18419.6083 | 1.0000 |
| Rho_2[10,6] | 0.0220 | -0.4844 | 0.5177 | 0.2596 | 0.0021 | 15713.7031 | 1.0001 |
| Rho_2[10,7] | 0.0168 | -0.4941 | 0.5241 | 0.2644 | 0.0022 | 14512.5510 | 0.9999 |
| Rho_2[10,8] | 0.1687 | -0.3461 | 0.6358 | 0.2564 | 0.0020 | 15654.1526 | 1.0004 |
| Rho_2[10,9] | 0.1583 | -0.3410 | 0.6197 | 0.2497 | 0.0020 | 14991.0221 | 1.0000 |
| Rho_2[10,10] | 1.0000 | 1.0000 | 1.0000 | 0.0000 | 0.0000 | 15526.4065 | 0.9998 |
| Rho_2[10,11] | 0.1407 | -0.3716 | 0.6133 | 0.2566 | 0.0022 | 13703.6155 | 1.0004 |
| Rho_2[11,1] | 0.1012 | -0.4013 | 0.5840 | 0.2554 | 0.0014 | 31077.6544 | 1.0000 |
| Rho_2[11,2] | 0.1032 | -0.4016 | 0.5773 | 0.2527 | 0.0014 | 30983.6621 | 1.0000 |
| Rho_2[11,3] | 0.0532 | -0.4674 | 0.5490 | 0.2637 | 0.0015 | 30500.9629 | 0.9999 |
| Rho_2[11,4] | 0.0222 | -0.4948 | 0.5285 | 0.2662 | 0.0018 | 22725.7185 | 1.0000 |
| Rho_2[11,5] | 0.1380 | -0.3766 | 0.6142 | 0.2589 | 0.0019 | 18128.1709 | 1.0000 |
| Rho_2[11,6] | 0.0707 | -0.4503 | 0.5644 | 0.2643 | 0.0021 | 16273.3593 | 1.0001 |
| Rho_2[11,7] | 0.0425 | -0.4684 | 0.5451 | 0.2642 | 0.0021 | 15715.0830 | 0.9998 |
| Rho_2[11,8] | 0.0739 | -0.4321 | 0.5715 | 0.2603 | 0.0021 | 15668.7009 | 1.0000 |
| Rho_2[11,9] | 0.1379 | -0.3806 | 0.6161 | 0.2582 | 0.0021 | 15038.4377 | 1.0000 |
| Rho_2[11,10] | 0.1407 | -0.3716 | 0.6133 | 0.2566 | 0.0022 | 13703.6155 | 1.0004 |
| Rho_2[11,11] | 1.0000 | 1.0000 | 1.0000 | 0.0000 | 0.0000 | 17357.3504 | 0.9998 |
| p[1] | 0.2796 | 0.1261 | 0.4584 | 0.0851 | 0.0011 | 5613.5087 | 1.0004 |
| p[2] | 0.0822 | 0.0253 | 0.1748 | 0.0391 | 0.0005 | 5347.7291 | 1.0012 |
| p[3] | 0.1248 | 0.0574 | 0.2221 | 0.0422 | 0.0005 | 7958.4756 | 1.0009 |
| p[4] | 0.1243 | 0.0602 | 0.2163 | 0.0399 | 0.0004 | 9561.0108 | 1.0003 |
| p[5] | 0.0755 | 0.0218 | 0.1737 | 0.0396 | 0.0003 | 12958.7977 | 1.0001 |
| p[6] | 0.0835 | 0.0106 | 0.2845 | 0.0738 | 0.0007 | 12094.1805 | 1.0001 |
| p[7] | 0.0758 | 0.0050 | 0.2500 | 0.0684 | 0.0007 | 8901.0988 | 1.0003 |
| p[8] | 0.2100 | 0.1302 | 0.3044 | 0.0443 | 0.0003 | 18567.9065 | 1.0000 |
| p[9] | 0.3024 | 0.1288 | 0.5400 | 0.1020 | 0.0009 | 14147.8564 | 1.0000 |
| p[10] | 0.3761 | 0.2419 | 0.5232 | 0.0704 | 0.0006 | 15937.2681 | 1.0000 |
| p[11] | 0.2600 | 0.0980 | 0.4894 | 0.0955 | 0.0007 | 17247.1597 | 1.0000 |
| p[12] | 0.1692 | 0.0622 | 0.3100 | 0.0652 | 0.0009 | 5593.4252 | 1.0006 |
| p[13] | 0.0442 | 0.0133 | 0.1016 | 0.0234 | 0.0003 | 5660.1724 | 1.0011 |
| p[14] | 0.0680 | 0.0308 | 0.1285 | 0.0251 | 0.0003 | 8430.5735 | 1.0009 |
| p[15] | 0.0676 | 0.0330 | 0.1247 | 0.0238 | 0.0002 | 10363.5718 | 1.0005 |
| p[16] | 0.0398 | 0.0115 | 0.0925 | 0.0213 | 0.0002 | 13728.6781 | 1.0001 |
| p[17] | 0.0471 | 0.0051 | 0.1754 | 0.0496 | 0.0005 | 11878.5458 | 1.0002 |
| p[18] | 0.0408 | 0.0024 | 0.1288 | 0.0377 | 0.0004 | 9138.1431 | 1.0003 |
| p[19] | 0.1211 | 0.0584 | 0.1957 | 0.0343 | 0.0003 | 11702.9623 | 0.9999 |
| p[20] | 0.1880 | 0.0618 | 0.4019 | 0.0870 | 0.0008 | 12369.4638 | 1.0000 |
| p[21] | 0.2376 | 0.1281 | 0.3824 | 0.0614 | 0.0005 | 13637.3657 | 1.0001 |
| p[22] | 0.1580 | 0.0467 | 0.3484 | 0.0776 | 0.0006 | 14459.4433 | 1.0000 |

Table S10: Parameter estimates for 1997-type severity ($\beta_{k\mathcal{l}}$: beta_sero_prior, $\beta_{j}$: beta_reg, $\alpha$: intercept, $\sigma_{jk\mathcal{l}}$: sigma, $\epsilon_{ijk\mathcal{l}}$: eps_vals, $P_{j}$: Rho_1 and Rho_2, $p_{jk\mathcal{l}}$: p)

## Trace Plots and Parameter Estimates (2009-type Severity)

Figure S5: Trace plot for random effects $\epsilon_{ijk\mathcal{l}}$ for 2009-type severity

Figure S6: Trace plot for prior exposure-serotype effects $\beta_{k\mathcal{l}}$, region effect $\beta_{j}$, intercept $\alpha$, and standard deviation of random effects $\sigma_{jk\mathcal{l}}$ for 2009-type severity.

Figure S7: Trace plot for region-specific correlation matrices $P_{j}$ for 2009-type severity (including diagonal elements that are always 1 by definition).

| Parameter | Mean | 2.50% | 97.50% | SD | SE_Mean | ESS | Rhat |
| --- | --- | --- | --- | --- | --- | --- | --- |
| beta_sero_prior[1] | -1.3982 | -3.0931 | 0.2414 | 0.8483 | 0.0109 | 6013.9092 | 1.0000 |
| beta_sero_prior[2] | -0.5956 | -1.8305 | 0.6637 | 0.6236 | 0.0076 | 6788.4931 | 1.0003 |
| beta_sero_prior[3] | -0.1517 | -1.4582 | 1.1902 | 0.6701 | 0.0072 | 8616.8102 | 1.0001 |
| beta_sero_prior[4] | -0.0774 | -1.2895 | 1.1691 | 0.6101 | 0.0069 | 7800.2995 | 0.9999 |
| beta_sero_prior[5] | -0.8791 | -3.3982 | 1.3478 | 1.1962 | 0.0098 | 14885.5186 | 1.0000 |
| beta_sero_prior[6] | 0.4960 | -1.2678 | 2.1804 | 0.8543 | 0.0092 | 8538.0588 | 1.0001 |
| beta_sero_prior[7] | 0.2396 | -1.8905 | 2.3516 | 1.0373 | 0.0099 | 10883.2645 | 1.0001 |
| beta_sero_prior[8] | -0.7116 | -2.7577 | 0.9987 | 0.9560 | 0.0089 | 11628.2652 | 1.0000 |
| beta_sero_prior[9] | -1.0426 | -2.9983 | 0.7978 | 0.9583 | 0.0090 | 11345.4777 | 0.9999 |
| beta_reg | 0.1217 | -0.9042 | 0.9933 | 0.4836 | 0.0060 | 6432.3601 | 1.0009 |
| intercept | -2.0640 | -3.1650 | -1.0898 | 0.5304 | 0.0068 | 6166.1309 | 1.0003 |
| sigma[1] | 1.9711 | 0.7326 | 4.2884 | 0.9323 | 0.0092 | 10264.6042 | 1.0005 |
| sigma[2] | 2.0539 | 1.0210 | 3.8131 | 0.7163 | 0.0064 | 12416.3012 | 0.9999 |
| sigma[3] | 1.2262 | 0.0624 | 3.5310 | 0.9129 | 0.0080 | 12995.8388 | 1.0001 |
| sigma[4] | 0.6906 | 0.0183 | 2.5352 | 0.6948 | 0.0069 | 10097.8173 | 1.0001 |
| sigma[5] | 1.5066 | 0.3597 | 3.8703 | 0.9298 | 0.0068 | 18851.7982 | 1.0002 |
| sigma[6] | 1.8267 | 0.1763 | 4.4795 | 1.1254 | 0.0086 | 17301.7611 | 1.0001 |
| sigma[7] | 1.4503 | 0.0909 | 3.8693 | 0.9885 | 0.0098 | 10146.2602 | 1.0002 |
| sigma[8] | 2.2717 | 0.8449 | 4.6093 | 0.9785 | 0.0069 | 20404.0716 | 0.9999 |
| sigma[9] | 1.8627 | 0.4693 | 4.1210 | 0.9637 | 0.0114 | 7161.1382 | 1.0002 |
| sigma[10] | 0.9837 | 0.1481 | 2.4934 | 0.6081 | 0.0074 | 6678.5710 | 1.0001 |
| sigma[11] | 1.1735 | 0.3482 | 2.6200 | 0.5870 | 0.0055 | 11525.5614 | 1.0001 |
| sigma[12] | 1.0802 | 0.0789 | 3.0221 | 0.7779 | 0.0073 | 11353.9404 | 1.0006 |
| sigma[13] | 1.8112 | 0.1295 | 4.5762 | 1.1694 | 0.0093 | 15837.8376 | 1.0001 |
| sigma[14] | 1.1052 | 0.0644 | 3.2117 | 0.8325 | 0.0074 | 12724.5640 | 0.9999 |
| sigma[15] | 1.4340 | 0.0564 | 4.2200 | 1.1237 | 0.0089 | 15764.5591 | 0.9999 |
| sigma[16] | 1.8883 | 0.5813 | 4.0469 | 0.8896 | 0.0074 | 14582.3683 | 1.0000 |
| sigma[17] | 1.5715 | 0.0672 | 4.4419 | 1.1717 | 0.0092 | 16379.6424 | 1.0000 |
| eps_vals[1] | -1.5484 | -6.0204 | 1.0001 | 1.8018 | 0.0150 | 14366.4968 | 1.0002 |
| eps_vals[2] | -1.3811 | -5.9412 | 1.2958 | 1.8421 | 0.0148 | 15506.1746 | 1.0001 |
| eps_vals[3] | -1.7284 | -5.6124 | 0.7728 | 1.6292 | 0.0110 | 21794.7578 | 0.9999 |
| eps_vals[4] | -0.1304 | -2.5689 | 1.8518 | 1.0948 | 0.0099 | 12222.3310 | 1.0000 |
| eps_vals[5] | 1.0562 | -0.0558 | 2.2725 | 0.5927 | 0.0067 | 7799.4454 | 1.0006 |
| eps_vals[6] | -0.1981 | -1.7705 | 1.2380 | 0.7175 | 0.0057 | 16057.6584 | 0.9999 |
| eps_vals[7] | 0.2987 | -0.3937 | 1.5846 | 0.4961 | 0.0051 | 9343.6137 | 1.0000 |
| eps_vals[8] | -1.1168 | -5.6139 | 1.5518 | 1.8130 | 0.0144 | 15854.5942 | 1.0003 |
| eps_vals[9] | 0.1458 | -1.0859 | 1.7589 | 0.6581 | 0.0049 | 18301.3805 | 0.9999 |
| eps_vals[10] | -0.8825 | -5.4147 | 1.9237 | 1.8512 | 0.0135 | 18678.0474 | 0.9999 |
| eps_vals[11] | -0.3434 | -2.7115 | 0.8063 | 0.8761 | 0.0065 | 18025.3544 | 1.0001 |
| eps_vals[12] | -0.4912 | -2.5470 | 1.5714 | 0.9754 | 0.0088 | 12250.8896 | 1.0003 |
| eps_vals[13] | 0.4164 | -1.3702 | 2.4128 | 0.9550 | 0.0080 | 14100.3229 | 1.0001 |
| eps_vals[14] | -0.9149 | -2.6268 | 0.6851 | 0.8426 | 0.0099 | 7177.8564 | 1.0005 |
| eps_vals[15] | -1.8650 | -4.0321 | -0.1700 | 0.9777 | 0.0079 | 15347.5779 | 1.0002 |
| eps_vals[16] | -1.2773 | -5.9530 | 1.2722 | 1.8277 | 0.0123 | 22224.4864 | 0.9999 |
| eps_vals[17] | -1.3805 | -5.3087 | 0.4505 | 1.5248 | 0.0116 | 17161.3259 | 1.0001 |
| eps_vals[18] | -2.4110 | -6.9662 | 0.3189 | 1.8511 | 0.0126 | 21541.8500 | 1.0000 |
| eps_vals[19] | 1.0233 | -1.1086 | 3.7757 | 1.2218 | 0.0099 | 15371.4919 | 1.0001 |
| eps_vals[20] | 0.6873 | -0.6532 | 2.6488 | 0.8368 | 0.0080 | 10997.1780 | 0.9999 |
| eps_vals[21] | 0.4139 | -1.3519 | 2.6470 | 0.9634 | 0.0081 | 14089.9371 | 1.0002 |
| eps_vals[22] | 1.9239 | 0.3853 | 3.5293 | 0.7994 | 0.0083 | 9270.7061 | 1.0003 |
| eps_vals[23] | 0.1734 | -2.1060 | 2.3822 | 1.0605 | 0.0082 | 16814.6815 | 0.9999 |
| eps_vals[24] | 2.1864 | 0.7904 | 3.6875 | 0.7375 | 0.0070 | 11043.3220 | 1.0004 |
| eps_vals[25] | 0.6999 | -0.4461 | 2.2738 | 0.7137 | 0.0073 | 9576.5545 | 1.0002 |
| eps_vals[26] | 0.9737 | -0.3088 | 2.6226 | 0.7881 | 0.0089 | 7778.9440 | 1.0002 |
| eps_vals[27] | -0.7218 | -4.9818 | 2.0711 | 1.7413 | 0.0122 | 20423.7766 | 0.9999 |
| eps_vals[28] | -0.8187 | -3.3054 | 0.5016 | 0.9915 | 0.0083 | 14234.3822 | 1.0001 |
| eps_vals[29] | 0.6006 | -0.9440 | 2.2965 | 0.8143 | 0.0059 | 18814.7212 | 0.9999 |
| eps_vals[30] | -0.5848 | -2.6063 | 0.6480 | 0.8328 | 0.0071 | 13667.7897 | 1.0004 |
| eps_vals[31] | 1.0453 | -0.0829 | 2.5511 | 0.7027 | 0.0076 | 8447.7116 | 1.0000 |
| eps_vals[32] | -0.9400 | -5.7807 | 1.9101 | 1.9139 | 0.0131 | 21251.4297 | 0.9999 |
| eps_vals[33] | -0.0105 | -1.1916 | 1.2108 | 0.5837 | 0.0045 | 17049.7698 | 1.0003 |
| eps_vals[34] | -0.0621 | -1.3190 | 1.0714 | 0.5948 | 0.0049 | 15032.8066 | 1.0002 |
| eps_vals[35] | 2.0169 | 0.2597 | 3.9716 | 0.9780 | 0.0131 | 5536.7878 | 1.0002 |
| eps_vals[36] | 0.6623 | -0.5200 | 1.8805 | 0.6132 | 0.0049 | 15852.9653 | 1.0001 |
| eps_vals[37] | 0.3705 | -0.8500 | 1.6606 | 0.6158 | 0.0049 | 16077.6851 | 0.9999 |
| eps_vals[38] | -0.6607 | -3.1527 | 0.7665 | 1.0090 | 0.0083 | 14769.7993 | 1.0000 |
| eps_vals[39] | 0.6214 | -1.3594 | 2.7251 | 0.9680 | 0.0087 | 12277.4853 | 1.0002 |
| eps_vals[40] | 1.1646 | -0.3544 | 3.3097 | 0.9546 | 0.0092 | 10750.6025 | 1.0000 |
| eps_vals[41] | 0.2282 | -0.7527 | 1.4116 | 0.5384 | 0.0062 | 7484.5815 | 1.0001 |
| eps_vals[42] | 1.9933 | 0.2597 | 3.9748 | 0.9381 | 0.0079 | 14055.9355 | 1.0001 |
| eps_vals[43] | -2.7777 | -7.1543 | -0.3592 | 1.7652 | 0.0171 | 10619.0909 | 1.0000 |
| eps_vals[44] | -2.1288 | -3.8496 | -0.5790 | 0.8334 | 0.0074 | 12788.0700 | 1.0007 |
| eps_vals[45] | -0.9152 | -5.3200 | 1.2341 | 1.6563 | 0.0120 | 19142.5093 | 1.0000 |
| eps_vals[46] | -1.7907 | -5.7492 | 0.4377 | 1.5868 | 0.0108 | 21513.4610 | 0.9999 |
| eps_vals[47] | -1.2524 | -5.9351 | 0.8752 | 1.7658 | 0.0133 | 17505.1564 | 1.0000 |
| eps_vals[48] | 0.9570 | -0.5694 | 2.4715 | 0.7654 | 0.0097 | 6195.6592 | 1.0004 |
| eps_vals[49] | 0.3423 | -0.7150 | 1.5192 | 0.5638 | 0.0065 | 7619.9432 | 1.0006 |
| eps_vals[50] | 0.2203 | -0.4457 | 1.3921 | 0.4516 | 0.0046 | 9460.7765 | 0.9999 |
| eps_vals[51] | 1.4286 | -0.0830 | 3.1965 | 0.8712 | 0.0120 | 5274.1254 | 1.0003 |
| eps_vals[52] | -0.2683 | -1.1827 | 0.7518 | 0.4730 | 0.0042 | 12780.0096 | 1.0008 |
| eps_vals[53] | -0.6401 | -2.0977 | 0.5078 | 0.6612 | 0.0054 | 14734.2136 | 1.0003 |
| eps_vals[54] | -1.2699 | -5.8767 | 1.1912 | 1.7862 | 0.0129 | 19275.9368 | 0.9999 |
| eps_vals[55] | -0.3889 | -2.5174 | 1.2183 | 0.8997 | 0.0068 | 17676.2981 | 1.0000 |
| eps_vals[56] | -0.8077 | -5.0549 | 1.2714 | 1.5809 | 0.0112 | 19853.5815 | 0.9999 |
| eps_vals[57] | -0.5321 | -2.7113 | 1.2587 | 0.9975 | 0.0075 | 17628.7017 | 1.0002 |
| eps_vals[58] | -0.0861 | -1.5472 | 1.5819 | 0.7378 | 0.0067 | 12090.6234 | 1.0001 |
| eps_vals[59] | 0.7339 | -0.2885 | 1.8802 | 0.5539 | 0.0069 | 6357.8591 | 1.0004 |
| eps_vals[60] | 0.5016 | -0.9013 | 2.2094 | 0.8138 | 0.0112 | 5300.5921 | 1.0004 |
| eps_vals[61] | 0.2429 | -0.5645 | 1.2879 | 0.4544 | 0.0044 | 10580.0889 | 1.0005 |
| eps_vals[62] | -1.0450 | -2.4208 | 0.0571 | 0.6328 | 0.0056 | 12907.9560 | 1.0003 |
| eps_vals[63] | -0.5601 | -1.7014 | 0.3535 | 0.5315 | 0.0057 | 8565.6554 | 1.0005 |
| eps_vals[64] | 0.5650 | -0.3726 | 1.8030 | 0.5552 | 0.0053 | 10861.3030 | 1.0001 |
| Rho_1[1,1] | 1.0000 | 1.0000 | 1.0000 | 0.0000 | NA | NA | NA |
| Rho_1[1,2] | 0.2119 | -0.3469 | 0.7041 | 0.2733 | 0.0022 | 15250.4089 | 1.0000 |
| Rho_1[1,3] | 0.0174 | -0.5738 | 0.5966 | 0.3072 | 0.0017 | 32316.7116 | 0.9999 |
| Rho_1[1,4] | 0.0185 | -0.5689 | 0.5936 | 0.3041 | 0.0017 | 30857.9819 | 0.9999 |
| Rho_1[1,5] | 0.0014 | -0.5700 | 0.5704 | 0.3006 | 0.0016 | 34190.6882 | 1.0000 |
| Rho_1[1,6] | -0.0014 | -0.5747 | 0.5731 | 0.2960 | 0.0016 | 34386.1720 | 0.9999 |
| Rho_1[1,7] | 0.0002 | -0.5698 | 0.5733 | 0.2993 | 0.0015 | 37641.9380 | 0.9999 |
| Rho_1[1,8] | 0.0031 | -0.5804 | 0.5828 | 0.3031 | 0.0017 | 31216.0178 | 0.9999 |
| Rho_1[2,1] | 0.2119 | -0.3469 | 0.7041 | 0.2733 | 0.0022 | 15250.4089 | 1.0000 |
| Rho_1[2,2] | 1.0000 | 1.0000 | 1.0000 | 0.0000 | 0.0000 | 19729.1392 | 0.9998 |
| Rho_1[2,3] | 0.0608 | -0.5194 | 0.6172 | 0.2976 | 0.0018 | 27307.8306 | 0.9999 |
| Rho_1[2,4] | 0.0368 | -0.5572 | 0.6298 | 0.3111 | 0.0018 | 29214.0034 | 0.9999 |
| Rho_1[2,5] | -0.0035 | -0.5711 | 0.5684 | 0.2983 | 0.0017 | 31350.7998 | 0.9999 |
| Rho_1[2,6] | -0.0025 | -0.5768 | 0.5793 | 0.3018 | 0.0018 | 29618.8241 | 1.0000 |
| Rho_1[2,7] | 0.0027 | -0.5757 | 0.5728 | 0.3022 | 0.0018 | 29609.0335 | 0.9999 |
| Rho_1[2,8] | -0.0012 | -0.5752 | 0.5794 | 0.3026 | 0.0018 | 28403.1789 | 0.9998 |
| Rho_1[3,1] | 0.0174 | -0.5738 | 0.5966 | 0.3072 | 0.0017 | 32316.7116 | 0.9999 |
| Rho_1[3,2] | 0.0608 | -0.5194 | 0.6172 | 0.2976 | 0.0018 | 27307.8306 | 0.9999 |
| Rho_1[3,3] | 1.0000 | 1.0000 | 1.0000 | 0.0000 | 0.0000 | 3387.6349 | 0.9998 |
| Rho_1[3,4] | -0.0024 | -0.5761 | 0.5865 | 0.3038 | 0.0020 | 22944.5136 | 1.0002 |
| Rho_1[3,5] | -0.0024 | -0.5864 | 0.5844 | 0.3059 | 0.0020 | 23283.2555 | 0.9999 |
| Rho_1[3,6] | -0.0003 | -0.5791 | 0.5703 | 0.3034 | 0.0021 | 21348.8008 | 1.0000 |
| Rho_1[3,7] | 0.0042 | -0.5768 | 0.5812 | 0.3025 | 0.0020 | 21981.0129 | 1.0003 |
| Rho_1[3,8] | -0.0002 | -0.5847 | 0.5693 | 0.3018 | 0.0020 | 22983.0582 | 1.0001 |
| Rho_1[4,1] | 0.0185 | -0.5689 | 0.5936 | 0.3041 | 0.0017 | 30857.9819 | 0.9999 |
| Rho_1[4,2] | 0.0368 | -0.5572 | 0.6298 | 0.3111 | 0.0018 | 29214.0034 | 0.9999 |
| Rho_1[4,3] | -0.0024 | -0.5761 | 0.5865 | 0.3038 | 0.0020 | 22944.5136 | 1.0002 |
| Rho_1[4,4] | 1.0000 | 1.0000 | 1.0000 | 0.0000 | 0.0000 | 15543.6396 | 0.9998 |
| Rho_1[4,5] | -0.0024 | -0.5740 | 0.5691 | 0.2992 | 0.0023 | 17521.2234 | 1.0002 |
| Rho_1[4,6] | -0.0018 | -0.5710 | 0.5735 | 0.3006 | 0.0022 | 19070.7086 | 1.0000 |
| Rho_1[4,7] | 0.0018 | -0.5773 | 0.5763 | 0.3004 | 0.0022 | 18216.2599 | 0.9999 |
| Rho_1[4,8] | 0.0022 | -0.5828 | 0.5841 | 0.3055 | 0.0022 | 18763.4488 | 0.9999 |
| Rho_1[5,1] | 0.0014 | -0.5700 | 0.5704 | 0.3006 | 0.0016 | 34190.6882 | 1.0000 |
| Rho_1[5,2] | -0.0035 | -0.5711 | 0.5684 | 0.2983 | 0.0017 | 31350.7998 | 0.9999 |
| Rho_1[5,3] | -0.0024 | -0.5864 | 0.5844 | 0.3059 | 0.0020 | 23283.2555 | 0.9999 |
| Rho_1[5,4] | -0.0024 | -0.5740 | 0.5691 | 0.2992 | 0.0023 | 17521.2234 | 1.0002 |
| Rho_1[5,5] | 1.0000 | 1.0000 | 1.0000 | 0.0000 | 0.0000 | 7099.2996 | 0.9998 |
| Rho_1[5,6] | 0.0399 | -0.5395 | 0.6024 | 0.3000 | 0.0025 | 14725.7797 | 0.9999 |
| Rho_1[5,7] | 0.0111 | -0.5666 | 0.5891 | 0.3032 | 0.0024 | 15810.0464 | 0.9999 |
| Rho_1[5,8] | 0.0566 | -0.5211 | 0.6128 | 0.2965 | 0.0024 | 14888.9920 | 1.0001 |
| Rho_1[6,1] | -0.0014 | -0.5747 | 0.5731 | 0.2960 | 0.0016 | 34386.1720 | 0.9999 |
| Rho_1[6,2] | -0.0025 | -0.5768 | 0.5793 | 0.3018 | 0.0018 | 29618.8241 | 1.0000 |
| Rho_1[6,3] | -0.0003 | -0.5791 | 0.5703 | 0.3034 | 0.0021 | 21348.8008 | 1.0000 |
| Rho_1[6,4] | -0.0018 | -0.5710 | 0.5735 | 0.3006 | 0.0022 | 19070.7086 | 1.0000 |
| Rho_1[6,5] | 0.0399 | -0.5395 | 0.6024 | 0.3000 | 0.0025 | 14725.7797 | 0.9999 |
| Rho_1[6,6] | 1.0000 | 1.0000 | 1.0000 | 0.0000 | 0.0000 | 12886.7499 | 0.9998 |
| Rho_1[6,7] | 0.0577 | -0.5186 | 0.6119 | 0.2967 | 0.0025 | 14255.4365 | 0.9999 |
| Rho_1[6,8] | 0.1203 | -0.4374 | 0.6466 | 0.2842 | 0.0024 | 13914.5622 | 0.9999 |
| Rho_1[7,1] | 0.0002 | -0.5698 | 0.5733 | 0.2993 | 0.0015 | 37641.9380 | 0.9999 |
| Rho_1[7,2] | 0.0027 | -0.5757 | 0.5728 | 0.3022 | 0.0018 | 29609.0335 | 0.9999 |
| Rho_1[7,3] | 0.0042 | -0.5768 | 0.5812 | 0.3025 | 0.0020 | 21981.0129 | 1.0003 |
| Rho_1[7,4] | 0.0018 | -0.5773 | 0.5763 | 0.3004 | 0.0022 | 18216.2599 | 0.9999 |
| Rho_1[7,5] | 0.0111 | -0.5666 | 0.5891 | 0.3032 | 0.0024 | 15810.0464 | 0.9999 |
| Rho_1[7,6] | 0.0577 | -0.5186 | 0.6119 | 0.2967 | 0.0025 | 14255.4365 | 0.9999 |
| Rho_1[7,7] | 1.0000 | 1.0000 | 1.0000 | 0.0000 | 0.0000 | 6152.0839 | 0.9998 |
| Rho_1[7,8] | 0.0981 | -0.4582 | 0.6257 | 0.2845 | 0.0026 | 12415.2501 | 1.0000 |
| Rho_1[8,1] | 0.0031 | -0.5804 | 0.5828 | 0.3031 | 0.0017 | 31216.0178 | 0.9999 |
| Rho_1[8,2] | -0.0012 | -0.5752 | 0.5794 | 0.3026 | 0.0018 | 28403.1789 | 0.9998 |
| Rho_1[8,3] | -0.0002 | -0.5847 | 0.5693 | 0.3018 | 0.0020 | 22983.0582 | 1.0001 |
| Rho_1[8,4] | 0.0022 | -0.5828 | 0.5841 | 0.3055 | 0.0022 | 18763.4488 | 0.9999 |
| Rho_1[8,5] | 0.0566 | -0.5211 | 0.6128 | 0.2965 | 0.0024 | 14888.9920 | 1.0001 |
| Rho_1[8,6] | 0.1203 | -0.4374 | 0.6466 | 0.2842 | 0.0024 | 13914.5622 | 0.9999 |
| Rho_1[8,7] | 0.0981 | -0.4582 | 0.6257 | 0.2845 | 0.0026 | 12415.2501 | 1.0000 |
| Rho_1[8,8] | 1.0000 | 1.0000 | 1.0000 | 0.0000 | 0.0000 | 15904.3819 | 0.9998 |
| Rho_2[1,1] | 1.0000 | 1.0000 | 1.0000 | 0.0000 | NA | NA | NA |
| Rho_2[1,2] | -0.0022 | -0.5687 | 0.5616 | 0.2954 | 0.0018 | 26966.1208 | 1.0000 |
| Rho_2[1,3] | 0.0019 | -0.5231 | 0.5372 | 0.2732 | 0.0017 | 24774.6207 | 0.9999 |
| Rho_2[1,4] | 0.0559 | -0.4998 | 0.5913 | 0.2834 | 0.0016 | 29895.2002 | 0.9998 |
| Rho_2[1,5] | 0.0016 | -0.5500 | 0.5510 | 0.2910 | 0.0016 | 32236.7545 | 0.9999 |
| Rho_2[1,6] | -0.0007 | -0.5572 | 0.5628 | 0.2908 | 0.0016 | 33546.1947 | 0.9998 |
| Rho_2[1,7] | -0.0004 | -0.5539 | 0.5465 | 0.2873 | 0.0016 | 31049.7265 | 1.0001 |
| Rho_2[1,8] | 0.0007 | -0.5495 | 0.5523 | 0.2891 | 0.0016 | 30901.0676 | 0.9999 |
| Rho_2[1,9] | 0.0015 | -0.5502 | 0.5565 | 0.2889 | 0.0016 | 34166.1939 | 1.0000 |
| Rho_2[2,1] | -0.0022 | -0.5687 | 0.5616 | 0.2954 | 0.0018 | 26966.1208 | 1.0000 |
| Rho_2[2,2] | 1.0000 | 1.0000 | 1.0000 | 0.0000 | 0.0000 | 20216.5474 | 0.9998 |
| Rho_2[2,3] | -0.0377 | -0.5914 | 0.5190 | 0.2891 | 0.0020 | 21302.1569 | 0.9999 |
| Rho_2[2,4] | 0.0192 | -0.5341 | 0.5691 | 0.2882 | 0.0018 | 26291.7515 | 1.0000 |
| Rho_2[2,5] | -0.0395 | -0.5782 | 0.5149 | 0.2863 | 0.0018 | 26235.8153 | 1.0000 |
| Rho_2[2,6] | 0.0010 | -0.5500 | 0.5566 | 0.2888 | 0.0018 | 26436.8461 | 1.0000 |
| Rho_2[2,7] | -0.0026 | -0.5533 | 0.5559 | 0.2911 | 0.0018 | 26902.3967 | 1.0001 |
| Rho_2[2,8] | -0.0005 | -0.5414 | 0.5490 | 0.2867 | 0.0018 | 24217.9597 | 1.0000 |
| Rho_2[2,9] | -0.0010 | -0.5537 | 0.5520 | 0.2880 | 0.0017 | 29252.8625 | 0.9998 |
| Rho_2[3,1] | 0.0019 | -0.5231 | 0.5372 | 0.2732 | 0.0017 | 24774.6207 | 0.9999 |
| Rho_2[3,2] | -0.0377 | -0.5914 | 0.5190 | 0.2891 | 0.0020 | 21302.1569 | 0.9999 |
| Rho_2[3,3] | 1.0000 | 1.0000 | 1.0000 | 0.0000 | 0.0000 | 8453.5079 | 0.9998 |
| Rho_2[3,4] | 0.0640 | -0.4845 | 0.5894 | 0.2796 | 0.0019 | 21060.2257 | 1.0000 |
| Rho_2[3,5] | 0.0031 | -0.5525 | 0.5571 | 0.2896 | 0.0019 | 23434.6297 | 1.0005 |
| Rho_2[3,6] | 0.0017 | -0.5485 | 0.5488 | 0.2858 | 0.0018 | 24544.2474 | 0.9999 |
| Rho_2[3,7] | -0.0002 | -0.5552 | 0.5532 | 0.2886 | 0.0018 | 24929.9686 | 1.0000 |
| Rho_2[3,8] | 0.0004 | -0.5595 | 0.5553 | 0.2905 | 0.0019 | 23910.5946 | 1.0001 |
| Rho_2[3,9] | 0.0005 | -0.5557 | 0.5500 | 0.2892 | 0.0019 | 23658.5272 | 1.0000 |
| Rho_2[4,1] | 0.0559 | -0.4998 | 0.5913 | 0.2834 | 0.0016 | 29895.2002 | 0.9998 |
| Rho_2[4,2] | 0.0192 | -0.5341 | 0.5691 | 0.2882 | 0.0018 | 26291.7515 | 1.0000 |
| Rho_2[4,3] | 0.0640 | -0.4845 | 0.5894 | 0.2796 | 0.0019 | 21060.2257 | 1.0000 |
| Rho_2[4,4] | 1.0000 | 1.0000 | 1.0000 | 0.0000 | 0.0000 | 15924.6714 | 0.9998 |
| Rho_2[4,5] | -0.0011 | -0.5520 | 0.5592 | 0.2899 | 0.0019 | 22381.9822 | 1.0000 |
| Rho_2[4,6] | 0.0024 | -0.5444 | 0.5511 | 0.2879 | 0.0020 | 20228.4005 | 1.0002 |
| Rho_2[4,7] | -0.0004 | -0.5513 | 0.5528 | 0.2886 | 0.0020 | 20618.4673 | 1.0003 |
| Rho_2[4,8] | 0.0004 | -0.5559 | 0.5509 | 0.2885 | 0.0020 | 21642.6093 | 0.9999 |
| Rho_2[4,9] | 0.0007 | -0.5604 | 0.5499 | 0.2874 | 0.0020 | 20540.7334 | 0.9999 |
| Rho_2[5,1] | 0.0016 | -0.5500 | 0.5510 | 0.2910 | 0.0016 | 32236.7545 | 0.9999 |
| Rho_2[5,2] | -0.0395 | -0.5782 | 0.5149 | 0.2863 | 0.0018 | 26235.8153 | 1.0000 |
| Rho_2[5,3] | 0.0031 | -0.5525 | 0.5571 | 0.2896 | 0.0019 | 23434.6297 | 1.0005 |
| Rho_2[5,4] | -0.0011 | -0.5520 | 0.5592 | 0.2899 | 0.0019 | 22381.9822 | 1.0000 |
| Rho_2[5,5] | 1.0000 | 1.0000 | 1.0000 | 0.0000 | 0.0000 | 18581.4926 | 0.9998 |
| Rho_2[5,6] | 0.0540 | -0.4966 | 0.5944 | 0.2850 | 0.0022 | 17354.7732 | 1.0000 |
| Rho_2[5,7] | 0.0400 | -0.5171 | 0.5824 | 0.2880 | 0.0022 | 17340.7946 | 1.0003 |
| Rho_2[5,8] | 0.0804 | -0.4694 | 0.6041 | 0.2787 | 0.0022 | 15642.8830 | 1.0001 |
| Rho_2[5,9] | 0.0148 | -0.5377 | 0.5677 | 0.2878 | 0.0022 | 17667.3329 | 1.0001 |
| Rho_2[6,1] | -0.0007 | -0.5572 | 0.5628 | 0.2908 | 0.0016 | 33546.1947 | 0.9998 |
| Rho_2[6,2] | 0.0010 | -0.5500 | 0.5566 | 0.2888 | 0.0018 | 26436.8461 | 1.0000 |
| Rho_2[6,3] | 0.0017 | -0.5485 | 0.5488 | 0.2858 | 0.0018 | 24544.2474 | 0.9999 |
| Rho_2[6,4] | 0.0024 | -0.5444 | 0.5511 | 0.2879 | 0.0020 | 20228.4005 | 1.0002 |
| Rho_2[6,5] | 0.0540 | -0.4966 | 0.5944 | 0.2850 | 0.0022 | 17354.7732 | 1.0000 |
| Rho_2[6,6] | 1.0000 | 1.0000 | 1.0000 | 0.0000 | 0.0000 | 13796.0098 | 0.9998 |
| Rho_2[6,7] | 0.0255 | -0.5271 | 0.5712 | 0.2854 | 0.0023 | 15364.1960 | 1.0000 |
| Rho_2[6,8] | 0.0746 | -0.4888 | 0.6113 | 0.2879 | 0.0025 | 13585.7077 | 1.0001 |
| Rho_2[6,9] | 0.0132 | -0.5463 | 0.5622 | 0.2885 | 0.0023 | 15667.0719 | 1.0002 |
| Rho_2[7,1] | -0.0004 | -0.5539 | 0.5465 | 0.2873 | 0.0016 | 31049.7265 | 1.0001 |
| Rho_2[7,2] | -0.0026 | -0.5533 | 0.5559 | 0.2911 | 0.0018 | 26902.3967 | 1.0001 |
| Rho_2[7,3] | -0.0002 | -0.5552 | 0.5532 | 0.2886 | 0.0018 | 24929.9686 | 1.0000 |
| Rho_2[7,4] | -0.0004 | -0.5513 | 0.5528 | 0.2886 | 0.0020 | 20618.4673 | 1.0003 |
| Rho_2[7,5] | 0.0400 | -0.5171 | 0.5824 | 0.2880 | 0.0022 | 17340.7946 | 1.0003 |
| Rho_2[7,6] | 0.0255 | -0.5271 | 0.5712 | 0.2854 | 0.0023 | 15364.1960 | 1.0000 |
| Rho_2[7,7] | 1.0000 | 1.0000 | 1.0000 | 0.0000 | 0.0000 | 14281.7564 | 0.9998 |
| Rho_2[7,8] | 0.0726 | -0.4724 | 0.5994 | 0.2785 | 0.0025 | 12287.5850 | 0.9999 |
| Rho_2[7,9] | 0.0314 | -0.5292 | 0.5719 | 0.2877 | 0.0025 | 13510.9733 | 1.0001 |
| Rho_2[8,1] | 0.0007 | -0.5495 | 0.5523 | 0.2891 | 0.0016 | 30901.0676 | 0.9999 |
| Rho_2[8,2] | -0.0005 | -0.5414 | 0.5490 | 0.2867 | 0.0018 | 24217.9597 | 1.0000 |
| Rho_2[8,3] | 0.0004 | -0.5595 | 0.5553 | 0.2905 | 0.0019 | 23910.5946 | 1.0001 |
| Rho_2[8,4] | 0.0004 | -0.5559 | 0.5509 | 0.2885 | 0.0020 | 21642.6093 | 0.9999 |
| Rho_2[8,5] | 0.0804 | -0.4694 | 0.6041 | 0.2787 | 0.0022 | 15642.8830 | 1.0001 |
| Rho_2[8,6] | 0.0746 | -0.4888 | 0.6113 | 0.2879 | 0.0025 | 13585.7077 | 1.0001 |
| Rho_2[8,7] | 0.0726 | -0.4724 | 0.5994 | 0.2785 | 0.0025 | 12287.5850 | 0.9999 |
| Rho_2[8,8] | 1.0000 | 1.0000 | 1.0000 | 0.0000 | 0.0000 | 8513.6742 | 0.9998 |
| Rho_2[8,9] | 0.0639 | -0.4825 | 0.5868 | 0.2790 | 0.0025 | 12484.8101 | 1.0002 |
| Rho_2[9,1] | 0.0015 | -0.5502 | 0.5565 | 0.2889 | 0.0016 | 34166.1939 | 1.0000 |
| Rho_2[9,2] | -0.0010 | -0.5537 | 0.5520 | 0.2880 | 0.0017 | 29252.8625 | 0.9998 |
| Rho_2[9,3] | 0.0005 | -0.5557 | 0.5500 | 0.2892 | 0.0019 | 23658.5272 | 1.0000 |
| Rho_2[9,4] | 0.0007 | -0.5604 | 0.5499 | 0.2874 | 0.0020 | 20540.7334 | 0.9999 |
| Rho_2[9,5] | 0.0148 | -0.5377 | 0.5677 | 0.2878 | 0.0022 | 17667.3329 | 1.0001 |
| Rho_2[9,6] | 0.0132 | -0.5463 | 0.5622 | 0.2885 | 0.0023 | 15667.0719 | 1.0002 |
| Rho_2[9,7] | 0.0314 | -0.5292 | 0.5719 | 0.2877 | 0.0025 | 13510.9733 | 1.0001 |
| Rho_2[9,8] | 0.0639 | -0.4825 | 0.5868 | 0.2790 | 0.0025 | 12484.8101 | 1.0002 |
| Rho_2[9,9] | 1.0000 | 1.0000 | 1.0000 | 0.0000 | 0.0000 | 19675.7397 | 0.9998 |
| p[1] | 0.1232 | 0.0405 | 0.2517 | 0.0554 | 0.0007 | 6406.7336 | 1.0003 |
| p[2] | 0.0396 | 0.0058 | 0.1126 | 0.0296 | 0.0004 | 5465.5078 | 1.0002 |
| p[3] | 0.0701 | 0.0251 | 0.1307 | 0.0267 | 0.0003 | 10957.2617 | 1.0004 |
| p[4] | 0.1079 | 0.0392 | 0.2444 | 0.0532 | 0.0005 | 11911.6956 | 1.0004 |
| p[5] | 0.1149 | 0.0452 | 0.2588 | 0.0561 | 0.0006 | 7538.2674 | 1.0008 |
| p[6] | 0.0788 | 0.0041 | 0.3119 | 0.0853 | 0.0007 | 15730.3883 | 1.0002 |
| p[7] | 0.1938 | 0.0378 | 0.4692 | 0.1058 | 0.0009 | 12649.8071 | 1.0001 |
| p[8] | 0.1780 | 0.0181 | 0.5715 | 0.1417 | 0.0014 | 10668.6409 | 1.0004 |
| p[9] | 0.0776 | 0.0083 | 0.2326 | 0.0609 | 0.0005 | 12707.7669 | 1.0003 |
| p[10] | 0.0597 | 0.0064 | 0.2064 | 0.0560 | 0.0005 | 13173.7419 | 0.9999 |
| p[11] | 0.1365 | 0.0423 | 0.2757 | 0.0594 | 0.0007 | 7841.1251 | 0.9999 |
| p[12] | 0.0440 | 0.0077 | 0.1415 | 0.0354 | 0.0004 | 6443.8091 | 1.0006 |
| p[13] | 0.0818 | 0.0245 | 0.1812 | 0.0406 | 0.0005 | 7664.7629 | 1.0005 |
| p[14] | 0.1228 | 0.0330 | 0.2711 | 0.0624 | 0.0006 | 10597.2658 | 1.0001 |
| p[15] | 0.1244 | 0.0395 | 0.2060 | 0.0420 | 0.0004 | 10345.7691 | 0.9999 |
| p[16] | 0.0911 | 0.0042 | 0.3674 | 0.0988 | 0.0008 | 14617.0520 | 1.0003 |
| p[17] | 0.2193 | 0.0366 | 0.5466 | 0.1309 | 0.0013 | 9661.9579 | 1.0002 |
| p[18] | 0.1881 | 0.0226 | 0.5690 | 0.1355 | 0.0012 | 12264.6645 | 1.0001 |
| p[19] | 0.0877 | 0.0083 | 0.2414 | 0.0661 | 0.0006 | 10622.4509 | 1.0002 |
| p[20] | 0.0664 | 0.0071 | 0.2205 | 0.0594 | 0.0005 | 13077.3964 | 1.0001 |

Table S11: Parameter estimates for 2009-type severity ($\beta_{k\mathcal{l}}$: beta_sero_prior, $\beta_{j}$: beta_reg, $\alpha$: intercept, $\sigma_{jk\mathcal{l}}$: sigma, $\epsilon_{ijk\mathcal{l}}$: eps_vals, $P_{j}$: Rho_1 and Rho_2, $p_{jk\mathcal{l}}$: p)

## Trace Plots and Parameter Estimates (Hospitalisation)

Figure S8: Trace plot for random effects $\epsilon_{ijk\mathcal{l}}$ for hospitalisation

Figure S9: Trace plot for prior exposure-serotype effects $\beta_{k\mathcal{l}}$, region effect $\beta_{j}$, intercept $\alpha$, and standard deviation of random effects $\sigma_{jk\mathcal{l}}$ for hospitalisation.

Figure S10: Trace plot for region-specific correlation matrices $P_{j}$ for hospitalisation (including diagonal elements that are always 1 by definition).

| Parameter | Mean | 2.50% | 97.50% | SD | SE_Mean | ESS | Rhat |
| --- | --- | --- | --- | --- | --- | --- | --- |
| beta_sero_prior[1] | -0.0067 | -1.7108 | 1.5771 | 0.8415 | 0.0101 | 6908.1909 | 1.0004 |
| beta_sero_prior[2] | 0.5899 | -0.8927 | 2.0503 | 0.7478 | 0.0082 | 8371.1995 | 1.0001 |
| beta_sero_prior[3] | 0.8990 | -0.5360 | 2.3165 | 0.7209 | 0.0079 | 8297.6689 | 1.0004 |
| beta_sero_prior[4] | 0.1547 | -1.7541 | 1.9413 | 0.9335 | 0.0087 | 11459.1503 | 1.0001 |
| beta_sero_prior[5] | -0.9154 | -3.0153 | 1.3706 | 1.0968 | 0.0095 | 13397.3228 | 1.0001 |
| beta_sero_prior[6] | -1.3325 | -3.9389 | 1.2478 | 1.3087 | 0.0098 | 17776.5815 | 1.0002 |
| beta_sero_prior[7] | -0.2508 | -3.1872 | 2.5433 | 1.4501 | 0.0101 | 20565.8544 | 1.0000 |
| beta_sero_prior[8] | -0.3903 | -2.8955 | 2.2161 | 1.2856 | 0.0095 | 18382.8891 | 0.9999 |
| beta_reg | -0.6065 | -1.7770 | 0.5114 | 0.5836 | 0.0068 | 7440.5996 | 0.9999 |
| intercept | -1.4812 | -2.7790 | -0.1706 | 0.6569 | 0.0072 | 8281.1516 | 1.0003 |
| sigma[1] | 1.9949 | 0.8469 | 4.1364 | 0.8660 | 0.0090 | 9355.0124 | 1.0002 |
| sigma[2] | 1.6122 | 0.8542 | 2.9639 | 0.5507 | 0.0051 | 11435.7793 | 1.0000 |
| sigma[3] | 1.5946 | 0.6648 | 3.4656 | 0.7305 | 0.0061 | 14209.2009 | 1.0001 |
| sigma[4] | 1.7392 | 0.6063 | 4.0418 | 0.9022 | 0.0065 | 19173.2659 | 1.0001 |
| sigma[5] | 1.7605 | 0.8308 | 3.4900 | 0.6863 | 0.0072 | 9041.7319 | 1.0002 |
| sigma[6] | 1.3494 | 0.6715 | 2.6380 | 0.5138 | 0.0053 | 9404.0445 | 1.0004 |
| sigma[7] | 0.7645 | 0.3081 | 1.7780 | 0.3925 | 0.0038 | 10852.6855 | 1.0001 |
| sigma[8] | 1.9563 | 0.4977 | 4.3057 | 0.9938 | 0.0074 | 18246.3124 | 1.0002 |
| sigma[9] | 1.3285 | 0.0590 | 3.7989 | 1.0086 | 0.0085 | 14156.0220 | 0.9999 |
| sigma[10] | 1.5876 | 0.0678 | 4.4445 | 1.1893 | 0.0090 | 17511.7923 | 1.0000 |
| sigma[11] | 2.7778 | 1.0570 | 5.3040 | 1.1117 | 0.0066 | 28023.4989 | 1.0000 |
| sigma[12] | 2.1653 | 0.5580 | 4.5931 | 1.0392 | 0.0073 | 20398.0843 | 1.0000 |
| sigma[13] | 1.9063 | 0.4832 | 4.2744 | 0.9790 | 0.0068 | 20673.4118 | 1.0001 |
| eps_vals[1] | -2.2272 | -6.2693 | 0.1584 | 1.6748 | 0.0149 | 12663.3565 | 0.9999 |
| eps_vals[2] | -1.3497 | -4.0868 | 0.4629 | 1.1687 | 0.0083 | 19998.8539 | 1.0000 |
| eps_vals[3] | -0.7631 | -1.5585 | -0.0810 | 0.3666 | 0.0032 | 13220.1760 | 1.0001 |
| eps_vals[4] | 1.2390 | -0.0245 | 2.7023 | 0.6969 | 0.0084 | 6955.1246 | 1.0001 |
| eps_vals[5] | 1.0653 | 0.0821 | 2.1194 | 0.5120 | 0.0048 | 11525.3043 | 1.0001 |
| eps_vals[6] | -0.0348 | -0.8146 | 0.6679 | 0.3635 | 0.0031 | 13583.8685 | 1.0001 |
| eps_vals[7] | -1.7378 | -5.7846 | 0.6083 | 1.6363 | 0.0149 | 12108.3815 | 1.0001 |
| eps_vals[8] | -0.3857 | -2.1091 | 1.2524 | 0.8535 | 0.0071 | 14495.8967 | 0.9999 |
| eps_vals[9] | 0.0578 | -1.3497 | 1.3254 | 0.6690 | 0.0084 | 6268.4651 | 1.0003 |
| eps_vals[10] | 0.4233 | -0.6127 | 1.5935 | 0.5609 | 0.0065 | 7491.2264 | 0.9999 |
| eps_vals[11] | -0.4073 | -1.5567 | 0.8730 | 0.6187 | 0.0070 | 7868.3090 | 1.0000 |
| eps_vals[12] | -0.4366 | -1.9780 | 1.4065 | 0.8355 | 0.0075 | 12570.6853 | 0.9999 |
| eps_vals[13] | -0.0377 | -1.4754 | 1.2534 | 0.6828 | 0.0085 | 6428.1689 | 1.0003 |
| eps_vals[14] | 0.3437 | -0.8009 | 1.5706 | 0.6063 | 0.0067 | 8225.4320 | 0.9999 |
| eps_vals[15] | -1.2888 | -2.5256 | 0.1129 | 0.6632 | 0.0080 | 6920.3154 | 1.0002 |
| eps_vals[16] | -1.5238 | -2.6198 | -0.4747 | 0.5437 | 0.0051 | 11551.0812 | 1.0001 |
| eps_vals[17] | -2.5863 | -6.7352 | -0.2110 | 1.6658 | 0.0151 | 12096.4728 | 1.0001 |
| eps_vals[18] | 1.0749 | 0.1684 | 2.0304 | 0.4679 | 0.0046 | 10140.4400 | 1.0001 |
| eps_vals[19] | -1.3927 | -3.0336 | 0.0481 | 0.7745 | 0.0089 | 7511.4137 | 1.0002 |
| eps_vals[20] | -2.1554 | -3.4792 | -0.8994 | 0.6545 | 0.0066 | 9732.5985 | 0.9999 |
| eps_vals[21] | 2.1449 | -0.7034 | 5.1790 | 1.4905 | 0.0110 | 18350.8399 | 0.9999 |
| eps_vals[22] | 1.5736 | 0.1713 | 2.8375 | 0.6678 | 0.0084 | 6272.8030 | 1.0003 |
| eps_vals[23] | 1.5065 | 0.4681 | 2.6702 | 0.5597 | 0.0065 | 7470.5743 | 0.9999 |
| eps_vals[24] | 1.6289 | 0.4873 | 2.9070 | 0.6178 | 0.0070 | 7900.2902 | 1.0000 |
| eps_vals[25] | 1.2760 | -0.2707 | 3.1252 | 0.8352 | 0.0075 | 12541.9192 | 0.9999 |
| eps_vals[26] | 1.5149 | 0.4468 | 2.6908 | 0.5681 | 0.0065 | 7550.4318 | 0.9999 |
| eps_vals[27] | 0.9352 | -0.2246 | 2.2324 | 0.6274 | 0.0070 | 8103.3936 | 1.0001 |
| eps_vals[28] | 2.0322 | 0.8222 | 3.4342 | 0.6663 | 0.0083 | 6518.5956 | 1.0003 |
| eps_vals[29] | 0.5567 | -0.3454 | 1.5078 | 0.4614 | 0.0046 | 9854.1184 | 1.0002 |
| eps_vals[30] | 0.3830 | -0.4186 | 1.1765 | 0.3954 | 0.0032 | 14892.1814 | 1.0003 |
| eps_vals[31] | 0.1819 | -1.9569 | 2.2736 | 0.9807 | 0.0075 | 17026.7409 | 1.0004 |
| eps_vals[32] | 0.0847 | -2.5778 | 2.6161 | 1.2104 | 0.0089 | 18669.1294 | 1.0001 |
| eps_vals[33] | 1.2034 | -1.3842 | 4.0091 | 1.3389 | 0.0100 | 17938.6117 | 1.0002 |
| eps_vals[34] | 1.1148 | -0.4032 | 2.7714 | 0.8123 | 0.0079 | 10503.4279 | 1.0004 |
| eps_vals[35] | 0.3610 | -0.9558 | 1.8363 | 0.7107 | 0.0085 | 7026.8979 | 1.0004 |
| eps_vals[36] | -0.6713 | -1.6697 | 0.3302 | 0.5022 | 0.0048 | 10837.1984 | 1.0004 |
| eps_vals[37] | -0.1603 | -1.0006 | 0.6041 | 0.3980 | 0.0033 | 14411.5557 | 1.0001 |
| eps_vals[38] | -1.5782 | -5.6016 | 0.7317 | 1.6423 | 0.0108 | 22970.0520 | 1.0001 |
| eps_vals[39] | 1.5546 | 0.3659 | 2.9343 | 0.6560 | 0.0082 | 6380.1473 | 1.0003 |
| eps_vals[40] | 0.5104 | -0.4352 | 1.4981 | 0.4828 | 0.0047 | 10426.0647 | 1.0002 |
| eps_vals[41] | 0.5483 | -0.2404 | 1.3684 | 0.3986 | 0.0032 | 15105.3757 | 1.0003 |
| eps_vals[42] | 1.4536 | -0.1101 | 3.2681 | 0.8707 | 0.0072 | 14622.9419 | 0.9999 |
| eps_vals[43] | -0.4992 | -2.9140 | 1.5773 | 1.1161 | 0.0079 | 20128.8328 | 0.9999 |
| eps_vals[44] | -0.6702 | -3.2386 | 1.1178 | 1.0756 | 0.0088 | 14892.8926 | 1.0001 |
| eps_vals[45] | -1.1721 | -6.2060 | 1.2779 | 1.9135 | 0.0141 | 18483.5118 | 1.0001 |
| eps_vals[46] | -2.9999 | -8.3661 | 0.4833 | 2.2519 | 0.0166 | 18414.8941 | 0.9998 |
| eps_vals[47] | -1.7972 | -5.0207 | 0.7580 | 1.4523 | 0.0109 | 17667.5960 | 0.9999 |
| eps_vals[48] | -1.5216 | -3.8945 | 0.2389 | 1.0655 | 0.0092 | 13286.5333 | 0.9999 |
| Rho_1[1,1] | 1.0000 | 1.0000 | 1.0000 | 0.0000 | NA | NA | NA |
| Rho_1[1,2] | 0.3250 | -0.3717 | 0.8534 | 0.3278 | 0.0027 | 14468.5460 | 1.0000 |
| Rho_1[1,3] | 0.1985 | -0.5052 | 0.8027 | 0.3489 | 0.0023 | 23145.6186 | 0.9999 |
| Rho_1[1,4] | 0.1327 | -0.5880 | 0.7839 | 0.3640 | 0.0022 | 27755.9042 | 0.9999 |
| Rho_1[2,1] | 0.3250 | -0.3717 | 0.8534 | 0.3278 | 0.0027 | 14468.5460 | 1.0000 |
| Rho_1[2,2] | 1.0000 | 1.0000 | 1.0000 | 0.0000 | 0.0000 | 19883.0809 | 0.9998 |
| Rho_1[2,3] | 0.2492 | -0.4667 | 0.8325 | 0.3485 | 0.0025 | 19525.9440 | 0.9999 |
| Rho_1[2,4] | 0.1222 | -0.5775 | 0.7642 | 0.3586 | 0.0022 | 26117.3835 | 0.9999 |
| Rho_1[3,1] | 0.1985 | -0.5052 | 0.8027 | 0.3489 | 0.0023 | 23145.6186 | 0.9999 |
| Rho_1[3,2] | 0.2492 | -0.4667 | 0.8325 | 0.3485 | 0.0025 | 19525.9440 | 0.9999 |
| Rho_1[3,3] | 1.0000 | 1.0000 | 1.0000 | 0.0000 | 0.0000 | 7129.7514 | 0.9998 |
| Rho_1[3,4] | 0.1992 | -0.4908 | 0.7987 | 0.3455 | 0.0026 | 17521.2198 | 1.0001 |
| Rho_1[4,1] | 0.1327 | -0.5880 | 0.7839 | 0.3640 | 0.0022 | 27755.9042 | 0.9999 |
| Rho_1[4,2] | 0.1222 | -0.5775 | 0.7642 | 0.3586 | 0.0022 | 26117.3835 | 0.9999 |
| Rho_1[4,3] | 0.1992 | -0.4908 | 0.7987 | 0.3455 | 0.0026 | 17521.2198 | 1.0001 |
| Rho_1[4,4] | 1.0000 | 1.0000 | 1.0000 | 0.0000 | 0.0000 | 4047.3901 | 0.9998 |
| Rho_2[1,1] | 1.0000 | 1.0000 | 1.0000 | 0.0000 | NA | NA | NA |
| Rho_2[1,2] | 0.1985 | -0.3404 | 0.6806 | 0.2660 | 0.0021 | 16636.8468 | 0.9999 |
| Rho_2[1,3] | 0.1241 | -0.4632 | 0.6564 | 0.2928 | 0.0018 | 25904.5203 | 1.0001 |
| Rho_2[1,4] | 0.0669 | -0.4702 | 0.5747 | 0.2728 | 0.0014 | 36182.0362 | 1.0000 |
| Rho_2[1,5] | -0.0023 | -0.5487 | 0.5500 | 0.2894 | 0.0015 | 38961.3983 | 0.9999 |
| Rho_2[1,6] | -0.0005 | -0.5490 | 0.5525 | 0.2864 | 0.0015 | 36801.2999 | 0.9999 |
| Rho_2[1,7] | -0.0027 | -0.5560 | 0.5555 | 0.2895 | 0.0015 | 35100.9284 | 1.0000 |
| Rho_2[1,8] | -0.0001 | -0.5498 | 0.5427 | 0.2874 | 0.0015 | 35254.4201 | 0.9999 |
| Rho_2[1,9] | -0.0004 | -0.5561 | 0.5576 | 0.2882 | 0.0015 | 36863.8989 | 0.9999 |
| Rho_2[2,1] | 0.1985 | -0.3404 | 0.6806 | 0.2660 | 0.0021 | 16636.8468 | 0.9999 |
| Rho_2[2,2] | 1.0000 | 1.0000 | 1.0000 | 0.0000 | 0.0000 | 19966.1979 | 0.9998 |
| Rho_2[2,3] | 0.0551 | -0.5107 | 0.5974 | 0.2912 | 0.0019 | 23526.6203 | 0.9999 |
| Rho_2[2,4] | 0.0823 | -0.4531 | 0.6060 | 0.2752 | 0.0015 | 31652.0409 | 0.9999 |
| Rho_2[2,5] | -0.0009 | -0.5554 | 0.5516 | 0.2893 | 0.0017 | 29642.3350 | 0.9999 |
| Rho_2[2,6] | 0.0010 | -0.5483 | 0.5476 | 0.2853 | 0.0016 | 32732.9962 | 1.0000 |
| Rho_2[2,7] | -0.0021 | -0.5622 | 0.5497 | 0.2903 | 0.0016 | 31287.8150 | 0.9999 |
| Rho_2[2,8] | -0.0022 | -0.5499 | 0.5551 | 0.2879 | 0.0016 | 31842.4294 | 0.9999 |
| Rho_2[2,9] | -0.0001 | -0.5511 | 0.5529 | 0.2868 | 0.0017 | 28552.4157 | 0.9999 |
| Rho_2[3,1] | 0.1241 | -0.4632 | 0.6564 | 0.2928 | 0.0018 | 25904.5203 | 1.0001 |
| Rho_2[3,2] | 0.0551 | -0.5107 | 0.5974 | 0.2912 | 0.0019 | 23526.6203 | 0.9999 |
| Rho_2[3,3] | 1.0000 | 1.0000 | 1.0000 | 0.0000 | 0.0000 | 14520.7269 | 0.9998 |
| Rho_2[3,4] | 0.0877 | -0.4503 | 0.6020 | 0.2737 | 0.0018 | 23585.2077 | 1.0000 |
| Rho_2[3,5] | -0.0009 | -0.5568 | 0.5496 | 0.2887 | 0.0017 | 28092.6184 | 1.0000 |
| Rho_2[3,6] | -0.0007 | -0.5561 | 0.5525 | 0.2894 | 0.0018 | 24846.9084 | 1.0001 |
| Rho_2[3,7] | 0.0001 | -0.5540 | 0.5625 | 0.2912 | 0.0018 | 26473.0427 | 0.9998 |
| Rho_2[3,8] | -0.0013 | -0.5624 | 0.5479 | 0.2901 | 0.0018 | 25469.9805 | 0.9998 |
| Rho_2[3,9] | 0.0010 | -0.5528 | 0.5557 | 0.2892 | 0.0018 | 24556.6880 | 0.9999 |
| Rho_2[4,1] | 0.0669 | -0.4702 | 0.5747 | 0.2728 | 0.0014 | 36182.0362 | 1.0000 |
| Rho_2[4,2] | 0.0823 | -0.4531 | 0.6060 | 0.2752 | 0.0015 | 31652.0409 | 0.9999 |
| Rho_2[4,3] | 0.0877 | -0.4503 | 0.6020 | 0.2737 | 0.0018 | 23585.2077 | 1.0000 |
| Rho_2[4,4] | 1.0000 | 1.0000 | 1.0000 | 0.0000 | 0.0000 | 17447.1239 | 0.9998 |
| Rho_2[4,5] | -0.0041 | -0.5539 | 0.5526 | 0.2893 | 0.0020 | 21751.3189 | 1.0000 |
| Rho_2[4,6] | 0.0016 | -0.5554 | 0.5554 | 0.2893 | 0.0020 | 20649.3869 | 0.9999 |
| Rho_2[4,7] | 0.0015 | -0.5509 | 0.5479 | 0.2879 | 0.0019 | 23043.8167 | 0.9999 |
| Rho_2[4,8] | 0.0000 | -0.5583 | 0.5519 | 0.2879 | 0.0021 | 19322.9708 | 0.9999 |
| Rho_2[4,9] | 0.0006 | -0.5477 | 0.5555 | 0.2870 | 0.0020 | 20004.8761 | 1.0000 |
| Rho_2[5,1] | -0.0023 | -0.5487 | 0.5500 | 0.2894 | 0.0015 | 38961.3983 | 0.9999 |
| Rho_2[5,2] | -0.0009 | -0.5554 | 0.5516 | 0.2893 | 0.0017 | 29642.3350 | 0.9999 |
| Rho_2[5,3] | -0.0009 | -0.5568 | 0.5496 | 0.2887 | 0.0017 | 28092.6184 | 1.0000 |
| Rho_2[5,4] | -0.0041 | -0.5539 | 0.5526 | 0.2893 | 0.0020 | 21751.3189 | 1.0000 |
| Rho_2[5,5] | 1.0000 | 1.0000 | 1.0000 | 0.0000 | 0.0000 | 5625.0943 | 0.9998 |
| Rho_2[5,6] | 0.0267 | -0.5282 | 0.5752 | 0.2897 | 0.0022 | 17903.0037 | 1.0000 |
| Rho_2[5,7] | 0.0423 | -0.5089 | 0.5742 | 0.2843 | 0.0022 | 16514.2922 | 0.9999 |
| Rho_2[5,8] | 0.0503 | -0.5102 | 0.5794 | 0.2848 | 0.0021 | 18062.2177 | 0.9999 |
| Rho_2[5,9] | 0.0511 | -0.4954 | 0.5787 | 0.2816 | 0.0022 | 15931.5885 | 0.9999 |
| Rho_2[6,1] | -0.0005 | -0.5490 | 0.5525 | 0.2864 | 0.0015 | 36801.2999 | 0.9999 |
| Rho_2[6,2] | 0.0010 | -0.5483 | 0.5476 | 0.2853 | 0.0016 | 32732.9962 | 1.0000 |
| Rho_2[6,3] | -0.0007 | -0.5561 | 0.5525 | 0.2894 | 0.0018 | 24846.9084 | 1.0001 |
| Rho_2[6,4] | 0.0016 | -0.5554 | 0.5554 | 0.2893 | 0.0020 | 20649.3869 | 0.9999 |
| Rho_2[6,5] | 0.0267 | -0.5282 | 0.5752 | 0.2897 | 0.0022 | 17903.0037 | 1.0000 |
| Rho_2[6,6] | 1.0000 | 1.0000 | 1.0000 | 0.0000 | 0.0000 | 16402.7170 | 0.9998 |
| Rho_2[6,7] | 0.0486 | -0.5186 | 0.5815 | 0.2848 | 0.0024 | 14557.1737 | 1.0003 |
| Rho_2[6,8] | 0.0450 | -0.5018 | 0.5792 | 0.2831 | 0.0023 | 14560.8456 | 1.0003 |
| Rho_2[6,9] | 0.0506 | -0.5088 | 0.5862 | 0.2860 | 0.0024 | 14604.9643 | 1.0001 |
| Rho_2[7,1] | -0.0027 | -0.5560 | 0.5555 | 0.2895 | 0.0015 | 35100.9284 | 1.0000 |
| Rho_2[7,2] | -0.0021 | -0.5622 | 0.5497 | 0.2903 | 0.0016 | 31287.8150 | 0.9999 |
| Rho_2[7,3] | 0.0001 | -0.5540 | 0.5625 | 0.2912 | 0.0018 | 26473.0427 | 0.9998 |
| Rho_2[7,4] | 0.0015 | -0.5509 | 0.5479 | 0.2879 | 0.0019 | 23043.8167 | 0.9999 |
| Rho_2[7,5] | 0.0423 | -0.5089 | 0.5742 | 0.2843 | 0.0022 | 16514.2922 | 0.9999 |
| Rho_2[7,6] | 0.0486 | -0.5186 | 0.5815 | 0.2848 | 0.0024 | 14557.1737 | 1.0003 |
| Rho_2[7,7] | 1.0000 | 1.0000 | 1.0000 | 0.0000 | 0.0000 | 10387.7650 | 0.9998 |
| Rho_2[7,8] | 0.0861 | -0.4552 | 0.6032 | 0.2781 | 0.0024 | 13944.7046 | 1.0001 |
| Rho_2[7,9] | 0.0829 | -0.4678 | 0.6101 | 0.2808 | 0.0024 | 13431.4673 | 1.0003 |
| Rho_2[8,1] | -0.0001 | -0.5498 | 0.5427 | 0.2874 | 0.0015 | 35254.4201 | 0.9999 |
| Rho_2[8,2] | -0.0022 | -0.5499 | 0.5551 | 0.2879 | 0.0016 | 31842.4294 | 0.9999 |
| Rho_2[8,3] | -0.0013 | -0.5624 | 0.5479 | 0.2901 | 0.0018 | 25469.9805 | 0.9998 |
| Rho_2[8,4] | 0.0000 | -0.5583 | 0.5519 | 0.2879 | 0.0021 | 19322.9708 | 0.9999 |
| Rho_2[8,5] | 0.0503 | -0.5102 | 0.5794 | 0.2848 | 0.0021 | 18062.2177 | 0.9999 |
| Rho_2[8,6] | 0.0450 | -0.5018 | 0.5792 | 0.2831 | 0.0023 | 14560.8456 | 1.0003 |
| Rho_2[8,7] | 0.0861 | -0.4552 | 0.6032 | 0.2781 | 0.0024 | 13944.7046 | 1.0001 |
| Rho_2[8,8] | 1.0000 | 1.0000 | 1.0000 | 0.0000 | 0.0000 | 12681.5899 | 0.9998 |
| Rho_2[8,9] | 0.1070 | -0.4400 | 0.6184 | 0.2746 | 0.0024 | 13438.9093 | 1.0002 |
| Rho_2[9,1] | -0.0004 | -0.5561 | 0.5576 | 0.2882 | 0.0015 | 36863.8989 | 0.9999 |
| Rho_2[9,2] | -0.0001 | -0.5511 | 0.5529 | 0.2868 | 0.0017 | 28552.4157 | 0.9999 |
| Rho_2[9,3] | 0.0010 | -0.5528 | 0.5557 | 0.2892 | 0.0018 | 24556.6880 | 0.9999 |
| Rho_2[9,4] | 0.0006 | -0.5477 | 0.5555 | 0.2870 | 0.0020 | 20004.8761 | 1.0000 |
| Rho_2[9,5] | 0.0511 | -0.4954 | 0.5787 | 0.2816 | 0.0022 | 15931.5885 | 0.9999 |
| Rho_2[9,6] | 0.0506 | -0.5088 | 0.5862 | 0.2860 | 0.0024 | 14604.9643 | 1.0001 |
| Rho_2[9,7] | 0.0829 | -0.4678 | 0.6101 | 0.2808 | 0.0024 | 13431.4673 | 1.0003 |
| Rho_2[9,8] | 0.1070 | -0.4400 | 0.6184 | 0.2746 | 0.0024 | 13438.9093 | 1.0002 |
| Rho_2[9,9] | 1.0000 | 1.0000 | 1.0000 | 0.0000 | 0.0000 | 18533.8635 | 0.9998 |
| p[1] | 0.2039 | 0.0585 | 0.4575 | 0.1031 | 0.0011 | 8193.3531 | 1.0003 |
| p[2] | 0.2017 | 0.0560 | 0.4160 | 0.0942 | 0.0012 | 6058.0588 | 1.0003 |
| p[3] | 0.2989 | 0.1388 | 0.4951 | 0.0900 | 0.0009 | 9591.1551 | 1.0002 |
| p[4] | 0.3620 | 0.2186 | 0.5450 | 0.0796 | 0.0007 | 13355.5475 | 1.0001 |
| p[5] | 0.2350 | 0.0477 | 0.5330 | 0.1265 | 0.0010 | 14788.1811 | 1.0000 |
| p[6] | 0.1149 | 0.0138 | 0.4759 | 0.1161 | 0.0010 | 13911.5342 | 1.0001 |
| p[7] | 0.0958 | 0.0043 | 0.4355 | 0.1162 | 0.0009 | 16726.8298 | 1.0001 |
| p[8] | 0.2200 | 0.0088 | 0.7531 | 0.2000 | 0.0015 | 16997.3221 | 1.0000 |
| p[9] | 0.1873 | 0.0124 | 0.6693 | 0.1721 | 0.0013 | 17428.1349 | 0.9999 |
| p[10] | 0.1348 | 0.0235 | 0.3839 | 0.0945 | 0.0011 | 7955.4463 | 1.0000 |
| p[11] | 0.1263 | 0.0336 | 0.3334 | 0.0771 | 0.0010 | 6216.2806 | 1.0003 |
| p[12] | 0.1965 | 0.0654 | 0.3864 | 0.0834 | 0.0010 | 7427.9886 | 0.9999 |
| p[13] | 0.2500 | 0.0777 | 0.4897 | 0.1072 | 0.0012 | 8124.4675 | 1.0001 |
| p[14] | 0.1520 | 0.0222 | 0.4040 | 0.0987 | 0.0008 | 13658.1564 | 0.9999 |
| p[15] | 0.0774 | 0.0055 | 0.3698 | 0.0982 | 0.0009 | 12927.9492 | 1.0001 |
| p[16] | 0.0647 | 0.0020 | 0.3460 | 0.0965 | 0.0008 | 14865.0611 | 1.0000 |
| p[17] | 0.1565 | 0.0038 | 0.6596 | 0.1740 | 0.0014 | 14439.3858 | 1.0000 |
| p[18] | 0.1311 | 0.0053 | 0.5778 | 0.1490 | 0.0012 | 14786.2492 | 0.9999 |

Table S12: Parameter estimates for Hospitalisation ($\beta_{k\mathcal{l}}$: beta_sero_prior, $\beta_{j}$: beta_reg, $\alpha$: intercept, $\sigma_{jk\mathcal{l}}$: sigma, $\epsilon_{ijk\mathcal{l}}$: eps_vals, $P_{j}$: Rho_1 and Rho_2, $p_{jk\mathcal{l}}$: p)

## Number of Studies Contributing to Parameter Estimates

| **Parameter** | **1997-type** | **2009-type** | **Hospitalisation** |
| --- | --- | --- | --- |
| Region Effect (Americas) | 14 | 13 | 8 |
| Primary-DENV1 | 5 | 3 | 2 |
| Primary-DENV2 | 6 | 3 | 2 |
| Primary-DENV3 | 7 | 2 | 2 |
| Secondary-DENV1 | 5 | 5 | 2 |
| Secondary-DENV3 | 8 | 5 | - |
| Secondary-DENV4 | 6 | - | - |
| Unknown-DENV1 | 23 | 12 | 12 |
| Unknown-DENV2 | 25 | 13 | 13 |
| Unknown-DENV3 | 18 | 7 | 8 |
| Unknown-DENV4 | 11 | 7 | 5 |
| Intercept | 42 | 25 | 20 |

Table S13: Number of studies contributing for each parameter estimate across severity definitions. - indicates that the parameter was not estimated for the given severity definition. Separate Asian and American arms of Dussart *et al.^50^* study counted separately here.

## Individual Study Results per Scenario (1997-type Severity)

**Figure S11:** Individual study estimates and scenario summary for Americas-Primary-DENV2 with 1997-type severity

**Figure S12:** Individual study estimates and scenario summary for Americas-Primary-DENV3 with 1997-type severity

**Figure S13:** Individual study estimates and scenario summary for Americas-Secondary-DENV2 with 1997-type severity

**Figure S14:** Individual study estimates and scenario summary for Americas-Secondary-DENV3 with 1997-type severity

**Figure S15:** Individual study estimates and scenario summary for Americas-Unknown-DENV1 with 1997-type severity

**Figure S16:** Individual study estimates and scenario summary for Americas-Unknown-DENV2 with 1997-type severity

**Figure S17:** Individual study estimates and scenario summary for Americas-Unknown-DENV3 with 1997-type severity

**Figure S18:** Individual study estimates and scenario summary for Americas-Unknown-DENV4 with 1997-type severity

**Figure S19:** Individual study estimates and scenario summary for Asia-Primary-DENV1 with 1997-type severity

**Figure S20:** Individual study estimates and scenario summary for Asia-Primary-DENV2 with 1997-type severity

**Figure S21:** Individual study estimates and scenario summary for Asia-Primary-DENV3 with 1997-type severity

**Figure S22:** Individual study estimates and scenario summary for Asia-Secondary-DENV1 with 1997-type severity

**Figure S23:** Individual study estimates and scenario summary for Asia-Secondary-DENV2 with 1997-type severity

**Figure S24:** Individual study estimates and scenario summary for Asia-Secondary-DENV3 with 1997-type severity

**Figure S25:** Individual study estimates and scenario summary for Asia-Secondary-DENV4 with 1997-type severity

**Figure S26:** Individual study estimates and scenario summary for Asia-Unknown-DENV1 with 1997-type severity

**Figure S27:** Individual study estimates and scenario summary for Asia-Unknown-DENV2 with 1997-type severity

**Figure S28:** Individual study estimates and scenario summary for Asia-Unknown-DENV3 with 1997-type severity

**Figure S29:** Individual study estimates and scenario summary for Asia-Unknown-DENV4 with 1997-type severity

## Individual Study Results per Scenario (2009-type Severity)

**Figure S30:** Individual study estimates and scenario summary for Americas-Primary-DENV3 with 2009-type severity

**Figure S31:** Individual study estimates and scenario summary for Americas-Secondary-DENV1 with 2009-type severity

**Figure S32:** Individual study estimates and scenario summary for Americas-Secondary-DENV2 with 2009-type severity

**Figure S33:** Individual study estimates and scenario summary for Americas-Secondary-DENV3 with 2009-type severity

**Figure S34:** Individual study estimates and scenario summary for Americas-Unknown-DENV1 with 2009-type severity

**Figure S35:** Individual study estimates and scenario summary for Americas-Unknown-DENV2 with 2009-type severity

**Figure S36:** Individual study estimates and scenario summary for Americas-Unknown-DENV3 with 2009-type severity

**Figure S37:** Individual study estimates and scenario summary for Americas-Unknown-DENV4 with 2009-type severity

**Figure S38:** Individual study estimates and scenario summary for Asia-Primary-DENV1 with 2009-type severity

**Figure S39:** Individual study estimates and scenario summary for Asia-Primary-DENV2 with 2009-type severity

**Figure S40:** Individual study estimates and scenario summary for Asia-Secondary-DENV1 with 2009-type severity

**Figure S41:** Individual study estimates and scenario summary for Asia-Secondary-DENV2 with 2009-type severity

**Figure S42:** Individual study estimates and scenario summary for Asia-Secondary-DENV3 with 2009-type severity

**Figure S43:** Individual study estimates and scenario summary for Asia-Unknown-DENV1 with 2009-type severity

**Figure S44:** Individual study estimates and scenario summary for Asia-Unknown-DENV2 with 2009-type severity

**Figure S45:** Individual study estimates and scenario summary for Asia-Unknown-DENV3 with 2009-type severity

**Figure S46:** Individual study estimates and scenario summary for Asia-Unknown-DENV4 with 2009-type severity

## Individual Study Results per Scenario (Hospitalisation)

**Figure S47:** Individual study estimates and scenario summary for Americas-Unknown-DENV1 with hospitalisation

**Figure S48:** Individual study estimates and scenario summary for Americas-Unknown-DENV2 with hospitalisation

**Figure S49:** Individual study estimates and scenario summary for Americas-Unknown-DENV3 with hospitalisation

**Figure S50:** Individual study estimates and scenario summary for Americas-Unknown-DENV4 with hospitalisation

**Figure S51:** Individual study estimates and scenario summary for Asia-Primary-DENV1 with hospitalisation

**Figure S52:** Individual study estimates and scenario summary for Asia-Primary-DENV2 with hospitalisation

**Figure S53:** Individual study estimates and scenario summary for Asia-Primary-DENV3 with hospitalisation

**Figure S54:** Individual study estimates and scenario summary for Asia-Secondary-DENV1 with hospitalisation

**Figure S55:** Individual study estimates and scenario summary for Asia-Secondary-DENV2 with hospitalisation

**Figure S56:** Individual study estimates and scenario summary for Asia-Unknown-DENV1 with hospitalisation

**Figure S57:** Individual study estimates and scenario summary for Asia-Unknown-DENV2 with hospitalisation

**Figure S58:** Individual study estimates and scenario summary for Asia-Unknown-DENV3 with hospitalisation

**Figure S59:** Individual study estimates and scenario summary for Asia-Unknown-DENV4 with hospitalisation

## Hospitalisation Results

**Figure S60:** Estimated Log odds ratios for hospitalisation between different prior exposure-serotype combinations (with odds ratios in the table). Estimates where the 95% CrI of the OR is entirely below or above 1 were deemed to be statistically significant.

## Prior Sensitivity Analysis

We ran a prior sensitivity analysis to check the sensitivity of our main findings and the width of prediction intervals (PIs) to prior distributions. Instead of the prior distributions in Table S1, we set the prior distributions for $\alpha$, $\beta_{j}$, $\beta_{k\mathcal{l}}$, and $\sigma_{jk\mathcal{l}}$ to $N\left( 0,1 \right)$, with a $[0,\infty)$ constraint for $\sigma_{jk\mathcal{l}}$. While estimates of the mean proportion of severe dengue among symptomatic cases were similar (e.g., for secondary DENV3 in the Americas with 1997-type severity, main analysis: 23·76% [95% CrI: 12·81 to 38·24%], prior sensitivity analysis: 23·06% [95% CrI: 13·83 to 33.31%]), PIs were generally narrower (main analysis: [95% PI: 2·74 to 79·17%], prior sensitivity analysis: [95% PI: 6·45 to 54·13%] for secondary DENV3 in the Americas with 1997-type severity), as shown in Figure S61. Notably, the main findings of this prior sensitivity analysis match those from main analysis: there were no significant differences between serotypes when conditioning on the same prior exposure status (Figure S62 to Figure S64).

**Figure S61**: Forest plot of pooled estimates of the proportion of severe cases ($p_{jk\mathcal{l}}$) with corresponding prediction intervals according to the three severity definitions for the prior sensitivity analysis

**Figure S62**: Estimated log odds ratios between different prior exposure-serotype combinations (with odds ratios in the table) for the prior sensitivity analysis (using 1997-type severity). Estimates where the 95% CrI of the OR is entirely below or above 1 were deemed to be statistically significant.

**Figure S63**: Estimated log odds ratios between different prior exposure-serotype combinations (with odds ratios in the table) for the prior sensitivity analysis (using 2009-type severity). Estimates where the 95% CrI of the OR is entirely below or above 1 were deemed to be statistically significant.

**Figure S64**: Estimated log odds ratios between different prior exposure-serotype combinations (with odds ratios in the table) for the prior sensitivity analysis (for hospitalisation). Estimates where the 95% CrI of the OR is entirely below or above 1 were deemed to be statistically significant.

## Cohort Study Sensitivity Analysis

Most central estimates from the cohort study sensitivity analysis sat between the central Asian and American estimates from the main analysis (Figure S65). Relaxation of constraints on the minimum number of cases required for analysis inclusion allowed for an estimate for the proportion of severe cases due to primary DENV4 (1·56% [95% CrI: 0·10 to 5·32%]), which makes it the least severe prior exposure-serotype combination (with no DHF/DSS found in either of the two studies reporting primary DENV4 cases).^28,45^ Sensitivity analysis estimates for the proportion of severe secondary DENV4 cases (10·97% [95% CrI: 7·92 to 14·49%]) were also smaller than those from the main analysis (Asia: 26·00% [95% CrI: 9·80 to 48·94%], Americas: 15·80% [95% CrI: 4·67 to 34·84%]).

**Figure S65:** Pooled estimates from cohort sub-analysis using data from Narvaez *et al.* 2025 (Nicaragua),^45^ Stephens *et al.* 2002 (Thailand),^23^ and Fried *et al.* 2010 (Thailand)^28^ with versus model estimates in Asia and the Americas from the main analysis.

In contrast to the results of the main analysis, there was a statistically significant difference between all prior exposure-serotype pairs except for primary DENV1 and 2, secondary DENV2 and 3, and secondary DENV4 versus primary DENV1, 2, or 3 (Figure S66). This means that in the cohort study sensitivity analysis, there were statistically significant differences between serotypes with the same prior exposure status, with DENV2 and 3 being the most severe secondary infections, and DENV3 being the most severe primary infection.

**Figure S66:** Estimated log odds ratios between different prior exposure-serotype combinations (with odds ratios in the table) from the cohort study sub-analysis (using 1997-type severity). Estimates where the 95% CrI of the OR is entirely below or above 1 were deemed to be statistically significant.

## Comparison with Clinical Trial Data

To validate our results, we compared the pooled estimates of the proportion of symptomatic cases resulting in hospitalisation in Asia and the Americas for unknown prior exposure to the proportion of virologically confirmed dengue (VCD) hospitalisations reported in the placebo arm of the TAK-003 clinical trial at 57 months since the first dose (Figure S67).^76^ Excluding results from Sri Lanka, which had higher rates of hospitalisation compared to other countries, the proportion of hospitalised cases observed during the trial fell between the central estimates for DENV1 and DENV2 obtained from the meta-analysis. This is expected as excluding Sri Lanka, the trial involved cases from Asian (Philippines, Thailand) and American (Brazil, Colombia, Dominican Republic, Nicaragua, and Panama) countries.^76^ The observed proportion of hospitalised VCD for DENV4, when Sri Lanka (which only saw 1 case) was included, also fell between the central Asian and American estimates from the review. However, for DENV3, the mean pooled estimates (36·20% in Asia and 25·00% in the Americas) were larger than the observed proportion of hospitalisations from the clinical trial (15·93% including Sri Lanka and 12·96% excluding it).

**Figure S67:** Proportion of VCD cases from the placebo arm who were hospitalised during the TAK-003 clinical trials (57 months since the first dose) versus pooled proportion of hospitalised cases per serotype with unknown prior exposure status from the meta-analysis^76^ **–** bars show 95% exact binomial confidence intervals (CIs) for clinical trial data and 95% CrIs for pooled estimates.

## Excluding Unknown Prior Exposure

We re-ran the analysis for all three severity definitions excluding all cases where prior exposure status was unknown to account for potential masking of serotype-specific effects by these cases. This resulted in 11 studies for 1997-type severity, 9 for 2009-type severity, and 3 for hospitalisation. Findings from this sensitivity analysis generally aligned with those from the main analysis (Figure S68), where DENV3 in Asia was the most severe primary and secondary infection for 1997-type severity (proportion DHF/DSS: 19·73% [95% CrI: 11·20 to 29·27%] for primary and 36·12% [95% CrI: 21·07 to 51·82%] for secondary infections). DENV2 in the Americas also remained the most severe primary and secondary infection for 2009-type severity (proportion SD: 25·51% [95% CrI: 3·06 to 68·69%] for primary and 16·61% [95% CrI: 3·68 to 41·42%] for secondary infections). However, exclusion of cases with unknown prior exposure meant that only 3 studies in Asia were used for the analysis of hospitalisation, with none in the Americas. Because of this, proportions for the Americas were not well-estimated, with credible intervals spanning almost the whole probability range.

Figure S69 compares the statistical significance of odds ratios (ORs) between different pairs of prior exposure-serotype combinations for 1997-type severity. The only difference when excluding cases with unknown prior exposure was that primary DENV2 was found to be significantly less severe than secondary DENV1 (OR: 0·23 [95% CrI: 0·01 to 0·93] when excluding unknown prior exposure versus 0·25 [95% CrI: 0·01 to 1·08] in the main analysis). However, conditioning on the same prior exposure status, there was still no statistically significant difference between serotypes and no significant differences between any pair of prior exposure-serotype combinations for 2009-type and hospitalisation severity.

The difference in severity between the Americas and Asia was no longer statistically significant when cases with unknown prior exposure were excluded (OR: 0·76 [95% CrI: 0·32 to 1·75] versus 0·53 [95% CrI: 0·27 to 0·91] in the main analysis). We hypothesise that the difference between regions in the main analysis may reflect differences in underlying demographics or population-level immunity profiles resulting in differing proportions of secondary infections among those reported with unknown prior exposure status.

Figure S68: Forest plot of pooled estimates of the proportion of severe cases $\left( p_{jk\mathcal{l}} \right)$ for three severity definitions together with estimated heterogeneity $\tau$ and $I^{2}$ when excluding all cases with unknown prior exposure status

Figure S69: Pairwise comparison of prior exposure-serotype effects ($\beta_{k\mathcal{l}})$ and their statistical significance based on 95% CrIs across the main model (Main) and the model excluding cases with unknown prior exposure status (Excl. Unknown) for 1997-type severity.

## Excluding Influential Studies

To check the robustness of serotype-specific estimates of dengue severity to the most influential studies, we examined the role that these studies play in our analysis of 1997-type severity. We defined influential in terms of the contribution of an individual study to the pooled data set and identified the top 3 studies contributing the most cases with known prior exposure status. These were studies by Narvaez *et al*. 2025^45^ (1752 cases), Fried *et al*. 2010^28^ (448 cases) and Sabchareon *et al.* 2012^18^ (332 cases). We refitted the model for 1997-type severity, individually excluding each of these three studies (thus refitting the model three times).

We found that exclusion of data from Narvaez *et al.* 2025 led to a statistically significant difference between the least and most severe serotypes in primary infection, DENV2 and 3, respectively (OR: 0·14 [95% CrI: 0·00 to 0·74]), as shown in Figure S73. However, excluding data from Narvaez *et al.* 2025 also meant that estimates of primary DENV2 and 3 were based solely on studies from Asia, making this result less generalisable. Individual exclusion of Fried *et al.* and Sabchareon *et al.* did not change our conclusions regarding serotype- or region-specific differences in severity.

Figure S70 to Figure S72 show pooled estimates of the proportion of severe cases according to 1997-type severity when excluding these three studies individually. Across all three exclusions, DENV3 in Asia was, on average, still the most severe primary and secondary infection. However, excluding Fried *et al.* meant that DENV1 was the least severe primary infection, as opposed to DENV2 in the main analysis, though the pooled proportions were relatively similar (4·66% [95% CrI: 0·31 to 21·35%] versus 5·10 [95% CrI: 0·32 to 15·56%] for primary DENV1 and 2 in the Americas, respectively), as shown in Figure S71. Of the studies reporting secondary DENV3 in Asia, Sabchareon *et al.* had the smallest observed proportion of severe cases (8/54), which meant its exclusion (Figure S72) increased the pooled estimate of the proportion with DHF/DSS (43·74% [95% CrI: 32·38 to 54·02%] for Asia-Secondary-DENV3 when excluding Sabchareon *et al.* versus 37·61% [95% CrI: 24·19 to 52·32%] in the main analysis). However, this did not change the overall severity of serotypes relative to each other in primary or secondary infections.

Figure S70: Forest plot of pooled estimates of the proportion of severe cases $\left( p_{jk\mathcal{l}} \right)$ for 1997-type severity when excluding Narvaez *et al.^45^*

Figure S71: Forest plot of pooled estimates of the proportion of severe cases $\left( p_{jk\mathcal{l}} \right)$ for 1997-type severity when excluding Fried *et al.^28^*

Figure S72: Forest plot of pooled estimates of the proportion of severe cases $\left( p_{jk\mathcal{l}} \right)$ for 1997-type severity when excluding Sabchareon *et al.^18^*

Figure S73: Pairwise comparison of serotype effects ($\beta_{k\mathcal{l}})$ and their statistical significance based on 95% CrIs across different data sets for 1997-type severity. Main refers to the model including all available data, the following three columns exclude one of the top three influential studies, while the last column refers to the result when excluding all cases with unknown prior exposure status.

Stricter De-duplication

We ran an additional sensitivity analysis excluding potential duplicate data even if duplication was unclear, retaining only the most complete study on the same cohort. The analyses for each severity definition were run independently (i.e. there could be studies from the same institution with overlapping study periods but if one gives data for 1997-type severity and the other for 2009-type severity, then there is no duplication as analyses are separate). Table S14 shows a summary of the additional exclusions for 1997-type and 2009-type severity (no additional exclusions made for hospitalisation).

| **Studies** | **Country** |
| --- | --- |
| **1997-type severity** | |
| Dewi 2014^10^ | Indonesia |
| Nainggolan 2023^43^ | Indonesia |
| Reyes 2010^24^ | Nicaragua |
| Myint 2006^8^ | Thailand |
| Stephens 2002^23^ | Thailand |
| Salje 2018^38^ | Thailand |
| **2009-type severity** | |
| Yung 2015^19^ | Singapore |
| Ta 2019^54^ | Vietnam |

Table S14: Studies additionally excluded for sensitivity analysis

Figure S74 shows the pooled estimates $p_{jk\mathcal{l}}$ with these additional study exclusions, while Figure S75 and Figure S76 show estimated log ORs for each possible pair of prior exposure-serotype combination for 1997-type and 2009-type severity, respectively. Findings of this sensitivity analysis are generally consistent with the main analysis: there were no statistically significant differences between serotypes when controlling for the same prior exposure status. However, we found that after these additional exclusions, the difference between the Americas and Asia with 1997-type severity was no longer statistically significant (OR: 0·71 [95% CrI: 0·35 to 1·32]), mirroring the results of SI: Excluding Unknown Prior Exposure.

Figure S74: Forest plot of pooled estimates of the proportion of severe cases ($p_{jk\mathcal{l}}$) according to 1997-type and 2009-type severity after additional study exclusions were made (Table S14)

Figure S75: Estimated log odds ratios for 1997-type severity between different prior exposure-serotype combinations (with odds ratios in the table) after additional study exclusions. Estimates where the 95% CrI of the OR is entirely below or above 1 were deemed to be statistically significant.

Figure S76: Estimated log odds ratios for 2009-type severity between different prior exposure-serotype combinations (with odds ratios in the table) after additional study exclusions. Estimates where the 95% CrI of the OR is entirely below or above 1 were deemed to be statistically significant.

# References

1.	Hoy D, Brooks P, Woolf A, et al. Assessing risk of bias in prevalence studies: modification of an existing tool and evidence of interrater agreement. *J Clin Epidemiol* 2012; **65**(9): 934–9.

2.	World Health Organization. Dengue haemorrhagic fever : diagnosis, treatment, prevention and control. Geneva: World Health Organization; 1997.

3.	World Health Organization. Dengue guidelines for diagnosis, treatment, prevention and control : new edition. World Health Organization; 2009.

4.	Poeranto S, Sutaryo S, Josef HK, Juffrie M. A relationship between dengue virus serotype and the clinical severity in paediatric patients from Gondokusuman region, Yogyakarta between 1995 and 1999. *Pediatria i Medycyna Rodzinna* 2016; **12**(3): 318–25.

5.	Osman O, Fong MY, Devi S. A preliminary study of dengue infection in Brunei. *Jpn J Infect Dis* 2007; **60**(4): 205–8.

6.	Burattini MN, Lopez LF, Coutinho FA, et al. Age and regional differences in clinical presentation and risk of hospitalization for dengue in Brazil, 2000-2014. *Clinics (Sao Paulo, Brazil)* 2016; **71**(8): 455–63.

7.	Sharp TM, Hunsperger E, Santiago GA, et al. Virus-specific differences in rates of disease during the 2010 Dengue epidemic in Puerto Rico. *PLoS Negl Trop Dis* 2013; **7**(4): e2159.

8.	Myint KS, Endy TP, Mongkolsirichaikul D, et al. Cellular immune activation in children with acute dengue virus infections is modulated by apoptosis. *J Infect Dis* 2006; **194**(5): 600–7.

9.	Saha K, Ghosh M, Firdaus R, et al. Changing pattern of dengue virus serotypes circulating during 2008-2012 and reappearance of dengue serotype 3 may cause outbreak in Kolkata, India. *J Med Virol* 2016; **88**(10): 1697–702.

10.	Dewi BE, Nainggolan L, Putri DH, et al. Characterization of dengue virus serotype 4 infection in Jakarta, Indonesia. *Southeast Asian J Trop Med Public Health* 2014; **45**(1): 53–61.

11.	Montoya Y, Holechek S, Caceres O, et al. Circulation of dengue viruses in North-Western Peru, 2000-2001. *Dengue Bulletin* 2003; **27**: 52–62.

12.	Dewi BE, Nainggolan L, Sudiro TM, Chenderawasi S, Goentoro PL, Sjatha F. Circulation of various dengue serotypes in a community-based study in Jakarta, Indonesia. *Jpn J Infect Dis* 2021; **74**(1): 17–22.

13.	Wardhani P, Aryati A, Yohan B, et al. Clinical and virological characteristics of dengue in Surabaya, Indonesia. *PLoS One* 2017; **12**(6): e0178443.

14.	Harris E, Videa E, Perez L, et al. Clinical, epidemiologic, and virologic features of dengue in the 1998 epidemic in Nicaragua. *Am J Trop Med Hyg* 2000; **63**(1-2): 5–11.

15.	Kerdpanich P, Kongkiatngam S, Buddhari D, et al. Comparative analyses of historical trends in confirmed dengue illnesses detected at public hospitals in Bangkok and Northern Thailand, 2002-2018. *Am J Trop Med Hyg* 2021; **104**(3): 1058–66.

16.	Guilarde AO, Turchi MD, Siqueira JB, Jr., et al. Dengue and dengue hemorrhagic fever among adults: clinical outcomes related to viremia, serotypes, and antibody response. *J Infect Dis* 2008; **197**(6): 817–24.

17.	World Health Organization. Dengue fever/dengue haemorrhagic fever: Puerto Rico. *Wkly Epidemiol Rec* 1987; **62**(28): 206–8.

18.	Sabchareon A, Sirivichayakul C, Limkittikul K, et al. Dengue Infection in Children in Ratchaburi, Thailand: A Cohort Study. I. Epidemiology of Symptomatic Acute Dengue Infection in Children, 2006–2009. *PLoS Negl Trop Dis* 2012; **6**(7): e1732.

19.	Yung C-F, Lee K-S, Thein T-L, et al. Dengue serotype-specific differences in clinical manifestation, laboratory parameters and risk of severe disease in adults, Singapore. *Am J Trop Med Hyg* 2015; **92**(5): 999–1005.

20.	Dumre SP, Bhandari R, Shakya G, et al. Dengue Virus Serotypes 1 and 2 Responsible for Major Dengue Outbreaks in Nepal: Clinical, Laboratory, and Epidemiological Features. *Am J Trop Med Hyg* 2017; **97**(4): 1062–9.

21.	Chakravarti A, Kumar A, Matlani M. Displacement of dengue virus type 3 and type 2 by dengue virus type 1 in Delhi during 2008. *Indian J Med Microbiol* 2010; **28**(4): 412.

22.	Rao MRK, Padhy RN, Das MK. Episodes of the epidemiological factors correlated with prevailing viral infections with dengue virus and molecular characterization of serotype-specific dengue virus circulation in eastern India. *Infect Genet Evol* 2018; **58**: 40–9.

23.	Stephens HAF, Klaythong R, Sirikong M, et al. HLA-A and -B allele associations with secondary dengue virus infections correlate with disease severity and the infecting viral serotype in ethnic Thais. *Tissue Antigens* 2002; **60**(4): 309–18.

24.	Reyes M, Mercado JC, Standish K, et al. Index Cluster Study of Dengue Virus Infection in Nicaragua. *Am J Trop Med Hyg* 2010; **83**(3): 683–9.

25.	Feres VCR, Martelli CMT, Turchi MD, et al. Laboratory surveillance of dengue virus in Central Brazil, 1994-2003. *J Clin Virol* 2006; **37**(3): 179–83.

26.	Sharma A, Rajbongshi G, Alam S, et al. Molecular typing of dengue viruses circulating in Assam, India during 2016-2017. *J Vector Borne Dis* 2020; **57**(3): 249–58.

27.	Thomas L, Najioullah F, Verlaeten O, et al. Relationship between nonstructural protein 1 detection and plasma virus load in Dengue patients. *Am J Trop Med Hyg* 2010; **83**(3): 696–9.

28.	Fried JR, Gibbons RV, Kalayanarooj S, et al. Serotype-specific differences in the risk of dengue hemorrhagic fever: an analysis of data collected in Bangkok, Thailand from 1994 to 2006. *PLoS Negl Trop Dis* 2010; **4**(3): e617.

29.	Falconar AKI, Romero-Vivas CME. Simple prognostic criteria can definitively identify patients who develop severe versus non-severe dengue disease, or have other febrile illnesses. *J Clin Med Res* 2012; **4**(1): 33–44.

30.	Ocazionez RE, Cortes FM, Villar LA, Gomez SY. Temporal distribution of dengue virus serotypes in Colombian endemic area and dengue incidence. Re-introduction of dengue-3 associated to mild febrile illness and primary infection. *Mem Inst Oswaldo Cruz* 2006; **101**(7): 725–31.

31.	Kao J, Chen C, Li Z, et al. The critical role of early dengue surveillance and limitations of clinical reporting - implications for non-endemic countries. *PLoS One* 2016; **11**(8): e0160230.

32.	Kosasih H, Alisjahbana B, Nurhayati, et al. The Epidemiology, Virology and Clinical Findings of Dengue Virus Infections in a Cohort of Indonesian Adults in Western Java. *PLoS Negl Trop Dis* 2016; **10**(2): e0004390.

33.	Capeding RZ, Brion JD, Caponpon MM, et al. The incidence, characteristics, and presentation of dengue virus infections during infancy. *Am J Trop Med Hyg* 2010; **82**(2): 330–6.

34.	Calvez E, Pommelet V, Somlor S, et al. Trends of the dengue serotype-4 circulation with epidemiological, phylogenetic, and entomological insights in lao pdr between 2015 and 2019. *Pathogens* 2020; **9**(9): 1–17.

35.	Mammen MP, Pimgate C, Koenraadt CJM, et al. Spatial and Temporal Clustering of Dengue Virus Transmission in Thai Villages. *PLoS Medicine* 2008; **5**(11): e205.

36.	Trravassos da Rosa AP, Vasconcelos PFC, Trravassos da Rosa ES, et al. Dengue epidemic in Belem, Para, Brazil, 1996-97. *Emerg Infect Dis* 2000; **6**(3): 298–301.

37.	Effler PV, Pang L, Kitsutani P, et al. Dengue fever, Hawaii, 2001-2002. *Emerg Infect Dis* 2005; **11**(5): 742–9.

38.	Salje H, Cummings DAT, Rodriguez-Barraquer I, et al. Reconstruction of antibody dynamics and infection histories to evaluate dengue risk. *Nature* 2018; **557**(7707): 719–23.

39.	Fakeeh M, Zaki AM. Virologic and serologic surveillance for dengue fever in Jeddah, Saudi Arabia, 1994-1999. *Am J Trop Med Hyg* 2001; **65**(6): 764–7.

40.	Beckett CG, Kosasih H, Faisal I, et al. Early detection of dengue infections using cluster sampling around index cases. *Am J Trop Med Hyg* 2005; **72**(6): 777–82.

41.	Messer WB, Vitarana UT, Sivananthan K, et al. Epidemiology of dengue in Sri Lanka before and after the emergence of epidemic dengue hemorrhagic fever. *Am J Trop Med Hyg* 2002; **66**(6): 765–73.

42.	Dung NTP, Duyen HTL, Thuy NTV, et al. Timing of CD8+ T Cell Responses in Relation to Commencement of Capillary Leakage in Children with Dengue. *J Immunol* 2010; **184**(12): 7281–7.

43.	Nainggolan L, Dewi BE, Hakiki A, et al. Association of viral kinetics, infection history, NS1 protein with plasma leakage among Indonesian dengue infected patients. *PLoS One* 2023; **18**(5): e0285087.

44.	Jadeja BJ, Khokhar N, Patel P, Makwana N, Shrimali G. Dengue Serotype Prevalence and Laboratory Profile Correlation in a Tertiary Care Hospital in Gandhinagar. *Natl J Med Res* 2022; **12**(04): 92–6.

45.	Narvaez F, Montenegro C, Juarez JG, et al. Dengue severity by serotype and immune status in 19 years of pediatric clinical studies in Nicaragua. *PLoS Negl Trop Dis* 2025; **19**(1): e0012811.

46.	Nwe KM, Ngwe Tun MM, Myat TW, et al. Acute-phase serum cytokine levels and correlation with clinical outcomes in children and adults with primary and secondary dengue virus infection in Myanmar between 2017 and 2019. *Pathogens* 2022; **11**(5).

47.	Azevedo BP, Farias PCS, Pastor AF, et al. AA IDO1 Variant Genotype (G2431A, rs3739319) Is Associated with Severe Dengue Risk Development in a DEN-3 Brazilian Cohort. *Viral Immunol* 2019; **32**(7): 296–301.

48.	Huang SW, Tsai HP, Hung SJ, Ko WC, Wang JR. Assessing the risk of dengue severity using demographic information and laboratory test results with machine learning. *PLoS Negl Trop Dis* 2020; **14**(12): 1–19.

49.	Martins VdCA, Bastos MdS, Ramasawmy R, et al. Clinical and virological descriptive study in the 2011 outbreak of dengue in the Amazonas, Brazil. *PLoS One* 2014; **9**(6): e100535.

50.	Dussart P, Baril L, Petit L, et al. Clinical and virological study of dengue cases and the members of their households: the multinational DENFRAME project. *PLoS Negl Trop Dis* 2012; **6**(1): e1482.

51.	Mukherjee S, Saha B, Tripathi A. Clinical significance of differential serum-signatures for early prediction of severe dengue among Eastern Indian patients. *Clin Exp Immunol* 2022; **208**(1): 72–82.

52.	Racherla RG, Pamireddy ML, Mohan A, et al. Co-circulation of four dengue serotypes at South Eastern Andhra Pradesh, India: A prospective study. *Indian J Med Microbiol* 2018; **36**(2): 236–40.

53.	Ly AN, Manzanero R, Maliga A, et al. Epidemiological and clinical characteristics of acute dengue virus infections detected through acute febrile illness surveillance, Belize 2020. *Viruses* 2022; **14**(4).

54.	Ta TV, Huynh TC, Tran HT, et al. Factors predicting severe dengue in children within 72 hours of illness onset. *Arch Pharm Pract* 2019; **10**(3): 87–91.

55.	Mehta TK, Shah PD. Identification of prevalent dengue serotypes by reverse transcriptase polymerase chain reaction and correlation with severity of dengue as per the recent World Health Organization classification (2009). *Indian J Med Microbiol* 2018; **36**(2): 273–8.

56.	Rouers A, Chng MHY, Lee B, et al. Immune cell phenotypes associated with disease severity and long-term neutralizing antibody titers after natural dengue virus infection. *Cell Rep Med* 2021; **2**(5): 100278.

57.	Guerra-Gomes IC, Gois BM, Peixoto RF, et al. Molecular and clinical epidemiological surveillance of dengue virus in Paraiba, Northeast Brazil. *Rev Soc Bras Med Trop* 2017; **50**(1): 19–26.

58.	Braga C, Albuquerque Md.e F, Cordeiro MT, et al. Prospective birth cohort in a hyperendemic dengue area in Northeast Brazil: methods and preliminary results. *Cad Saude Publica* 2016; **32**(1).

59.	Gupta BP, Singh S, Kurmi R, Malla R, Sreekumar E, Manandhar KD. Re-emergence of dengue virus serotype 2 strains in the 2013 outbreak in Nepal. *Indian J Med Res* 2015; **142**(7 (Suppl.)): 1–6.

60.	Perdomo-Celis F, Salgado DM, Narvaez CF. Selective dysfunction of subsets of peripheral blood mononuclear cells during pediatric dengue and its relationship with clinical outcome. *Virology* 2017; **507**: 11–9.

61.	Nguyen MT, Ho TN, Nguyen VVC, et al. An evidence-based algorithm for early prognosis of severe dengue in the outpatient setting. *Clin Infect Dis* 2017; **64**(5): 656–63.

62.	Estofolete CF, Versiani AF, Dourado FS, et al. Influence of previous Zika virus infection on acute dengue episode. *PLoS Negl Trop Dis* 2023; **17**(11): e0011710.

63.	Fiora MB, Gonzalvez ML, Aguirre JP, et al. Observational study of clinical, epidemiological, and laboratory characteristics of pediatric patients with dengue in the city of Córdoba. *Arch Argent Pediatr* 2024; **122**(1): e202202972.

64.	Odio CD, Daag JV, Crisostomo MV, et al. Dengue virus IgG and neutralizing antibody titers measured with standard and mature viruses are protective. *Nat Commun* 2025; **16**(1): 191.

65.	Burgos F, Ramírez-Orrego L, Rojas LM, Tapia-Limonchi R, Gutierrez C, Chenet SM. Dengue virus serotypes 1 and 2 responsible for dengue burden in Amazonas: epidemiological and clinical features. *Am J Trop Med Hyg* 2025; **112**(1): 189–93.

66.	Hanna JN, Ritchie SA, Phillips DA, et al. An epidemic of dengue 3 in far north Queensland, 1997-1999. *Med J Aust* 2001; **174**(4): 178–82.

67.	Santiago GA, Sharp TM, Rosenberg E, et al. Prior dengue virus infection is associated with increased viral load in patients infected with dengue but not Zika virus. *Open Forum Infect Dis* 2019; **6**(7): ofz320.

68.	Aubry M, Mapotoeke M, Teissier A, et al. Dengue virus serotype 2 (DENV-2) outbreak, French Polynesia, 2019. *Euro Surveill* 2019; **24**(29).

69.	Duyen HTL, Ngoc TV, Ha DT, et al. Kinetics of plasma viremia and soluble nonstructural protein 1 concentrations in dengue: differential effects according to serotype and immune status. *J Infect Dis* 2011; **203**(9): 1292–300.

70.	Gomez Dantes H, Koopman JS, Addy CL, et al. Dengue epidemics on the Pacific Coast of Mexico. *Int J Epidemiol* 1988; **17**(1): 178–86.

71.	Hanna JN, Ritchie SA, Merritt AD, et al. Two contiguous outbreaks of dengue type 2 in north Queensland. *Med J Aust* 1998; **168**(5): 221–5.

72.	Al Balushi L, Al Kalbani M, Al Manji A, et al. A second local dengue fever outbreak: A field experience from Muscat Governorate in Oman, 2022. *IJID Reg* 2023; **7**: 237–41.

73.	Rahim R, Hasan A, Phadungsombat J, et al. Genetic Analysis of Dengue Virus in Severe and Non-Severe Cases in Dhaka, Bangladesh, in 2018–2022. *Viruses* 2023; **15**(5): 1144.

74.	Rodriguez DM, Madewell ZJ, Torres JM, et al. Epidemiology of Dengue — Puerto Rico, 2010–2024. *Morb Mortal Wkly Rep* 2024; **73**(49): 1112–7.

75.	Peters JL, Sutton AJ, Jones DR, Abrams KR, Rushton L. Comparison of Two Methods to Detect Publication Bias in Meta-analysis. *JAMA* 2006; **295**(6): 676–80.

76.	Tricou V, Yu D, Reynales H, et al. Long-term efficacy and safety of a tetravalent dengue vaccine (TAK-003): 4·5-year results from a phase 3, randomised, double-blind, placebo-controlled trial. *Lancet Glob Health* 2024; **12**(2): e257–e70.
